# Hippocampal low-frequency stimulation prevents seizure generation in a mouse model of mesial temporal lobe epilepsy

**DOI:** 10.7554/eLife.54518

**Published:** 2020-12-22

**Authors:** Enya Paschen, Claudio Elgueta, Katharina Heining, Diego M Vieira, Piret Kleis, Catarina Orcinha, Ute Häussler, Marlene Bartos, Ulrich Egert, Philipp Janz, Carola A Haas

**Affiliations:** 1 Experimental Epilepsy Research, Department of Neurosurgery, Medical Center - University of Freiburg, Faculty of MedicineFreiburgGermany; 2 Faculty of Biology, University of FreiburgFreiburgGermany; 3 Systemic and Cellular Neurophysiology, Institute for Physiology I, Faculty of Medicine, University of FreiburgFreiburgGermany; 4 Biomicrotechnology, Department of Microsystems Engineering – IMTEK, Faculty of Engineering, University of FreiburgFreiburgGermany; 5 Bernstein Center Freiburg, University of FreiburgFreiburgGermany; 6 Center for Basics in NeuroModulation, Faculty of Medicine, University of FreiburgFreiburgGermany; Stanford University School of MedicineUnited States; Stanford University School of MedicineUnited States

**Keywords:** dentate gyrus, kainate, optogenetics, deep brain stimulation, channelrhodopsin, Mouse

## Abstract

Mesial temporal lobe epilepsy (MTLE) is the most common form of focal, pharmacoresistant epilepsy in adults and is often associated with hippocampal sclerosis. Here, we established the efficacy of optogenetic and electrical low-frequency stimulation (LFS) in interfering with seizure generation in a mouse model of MTLE. Specifically, we applied LFS in the sclerotic hippocampus to study the effects on spontaneous subclinical and evoked generalized seizures. We found that stimulation at 1 Hz for 1 hr resulted in an almost complete suppression of spontaneous seizures in both hippocampi. This seizure-suppressive action during daily stimulation remained stable over several weeks. Furthermore, LFS for 30 min before a pro-convulsive stimulus successfully prevented seizure generalization. Finally, acute slice experiments revealed a reduced efficacy of perforant path transmission onto granule cells upon LFS. Taken together, our results suggest that hippocampal LFS constitutes a promising approach for seizure control in MTLE.

## Introduction

Mesial temporal lobe epilepsy (MTLE) represents the most common form of acquired epilepsy in adults. MTLE is thought to arise from an initial precipitating insult in early childhood, such as *status epilepticus* (SE), complex febrile seizures, or head trauma (Engel, 2001). The most frequent histopathological hallmark of MTLE is hippocampal sclerosis, which is characterized by neuronal cell loss and gliosis (Blümcke et al., 2013). In addition, it is often associated with granule cell dispersion (GCD) and mossy fiber sprouting (Thom, 2014). MTLE is of particular clinical interest since it is frequently resistant to pharmacological treatment (Engel, 2001). Hence, surgical removal of the seizure focus is an effective therapeutic intervention for many MTLE patients (Englot and Chang, 2014). However, for patients with multiple seizure foci or for those at risk of resection-related impairments, this treatment is not an option.

One alternative for these patients is electrical deep brain stimulation (DBS) which often targets either the hippocampus or the anterior thalamic nucleus (Li and Cook, 2018). Complementary to pharmacological treatment, DBS at high frequencies (HFS, 130–200 Hz at 1–5 V) (Laxpati et al., 2014; Li and Cook, 2018), either as an open-loop (Boëx et al., 2011; Tellez-Zenteno et al., 2006; Velasco et al., 2007) or a closed-loop stimulation, especially the Responsive Neuro Stimulation (RNS) System (Bergey et al., 2015; Geller et al., 2017; Nair et al., 2020), is currently in use to alleviate intractable seizures.

In MTLE with hippocampal sclerosis, however, the efficacy of HFS is rather variable between patients (Boëx et al., 2011; Velasco et al., 2007). This is in line with the hypothesis that neuronal loss and/or altered electrical resistance in sclerotic neural tissue impair the efficacy of HFS since stimulation can only be successful when targeting a sufficiently preserved network (Cuéllar-Herrera et al., 2004; Velasco et al., 2007). MTLE patients with hippocampal sclerosis may therefore require specific stimulation parameters to achieve seizure control.

Interestingly, low-frequency stimulation (LFS) at 5 Hz was effective in MTLE patients in small cohort studies, including those with hippocampal sclerosis (Koubeissi et al., 2013; Lim et al., 2016). From a technical perspective, LFS would be favorable for clinical implementation due to its low duty cycle, resulting in less electric current injection and longer battery life. For a systematic assessment of seizure-suppressive effects of LFS in relation to disease parameters, studies in translational animal models are crucial.

Optogenetic stimulation offers cell- or pathway-specific modulation of neuronal activity and has been successfully applied to alleviate seizure burden in several rodent MTLE models (Krook-Magnuson and Soltesz, 2015; Zhao et al., 2015). These optogenetic approaches targeting the hippocampus were either based on the inhibition of excitatory neurons or on the recruitment of inhibitory interneurons (Kim et al., 2020; Kokaia et al., 2013; Krook-Magnuson et al., 2013; Ladas et al., 2015; Ledri et al., 2014; Lu et al., 2016). In MTLE models and patients with strong hippocampal sclerosis, however, pyramidal cells and GABAergic interneurons are strongly diminished and therefore would be difficult to target (Bouilleret et al., 1999; Maglóczky and Freund, 2005; Marx et al., 2013; Thom, 2014).

In the present study, we applied optogenetic LFS (oLFS) to systematically investigate the efficacy of different stimulation frequencies in seizure interference. We used the intrahippocampal kainate (KA) mouse model, which replicates the major hallmarks of human MTLE pathology, comprising the emergence of spontaneous recurrent seizures and robust unilateral hippocampal sclerosis (Bouilleret et al., 1999). In this model, dentate granule cells (DGCs) with their entorhinal inputs, i.e. the perforant path (Froriep et al., 2012; Janz et al., 2017a), and CA2 pyramidal cells are preserved (Häussler et al., 2016). Using different KA concentrations, we modified the severity of hippocampal sclerosis and applied oLFS to stimulate entorhinal afferents in the diseased hippocampus *in vivo* while recording local field potentials (LFP). We then assessed the responses of individual DGCs to oLFS *in vitro* by patch-clamp recordings. In addition, we probed the translational value of LFS by applying electrical LFS (eLFS) *in vivo* over several weeks. We present evidence that LFS is highly effective in preventing both subclinical epileptiform activity and behavioral seizures in experimental MTLE with severe hippocampal sclerosis.

## Results

### Modification of the intrahippocampal KA mouse model

**Figure 1.**
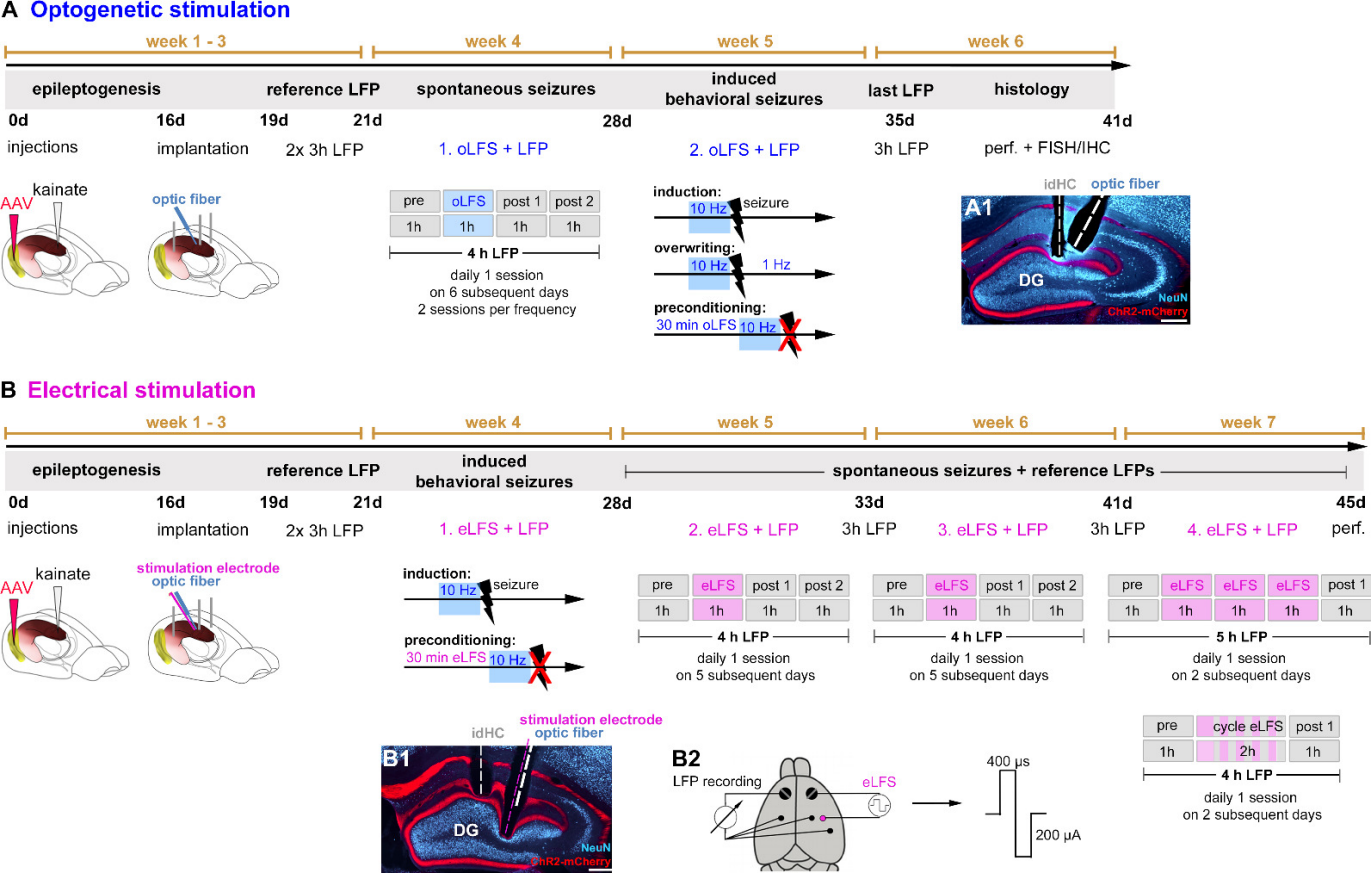
Experimental design for *in vivo* LFS. Animals received intrahippocampal KA and a channelrhodopsin 2 (ChR2)-carrying virus into the entorhinal cortex to trigger epileptogenesis and the expression of ChR2-mCherry in entorhinal afferent fibers. After 16 days post-injection, recording electrodes, and (**A**) an optic fiber or (**B**) an optic fiber combined with a stimulation electrode were implanted. Following recovery from implantations, reference LFPs were recorded on 2 consecutive days for 3 hr each. (**A**) In the first group of experiments, the effect of oLFS on spontaneously occurring epileptiform activity was tested (week 4) in 4-hr recording sessions. A session consisted of 1 hr of ‘pre’ stimulus recording, followed by 1 hr of ‘oLFS’ pulses and 2 hr of post-stimulus recordings (‘post 1’ and ‘post 2’). Three different oLFS frequencies (1, 0.5, or 0.2 Hz) were applied on successive days in each animal (two sessions per animal). Next, generalized seizures were induced by optogenetic (10 Hz) stimulation. To test the effects of oLFS on seizures, oLFS (1 and 0.5 Hz) was applied either immediately after (overwriting) or before the pro-convulsive (10 Hz) stimulation (preconditioning) (week 5). (**B**) The second group of *in vivo* experiments assessed the effects of eLFS on epileptiform activity. First, we tested the effects of eLFS on optogenetically induced seizures (week 4, preconditioning, see above). In weeks 5 and 6, animals were stimulated daily for 1 hr (1 Hz, eLFS) following the same ‘pre’, ‘eLFS’,’ post 1’, post 2’ paradigm, as described above. In week 7, animals were stimulated twice (on 2 different days) over 3 hr continuously and twice in an on-off ‘cycle’ paradigm: after initial 30 min eLFS, eLFS stimulation was turned off for 10 min and then turned on again for 10 min. This was repeated four times, followed by another hour LFP recording (‘post 1’). (**A1, B1**) All animals were perfused after the last LFP recording and brain sections were processed for immunohistological procedures. (**B2**) Implantation scheme for eLFS. DG, dentate gyrus; FISH, fluorescent *in situ* hybridization; IHC, immunohistochemistry; perf., perfusion.

**Figure 2.**
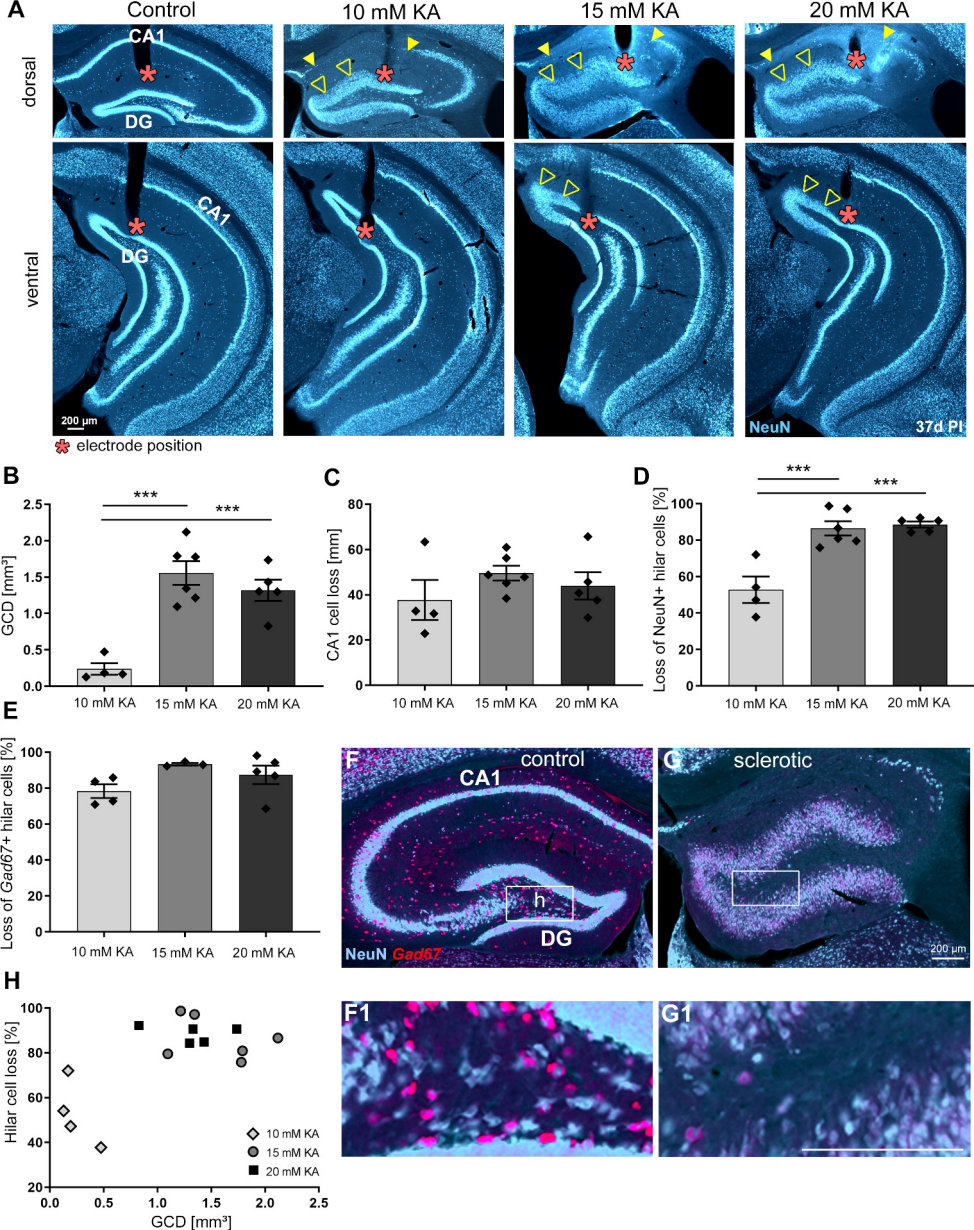
The degree of hippocampal sclerosis depends on KA concentration. (**A**) Representative NeuN-labeled sections of dorsal and ventral hippocampal regions treated with different KA concentrations at 37 days post-injection (PI). In each section, the electrode position is marked with a red asterisk. Epileptic hippocampi show GCD in the dentate gyrus (open arrowheads) and cell loss in CA1 (the region between filled arrowheads). Comparing KA concentration groups with respect to different markers of hippocampal sclerosis by quantification of (**B**) GCL volume of dispersed regions (i.e. GCD), (**C**) total length of cell loss in CA1, (**D**) % loss of NeuN^+^ hilar cells, and (**E**) loss of *Gad67^+^* hilar interneurons in the sclerotic vs. non-sclerotic hippocampus (i.e. (**G, G1**) ipsilateral vs (**F, F1**) contralateral). One-way ANOVA; Tukey’s multiple comparison test; *p<0.05, **p<0.01 and ***p<0.001. All values are given as mean ± standard error of the mean (SEM). (**H**) Animals injected with higher KA concentrations (15 and 20 mM KA) display stronger hilar cell loss along with a higher degree of GCD. Scale bars 200 µm.

**Figure 3.**
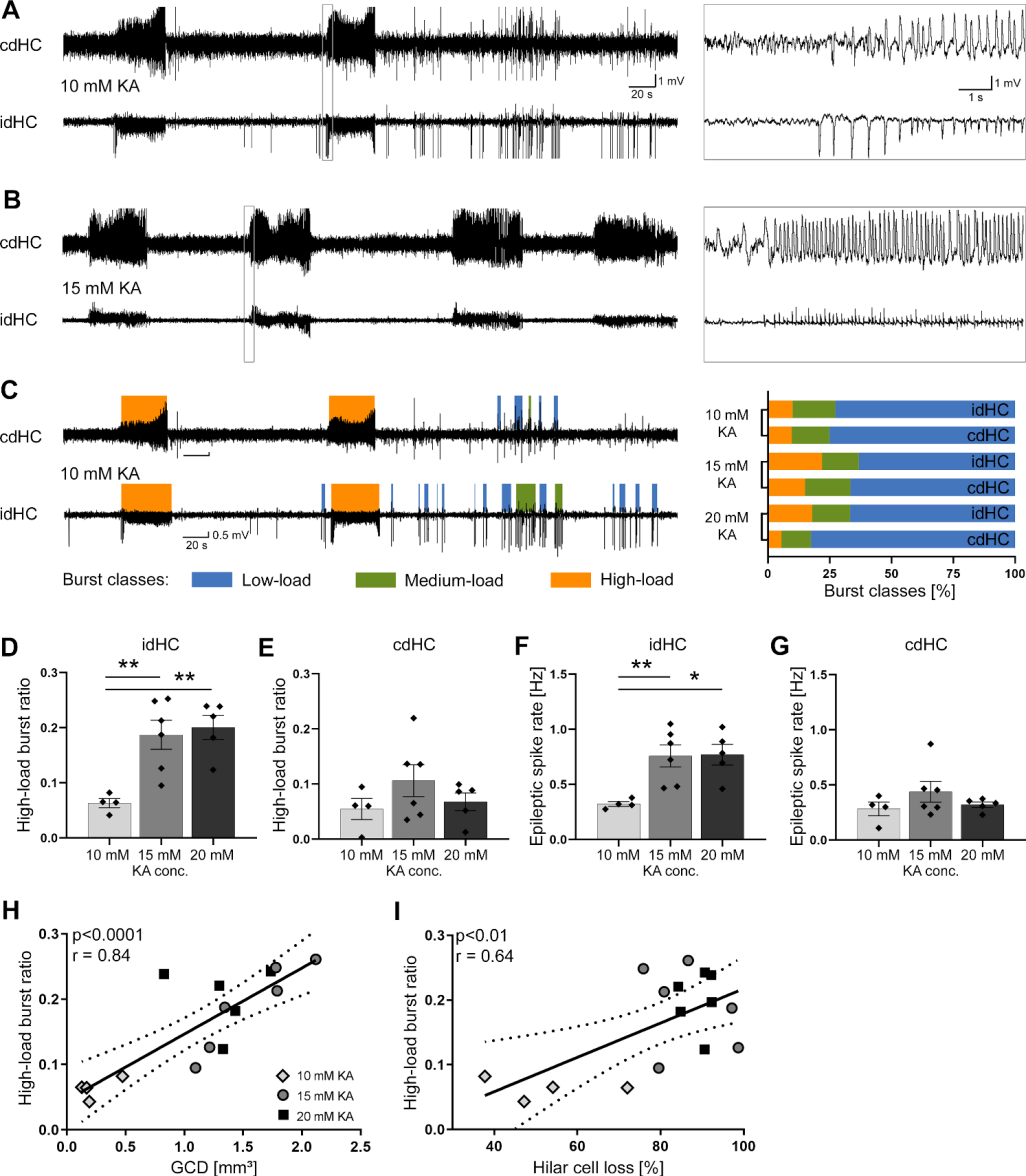
Variable severity of epileptiform activity elicited by different KA concentrations. (**A, B**) Representative LFP traces for the 10 mM and 15 mM KA group (20 mM not shown) showing spontaneous epileptiform activity in the cdHC and idHC. (**C**) Automatic classification of epileptiform activity into low-load (blue), medium-load (green) and high-load bursts (orange). We used a custom algorithm as illustrated in Figure 3—figure supplement 1. In the 20 mM KA group, the percentage of high-load bursts in the cdHC is decreased, whereas for the 10 mM and 15 mM KA group the percentage of the burst classes is similar in both hippocampi. (**D–G**) Injections of 15 and 20 mM KA lead to an increased high-load burst ratio and a higher epileptic spike rate in the idHC but not in the cdHC. All values are given as mean ± SEM. Source data is provided in Figure 3—source data 1. (**I, J**) The high-load burst ratio is positively correlated with GCD and hilar NeuN^+^ cell loss ((**I**): p<0.0001, two-tailed; Pearson’s r = 0.84; (**J**): p<0.01, two-tailed; Pearson’s r = 0.64). Figure 3—source data 1.Variable severity of epileptiform activity elicited by different KA concentrations.(**a**) The occurrence of burst classes elicited by different KA concentrations. In the ipsilateral hippocampus, animals treated with high (15 and 20 mM) KA concentrations exhibit more high-load and fewer low-load events than animals treated with 10 mM KA. Compared to the ipsilateral hippocampus, animals treated with high (especially 20 mM KA) have less high-load seizures on the contralateral side. (**b**) Mean high-load burst ratio and epileptic spike rate of the different KA groups. The mean ratio of time spent in high-load bursts (mean high-load burst ratio) and epileptic spike rate were significantly lower in the idHC 10 mM KA group compared to the 15 and 20 mM KA group. The mean was calculated from the reference and ‘pre’ recording sessions. Values are given as mean ± SEM.

To achieve different degrees of hippocampal sclerosis and seizure burden as observed in human MTLE (Thom, 2014), we modified the established intrahippocampal KA mouse model by injecting three KA concentrations (10, 15, and 20 mM). To this end, we compared these KA groups at 35–40 days after KA injection with respect to GCD, cell loss in CA1 and hilus, and epileptiform activity (Figures 1A, 2 and 3).

**Figure 3—figure supplement 1.**
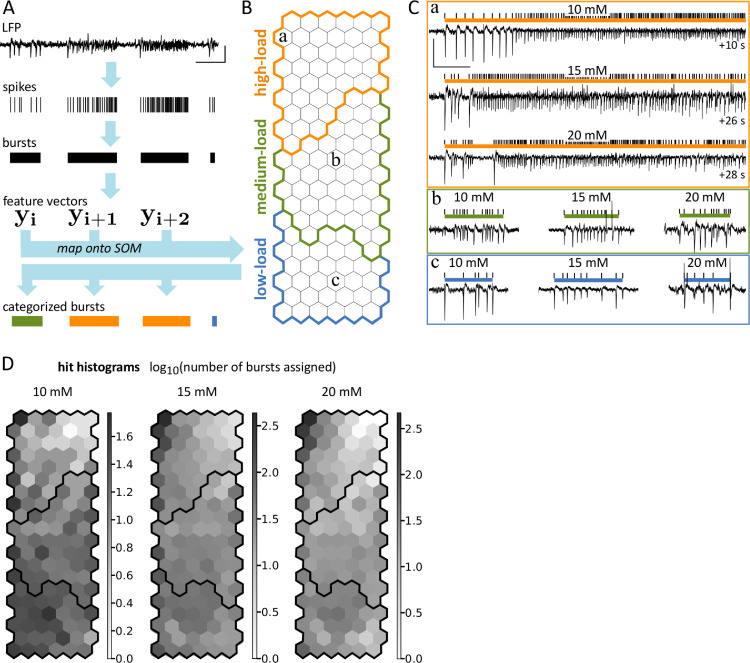
Analysis of epileptiform activity and comparison of different KA concentrations. (**A**) The workflow of analysis. Spikes were detected in LFP traces and spikes closer than 2.5 s were grouped into the same burst. For each burst, a feature vector (*y_i_*) was extracted and mapped onto the SOM obtained from a reference dataset (see panel B). Each *y_i_*was matched to the SOM’s node closest in feature space. Each burst inherited its category from this node. (**B**) SOM developed by Heining et al., 2019 was used to categorize bursts according to spike load. Nodes of the SOM are visualized as hexagons. (**C**) Exemplary bursts (ticks: detected spikes, colored bar: burst extent) matched to the hexagons marked a – c in B. For each hexagon, a representative burst from a mouse injected with 10, 15 or 20 mM KA is shown. Examples were truncated for visualization. They continued for the time indicated (+...s) with a similar activity pattern. (**D**) The normalized number of bursts (log10) matched to each node for mice injected with 10, 15 or 20 mM KA. We normalized by dividing the number of bursts by the total recording time analyzed for the respective dataset. Data shown in D is pooled across animals from all EEG and ‘pre’ recording sessions (10 mM: n = 35, 15 mM: n = 80, 20mM: n=58 sessions). Scale bars: 5 s; 4 mV.

Quantitative analysis of GCD in NeuN-stained sections revealed that the volume of the dispersed granule cell layer (GCL) was comparable between 20 and 15 mM KA but significantly smaller in the 10 mM KA group (Figure 2B, 10 mM: 0.24 ± 0.08 mm^3^; 15 mM: 1.56 ± 0.16 mm^3^; 20 mM: 1.52 ± 0.11 mm^3^, 10 mM vs 15 mM and vs. 20 mM p<0.001; n = 4; 6; 5 animals). Conversely, the loss of CA1 pyramidal cells, quantified as the total length of CA1 devoid of pyramidal cells, was similar in all groups (Figure 2C, 10 mM: 37.72 ± 8.84 mm; 15 mM: 49.57 ± 3.27 mm; 20 mM: 42.28 ± 6.44 mm; n = 4; 6; 5 animals).

The loss of NeuN+ hilar neurons in the sclerotic ipsilateral dorsal hippocampus (idHC) compared to the non-sclerotic contralateral dorsal hippocampus (cdHC) (Figure 2F,G) was significantly less pronounced in mice injected with 10 mM KA (Figure 2D, 10 mM: 52.78 ± 7.24% cell loss; 15 mM: 86.47 ± 3.90% cell loss; 20 mM: 88.54 ± 1.66% cell loss; 10 mM vs 15 mM and vs 20 mM p<0.001; n = 4; 6; 5 animals), whereas *glutamic acid decarboxylase 67* (*Gad67)* mRNA^+^ interneurons were equally lost in all groups (Figure 2E, 10 mM: 78.33 ± 3.77% cell loss; 15 mM: 93.35 ± 0.71% cell loss; 20 mM: 85.20 ± 4.35% cell loss; n = 4; 3; 5 animals). Animals injected with a higher KA dosage (15 and 20 mM), which showed greater loss of NeuN^+^ neurons in the hilus, also had a larger volume of the dispersed GCL (Figure 2H).

Next, we investigated the characteristics of spontaneous epileptiform activity in all mice using LFP recordings from electrodes in both dorsal hippocampi (idHC and cdHC, Figure 3). We used a custom algorithm as described in the Materials and methods and illustrated in the supplements (Figure 3—figure supplement 1) for data analysis of LFP recordings. In brief, epileptiform spikes were automatically detected and analyzed for bursts, which were classified as low-, medium- and high-load bursts according to their spike load using a self-organizing map (SOM, Figure 3—figure supplement 1A–C). Bursts recorded in the 10 mM KA group covered the whole range of the SOM, but a larger fraction of these bursts matched nodes representing lower spike loads compared to the 15 and 20 mM KA group (Figure 3—figure supplement 1D).

Epileptiform activity occurred in both hippocampi of all KA mice (Figure 3A,B). The idHC of 10 mM KA mice showed a smaller percentage of high-load bursts compared to the 15 or 20 mM KA groups. In the cdHC, however, the incidence of high-load events was similarly low for the 10 and 20 mM KA groups (Figure 3C, Figure 3—source data 1). The mean ratio of time spent in high-load bursts (mean high-load burst ratio) and epileptic spike rate were significantly lower in the idHC 10 mM KA group compared to the 15 and 20 mM KA group (Figure 3D–G, Figure 3—source data 1). In the idHC the extent of GCD and hilar cell loss was positively correlated with the high-load burst ratio (Figure 3H, p<0.0001, Pearson’s r = 0.84; and Figure 3I, p<0.01, Pearson’s r = 0.64; both n = 15 animals).

Taken together, we developed the intrahippocampal KA mouse model further, creating varying degrees of disease severity on both the anatomical and electrophysiological level. Thus, this model provides a valuable framework for the following stimulation experiments.

### Application of different oLFS protocols during spontaneous epileptiform activity

**Figure 4.**
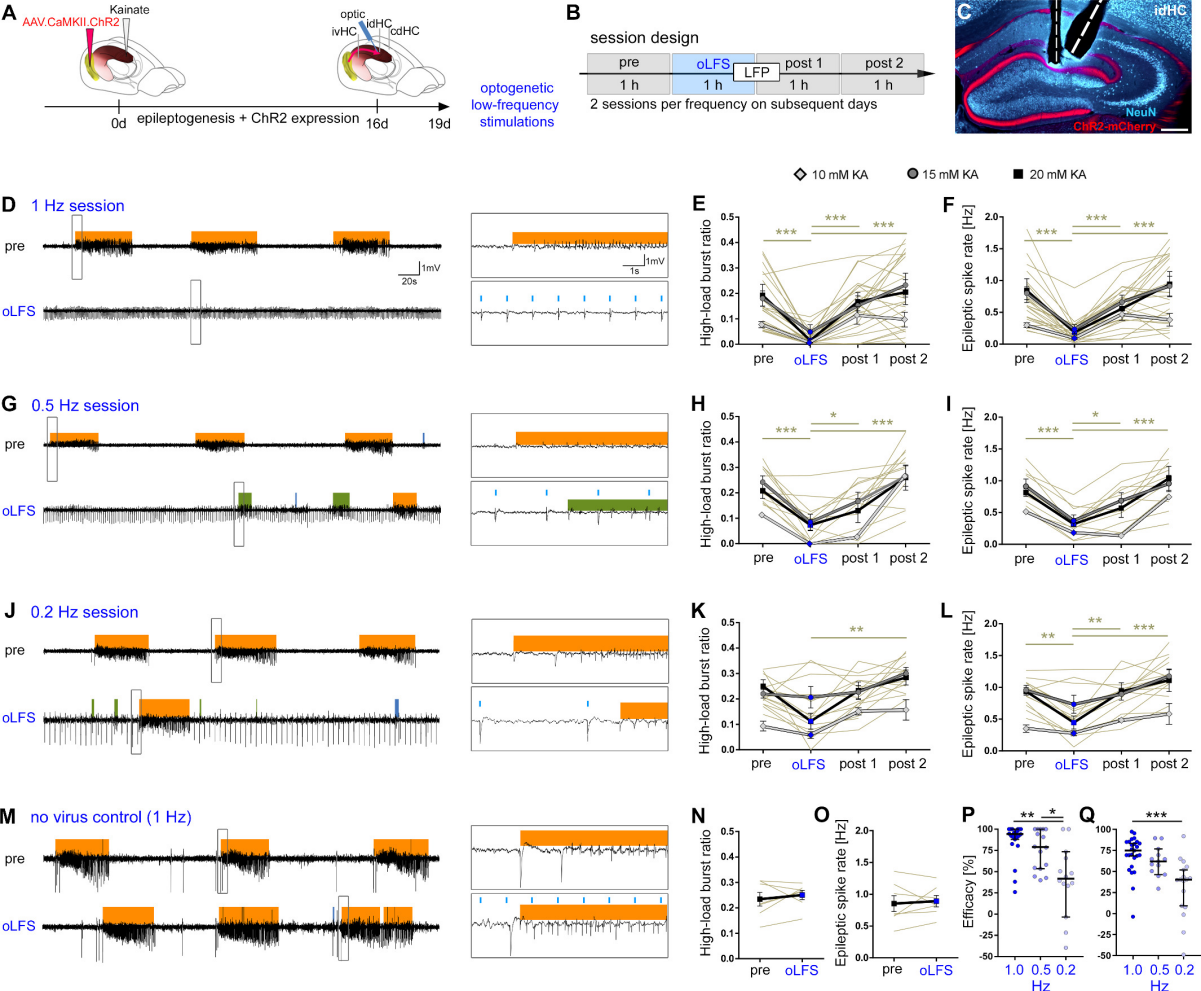
oLFS of entorhinal afferents interferes with spontaneous epileptiform activity in a frequency-dependent manner. (**A–C**) Experimental design. We targeted ChR2-mCherry expression (C, red) to excitatory neurons in the medial entorhinal cortex using viral vectors. ChR2-mCherry expression pattern for all mice included in the study are shown in Figure 4—figure supplement 2. The electrode positions are shown in Figure 4—figure supplement 3. (**B, C**) We locally stimulated entorhinal afferents in the sclerotic idHC for 1 hr per day, twice at each frequency applying only one frequency per session (1, 0.5, or 0.2 Hz). (**D, G, J, M**) Representative LFP traces (15 mM KA, idHC electrode) for the ‘pre’ and ‘oLFS’ sub-sessions (1, 0.5, 0.2 Hz and no-virus control, 1 Hz) are shown. Automatic detection of epileptiform activity is marked for low-load (blue), medium-load (green), and high-load bursts (orange). (**D**) Photostimulation at 1 Hz effectively decreases spontaneous epileptiform activity in the idHC. (**E, F**) Automatic quantification of epileptiform activity shows that oLFS reduces the high-load burst ratio as well as the epileptic spike rate in all animals independently of the KA concentration (10 mM: light gray; 15 mM: dark gray; 20 mM: black) followed by a return to pre-stimulation levels within 2 hr (‘post 1’ and ‘post 2’). (**G, J**) oLFS with (**H, I**) 0.5 Hz or (**K, L**) 0.2 Hz has a weaker antiepileptic effect during stimulation. Single sessions (olive-green) were used to calculate the one-way ANOVA; Tukey’s multiple comparison test (all KA concentrations pooled); *p<0.05, **p<0.01, and ***p<0.001. All mice were video recorded and the running behavior was analyzed during each session as shown in Figure 4—figure supplement 5 with the source data provided in Figure 4—figure supplement 5—source data 1. (**M–O**) 1 Hz stimulation does not have any effect on epileptiform activity in no-virus controls (20 mM KA). All values are given as mean ± SEM. Analysis of the cdHC is shown in Figure 4—figure supplement 1 with the source data provided in Figure 4—figure supplement 1—source data 1. We noticed that local oLFS in the idHC leads to a delayed cellular responses in the cdHC as shown in Figure 4—figure supplement 4. (**P, Q**) Comparison of the stimulation frequencies in terms of suppression efficacy using the high-load burst ratio and epileptic spike rate (1-(‘oLFS’/‘pre’)*100)(one-way ANOVA; Dunns’s multiple comparison test (all KA concentrations pooled), mean ±95% CI; *p<0.05, **p<0.01, ***p<0.001). Source data is provided in Figure 4—source data 1. Figure 4—source data 1.oLFS effect on ipsilateral epileptiform activity.Burst ratios and epileptic spike rates of each sub-session are listed for the three KA concentrations (10, 15, 20 mM) for each oLFS frequency (1, 0.5, 0.2 Hz) and no-virus controls (Ctr.). (**a, b**) The bust ratio and epileptic spike rate are reduced in all KA groups during oLFS but recover within the first hour of post-recording (post 1). This effect is also observed after 0.5 and 0.2 Hz oLFS but less pronounced. No change is observed in no-virus control animals. (**c, d**) Summary for all KA groups merged. (**e**) Median (±95% CI) of the suppression efficacy for the three applied frequencies. oLFS at 1 Hz seems most effective for the suppression of epileptiform activity. Values are given as mean ± SEM.

Since the sclerotic hippocampus is considered as the focus of epileptiform activity (Krook-Magnuson et al., 2015; Pallud et al., 2011), we targeted DGCs, the major surviving excitatory neurons, by photostimulation of entorhinal afferents. To this end, adult mice received KA into the hippocampus and a ChR2-carrying viral construct into the medial entorhinal cortex followed by LFP recordings and oLFS in the chronic epileptic phase (Figure 4A; Figure 4—figure supplement 1A). Prior to photostimulation, reference LFPs were recorded for 1 hr in ‘pre’ sessions to confirm the occurrence of spontaneous epileptiform activity in the idHC (Figure 4B) and cdHC (Figure 4—figure supplement 1B). Then, ChR2-expressing entorhinal fibers were stimulated in the sclerotic hippocampus (Figure 4C) with three frequencies (1, 0.5, and 0.2 Hz) on subsequent days. ChR2-mCherry expression and optic fiber position were verified histologically in all mice at the end of oLFS experiments (Figure 4—figure supplement 2, Figure 4—figure supplement 3).

**Figure 4—figure supplement 1.**
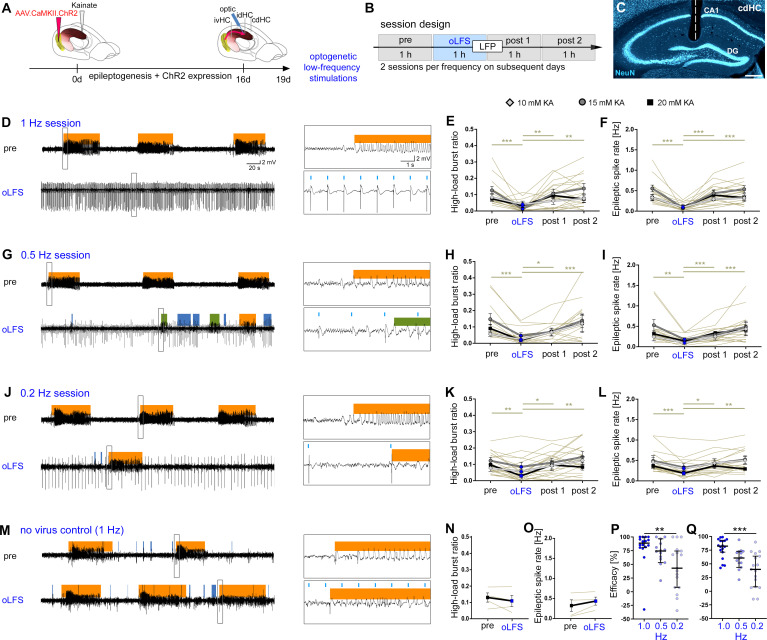
oLFS of entorhinal afferents in the idHC interferes with spontaneous epileptiform activity in the cdHC. (**A–C**) Experimental design. We optogenetically stimulated ChR2-mCherry expressing entorhinal afferents in the sclerotic idHC for 1 hr/day, twice at each frequency applying only one frequency per session (1, 0.5, or 0.2 Hz) and recorded LFP in both hippocampi. (**D, G, J, M**) Representative LFP traces (15 mM KA, cdHC electrode) for the ‘pre’ and ‘oLFS’ sub-sessions (1, 0.5, 0.2 Hz and no virus control, 1 Hz) are shown. Automatic detection of epileptiform activity is marked blue for low-, green for medium- and orange for high-load bursts. (**D**) Photostimulation at 1 Hz effectively decreases spontaneous epileptiform activity in the cdHC. (**E, F**) Automatic quantification of epileptiform activity shows that oLFS reduces the burst ratio as well as the epileptic spike rate in all animals independently of the KA concentration (10 mM: light gray; 15 mM: dark gray; 20 mM: black) followed by a return to pre-stimulation levels within 2 hr (‘post 1’ and ‘post 2’). (**G, J**) oLFS with 0.5 Hz or 0.2 Hz has a weaker antiepileptic effect (**H**, **I**, and **K**, **L**). Single sessions (olive-green) were used to calculate the one-way ANOVA; Tukey’s multiple comparison test (all KA concentrations merged); *p<0.05, **p<0.01, and ***p<0.001. (**M–O**) 1 Hz stimulation does not have any effect in no-virus controls (20 mM KA); Paired t-test. All values are given as mean ± SEM. (**P, Q**) Comparison of the stimulation frequencies in terms of suppression efficacy using the high-load burst ratio and epileptic spike rate (1-(‘oLFS’/‘pre’)*100) (one-way ANOVA; Dunns’s multiple comparison test (all KA concentrations pooled), mean ± 95% CI; **p<0.01, ***p<0.001). Source data is provided in Figure 4—figure supplement 1—source data 1. Figure 4—figure supplement 1—source data 1.oLFS effect on contralateral epileptiform activity.Burst ratios and epileptic spike rates of each sub-session are listed for the three KA concentrations (10, 15, 20 mM) for each oLFS frequency (1, 0.5, 0.2 Hz) and no-virus controls (Ctr.). (**a, b**) The bust ratio and epileptic spike rate are reduced in all KA groups during oLFS but recover within the first hour of post-recording (post 1). This effect is also observed after 0.5 and 0.2 Hz oLFS but less pronounced. No change is observed in no-virus control animals. (**c, d**) Summary for all KA groups merged. oLFS at 1 Hz seems most effective for the suppression of ictal and interictal activity in the contralateral dorsal hippocampus. (**e**) Median (±95% CI) of the suppression efficacy for the three applied frequencies. oLFS at 1 Hz seems most effective for the suppression of epileptiform activity. Values are given as mean ± SEM.

**Figure 4—figure supplement 2.**
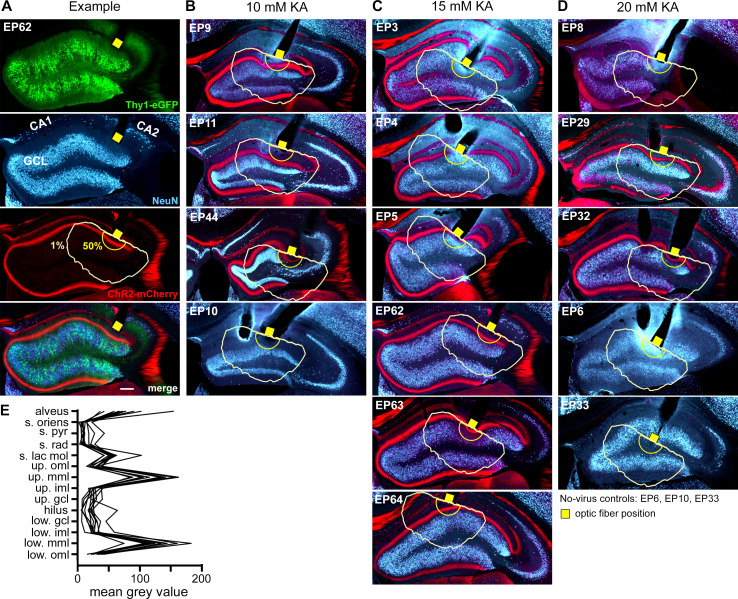
ChR2-mCherry expression pattern for all oLFS-stimulated mice. (**A–D**) After oLFS experiments, Thy1-eGFP mice were perfused and immunostained (NeuN, blue) for the identification of hippocampal sclerosis and ChR2-mCherry (red) expression. Representative pictures at the position of the optic fiber (yellow square) are shown for all mice. Following injection of the viral construct into the medial entorhinal cortex ChR2-mCherry is expressed in the medial perforant path terminating in the mid-molecular layer of the dentate gyrus (in the upper (up.) and lower (low.) blade). Additionally, the temporoammonic path is weakly labeled. Taking into account the restricted penetration depth of 473 nm light in brain tissue and an output of 150 mW/mm² at the tip of our optic fiber, we estimated that in our case the radius of light emission with sufficient power to activate ChR2 (yellow 1% line) does not exceed 500 µm. (**E**) Measurements of the mean gray value across the dorsoventral axis confirm the expression pattern of ChR2-mCherry in entorhinal afferents of the sclerotic hippocampus. s., stratum; lac., lacunosum; pyr., pyramidale; rad., radiatum; mol., moleculare; oml., outer molecular layer; mml., mid molecular layer; iml., inner molecular layer; gcl., granule cell layer. Scale bar 200 µm.

**Figure 4—figure supplement 3.**
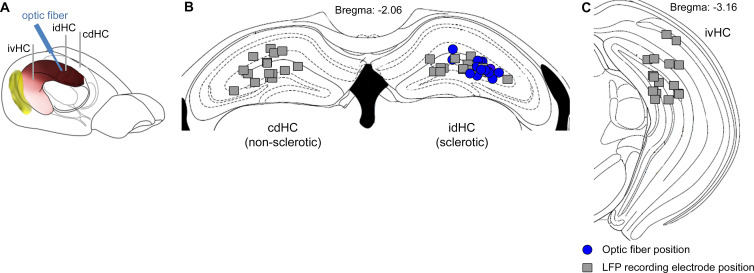
Positions of implanted electrodes and optic fibers for all animals included in the study. (**A**) Implantation scheme for oLFS. Electrode positions for all animals (n = 16) were histologically validated and located at the three different recording sites: cdHC, contralateral dorsal hippocampus; idHC, ipsilateral dorsal hippocampus and ivHC, ipsilateral ventral hippocampus. The optic fiber was placed in a 30° angle at the idHC site. (**B**) Positions of the optic fiber (blue circle) and LFP electrodes (gray square) in the dorsal region. (**C**) Ipsilateral ventral electrode positions.

**Figure 4—figure supplement 4.**
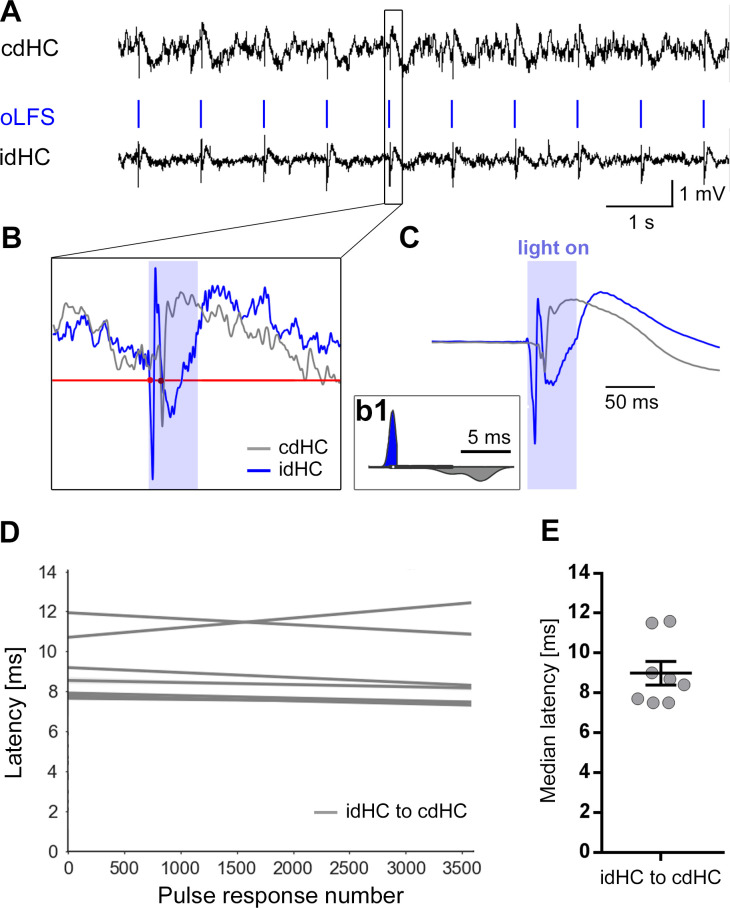
Local oLFS leads to delayed cellular responses in the cdHC. (**A**) Representative LFP traces of the two dorsal electrodes (idHC and cdHC) during 1 Hz oLFS. Local stimulation of DGCs via entorhinal afferents in the idHC evokes population spikes also in the cdHC. (**B**) One representative example of a population spike. They occur first in the idHC (blue) then in the cdHC (gray). For identification of the spike time, each voltage curve of a pulse response was first filtered and z-scored as described in the Materials and methods. Then, a manually chosen threshold (red line) was used to extract the population spike onset (red dots) for each pulse response in both hippocampi and was kept for all animals. (**b1**) Distribution of population spike onsets for 3600 responses (one representative example of a 1 Hz oLFS sub-session) to visualize the delay from idHC to cdHC. (**C**) Mean of all population spikes of one 1 Hz oLFS sub-session. (**D**) Linear regression shows stable latencies from idHC to cdHC (n = 8 sessions from seven animals) over 1-hr oLFS. Individual latency data points are not shown for visualization. (**E**) Median latencies for each analyzed oLFS sub-session. Population spikes occur with a latency of 8–12 ms (mean ± SEM: 9 ± 0.6 ms) in the cdHC relative to the population spike onsets in the idHC. Calculations were performed in python 2.7 provided in Figure 4—figure supplement 4—source code 1. Figure 4—figure supplement 4—source code 1.1 Hz oLFS response: idHC to cdHC delay calculation.LFP data (smr files for idHC and cdHC channels) were imported, z-scored and filtered (4th order low pass Chebyshev filter type I with a cut-off frequency at 300 Hz). LFP-snippets of a defined time interval (−0.1 s, +0.2 s relative to stimulus onset) were used to identify the crossing of a previously identified threshold (this threshold was kept constant for all animals). The delay of the stimulation pulse response from idHC to cdHC was then calculated for each LFP-snippet.

**Figure 4—figure supplement 5.**
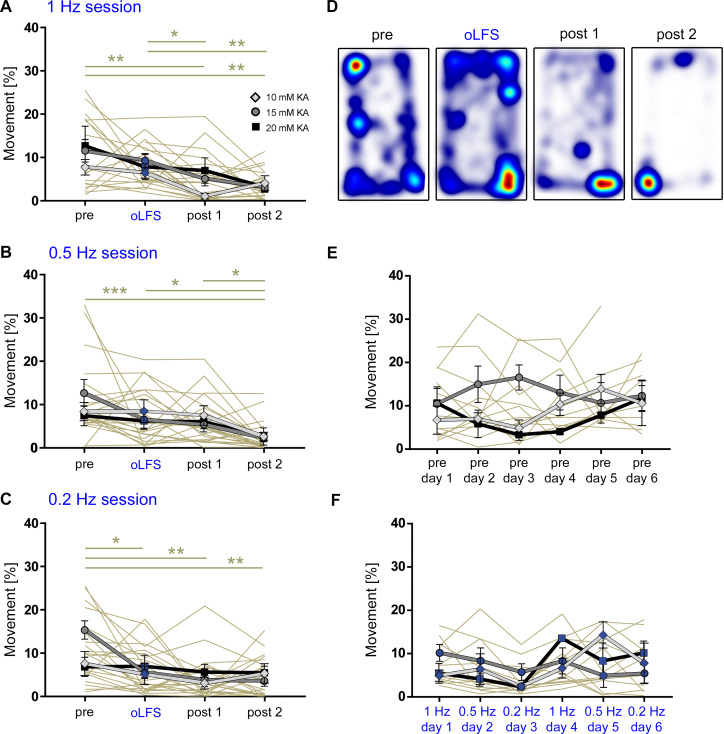
Movement analysis of chronically epileptic mice during LFP recordings. Freely moving animals were video tracked. Running phases were automatically detected and quantified as time spent running (>4 cm/s). (**A–C**) Analysis of all sub-session reveals that the running behavior of all KA-treated mice is comparable and it declines gradually during the recording time of 4 hr. (**D**) Representative heat map of a 1 Hz session with the corresponding ‘pre’ and ‘post’ recordings. Warm colors indicate places of longer stays during each sub-session. (**E, F**) No statistically significant difference could be detected for the running behavior during oLFS experiments (6 days in a row) in the ‘pre’ or the ‘oLFS’ sessions. Hence, all experiments were performed under comparable conditions. Individual values are presented in Figure 4—figure supplement 5—source data 1. Single sessions (olive-green) are used to calculate the one-way ANOVA; Tukey’s multiple comparison test (all KA concentrations merged); *p<0.05, **p<0.01 and ***p<0.001. Values are given as mean ± SEM. Figure 4—figure supplement 5—source data 1.oLFS effect on running behavior over time.(**a**) Time spent running (>4 cm/s) of each sub-session is listed for the three KA concentrations (10, 15, 20 mM) for each oLFS frequency (1, 0.5, 0.2 Hz). The running behavior between KA groups is not significantly different. (**b**) During oLFS sessions, the movement is not impaired. The decrease in the movement over the 4 hr of LFP recording is most likely due to adaptation effects to the environment. Values are given as mean ± SEM.

One hour of optogenetic stimulation with pulsed light at 1 Hz or 0.5 Hz significantly decreased the high-load burst ratio and the epileptic spike rate, followed by a return to pre-stimulation levels within 2 hr independent of the KA concentration (Figure 4D–I, Figure 4—source data 1). Photostimulation at 0.2 Hz had no significantly suppressive effect on the high-load burst ratio, but on the epileptic spike rate (Figure 4J–L; Figure 4—source data 1). 1 Hz oLFS was significantly more effective than 0.2 Hz in suppressing high-load bursts and epileptic spikes, whereas we found no significant difference between 1 Hz and 0.5 Hz (Figure 4P,Q; Figure 4—source data 1). Looking at individual sessions in more detail, 1 Hz oLFS had a higher percentage of sessions with a suppression efficacy above 75% than 0.5 Hz and 0.2 Hz regarding high-load burst ratio and epileptic spike rate (1 Hz: 86.36% and 48.00%, 0.5 Hz: 56.25% and 23.08%, 0.2 Hz: 14.29% and 5.88%). As expected, oLFS (1 Hz) did not influence epileptiform activity in no-virus control mice (Figure 4M–O, Figure 4—source data 1).

To clarify whether the suppression of high-load bursts and epileptic spikes due to oLFS was locally restricted to the stimulation site (idHC), we analyzed LFPs contralaterally (cdHC), outside of the epileptic focus (Figure 4—figure supplement 1A–C). Interestingly, high-load burst ratio and epileptic spike rate were also suppressed in the cdHC (Figure 4—figure supplement 1D–O, Figure 4—figure supplement 1—source data 1). 1 Hz oLFS was significantly more effective than 0.2 Hz in reducing high-load bursts and epileptic spikes, whereas there was no significant difference between 1 Hz and 0.5 Hz (Figure 4—figure supplement 1P,Q; Figure 4—figure supplement 1—source data 1). Looking at individual sessions in detail, 1 Hz oLFS had a higher percentage of sessions with a suppression efficacy above 75% than 0.5 Hz and 0.2 Hz regarding high-load burst ratio and epileptic spike rate (1 Hz: 87.50% and 66.67%, 0.5 Hz: 50.00% and 7.14%, 0.2 Hz: 23.53% and 5.88%).

Next, we tested whether the neuronal responses to oLFS were confined to the stimulated area by analyzing the spatial and temporal occurrence of evoked responses in the idHC and cdHC. In all animals, pulsed light delivery to the idHC did not only trigger local but also delayed responses in the cdHC (Figure 4—figure supplement 4A–C). These latencies remained stable over the stimulation period of 1 hr (Figure 4—figure supplement 4D) ranging from 8 to 12 ms (Figure 4—figure supplement 4E; 8.99 ± 0.59 ms, n = 8 sessions), suggesting that photostimulation of entorhinal afferents may lead to repeated action potential generation in a subset of DGCs and subsequent propagation within the hippocampal network.

In parallel to LFP recordings and optogenetic stimulation, we assessed the animals’ motor behavior in an open-field environment. Mice frequently groomed and explored their environment during oLFS. Video tracking revealed that independent of the KA concentration and stimulation frequency, mice did not change their running behavior during oLFS compared to the ‘pre’ recording. The percentage of running time gradually declined during the recording time of 4 hr (Figure 4—figure supplement 5A–D, Figure 4—figure supplement 5—source data 1). The percentage of running time in all ‘pre’ and ‘oLFS’ sessions over the 6 days of stimulation was stable (Figure 4—figure supplement 5E,F), indicating that hippocampal oLFS did not impair open-field running behavior of chronically epileptic mice.

So far, our findings demonstrated that 1 Hz oLFS of entorhinal afferents was highly effective in the suppression of spontaneous epileptiform activity within as well as outside of the epileptic focus. Moreover, this effect was independent of the degree of hippocampal sclerosis and seizure burden. Therefore, we pooled all KA groups in the following experiments.

### Effects of oLFS and eLFS on induced behavioral seizures

In the intrahippocampal KA model, spontaneous epileptiform activity is mainly subclinical and rarely generalizes into behavioral seizures (Häussler et al., 2012; Janz et al., 2018; Klein et al., 2015). To assess the impact of oLFS on generalized seizures, we induced these seizures by 10 Hz photostimulation as described previously (Janz et al., 2018; Osawa et al., 2013). In addition to the evoked potentials during stimulation, high-amplitude epileptic spikes emerged in the LFP which gradually became rhythmic and dominant before progressing into full-blown behavioral seizures (Figure 5—figure supplement 1A). These evoked seizures displayed electrographic features highly similar to those of spontaneous generalized seizures (Figure 5—figure supplement 1B) and were accompanied by the same stereotypic myoclonic movements (e.g. rearing, falling, and convulsion).

**Figure 5.**
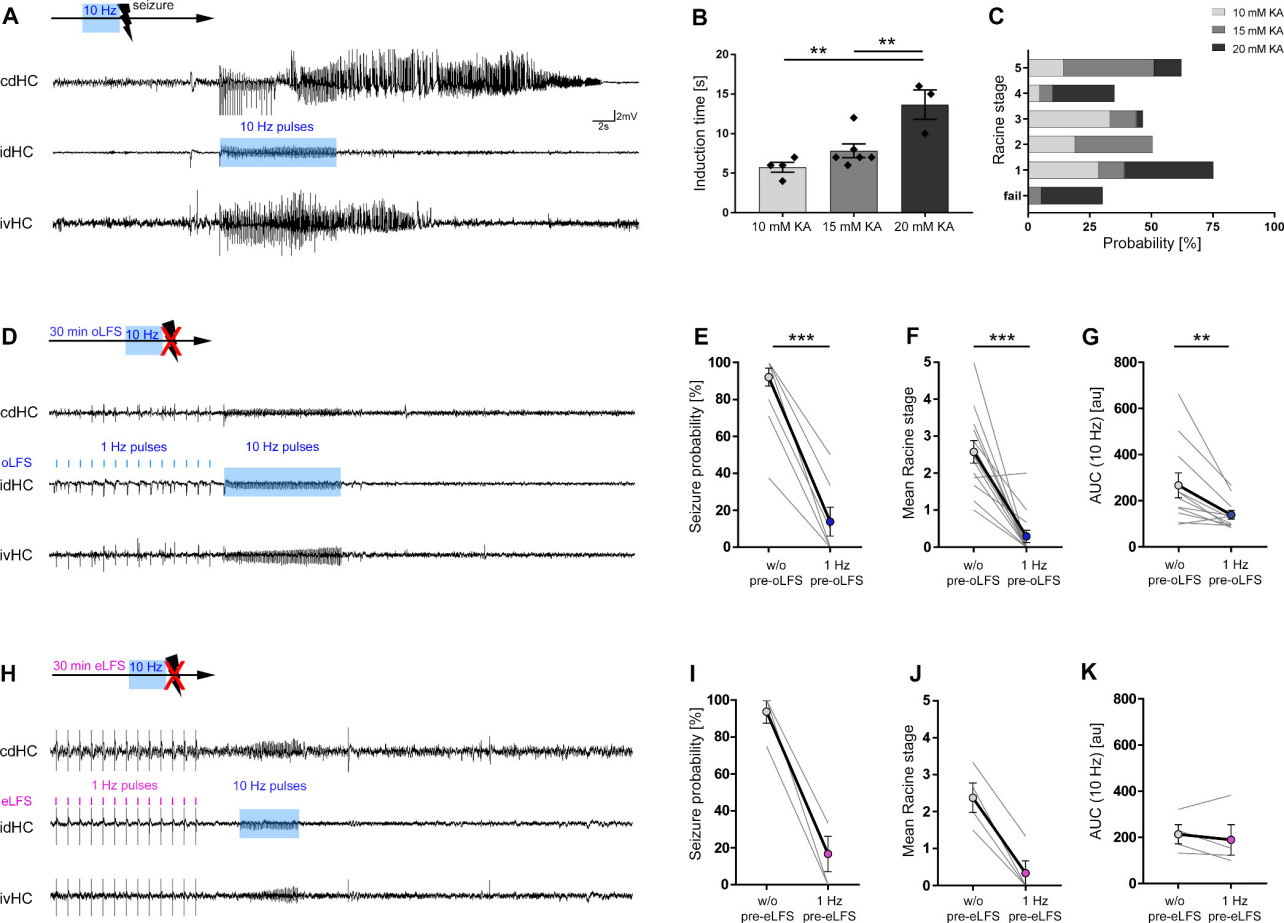
Preconditioning with LFS prevents optically evoked seizure generalization. (**A, D, H**) Representative LFP traces at three recording sites (cdHC, idHC, and ipsilateral ventral hippocampus (ivHC)). A schematic of the respective stimulation procedure is shown above each cdHC trace. (**A, B**) Local 10 Hz photostimulation of entorhinal afferents reliably induces generalized seizures in all KA groups. Evoked, generalized seizures displayed electrographic features highly similar to spontaneous generalized seizures as shown in Figure 5—figure supplement 1. The time needed to induce a generalized seizure (induction time) is longer with increasing KA concentration. One-way ANOVA; Tukey’s multiple comparison test; **p<0.01. (**C**) Mice exhibit behavioral symptoms equivalent to RS stage 1–5, independently of the KA concentration. (**D, E**) 1 Hz oLFS as well as (**H, I**) eLFS for 30 min before the pro-convulsive stimulus significantly decreases the seizure probability in all animals. Wilcoxon rank test, matched-pairs; ***p<0.001 (oLFS, n = 13; eLFS, n = 4 animals). Preconditioning with 0.5 Hz was also able to interfere with the generation of evoked generalized seizures as shown in Figure 5—figure supplement 2. (**F, J**) Trials in which seizure generalization is not prevented completely, the ensuing seizure is associated with a milder behavioral phenotype (RS). Wilcoxon rank test, matched-pairs; ***p<0.001 (oLFS, n = 13; eLFS, n = 4 animals). (**G, K**) Cellular response to 10 Hz stimulation quantified as mean AUC. (**G**) The response is reduced after 1 Hz oLFS stimulation in sessions in which seizures have been successfully suppressed. (**K**) No significant reduction is visible for AUC values after eLFS. Paired t-test; **p<0.01 (oLFS, n = 13; eLFS, n = 4 animals, respectively). All values are given as mean ± SEM. AUC calculation was performed in python 2.7 provided in Figure 5—source code 1. Figure 5—source code 1.AUC calculation of 10 Hz oLFS evoked responses.LFP data (idHC) were imported to python 2.7 and filtered (4th order low-pass Chebyshev filter type I with a cut-off frequency at 300 Hz) to smoothen the signal. We calculated the AUC of evoked responses (integral of LFP traces in a defined time interval) during pro-convulsive optical stimulation (10 Hz). The time interval for AUC calculation was set relative to stimulus onset from −0.02 s to +0.06 s.

We determined the minimum stimulus duration sufficient to reliably trigger a generalized seizure for each animal (Figure 5A, as described in the Materials and methods under Optogenetic stimulation). Interestingly, in mice with lower KA concentrations, generalized seizures were induced much faster, suggesting a higher susceptibility for seizure generalization (Figure 5B, 10 mM: 5.75 ± 0.63 s; 15 mM: 7.83 ± 0.87 s; 20 mM: 13.67 ± 1.86 s, 10 and 15 mM vs. 20 mM p<0.01; n = 3; 6; 4 animals). With ongoing seizure activity, mice exhibited behavioral symptoms equivalent to Racine stages (RS) 1 to 5 (Racine, 1972) independent of the stimulation duration (Figure 5C, n = 3; 6; 4 animals).

**Figure 5—figure supplement 1.**
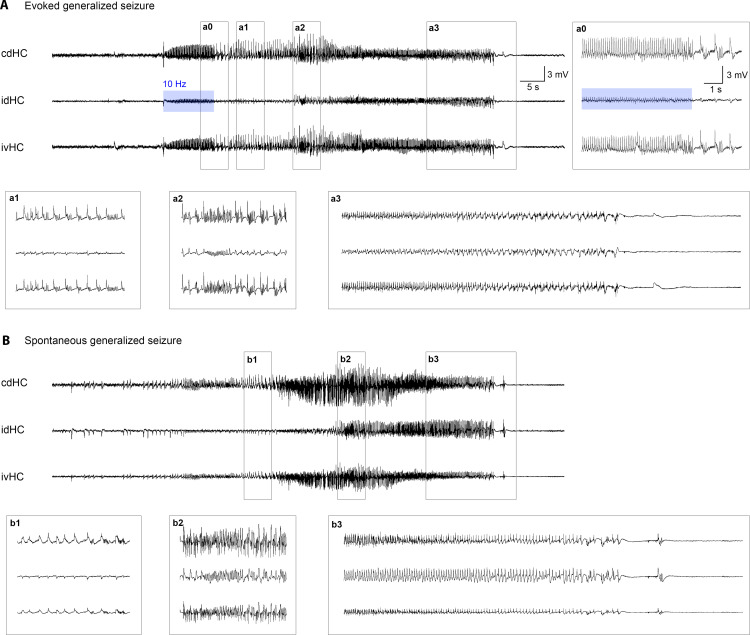
Comparison of spontaneous and evoked generalized seizures. (**A, B**) Representative LFP traces of an optogenetically evoked and a spontaneous generalized seizure from the same animal. (**a0**) High-amplitude epileptic spikes emerge in addition to the evoked potentials during stimulation and rhythmic activity persists after 10 Hz stimulation has been stopped. Both seizures consist of the same building blocks [(**a1, b1**) spike-and-wave events; (**a2, b2**) fast discharges; (**a3, b3**) increasing inter-spike-intervals and subsequent termination] with similar dynamics. This is consistent for all mice (n = 5) in which both spontaneous and evoked generalized seizures were detected.

**Figure 5—figure supplement 2.**
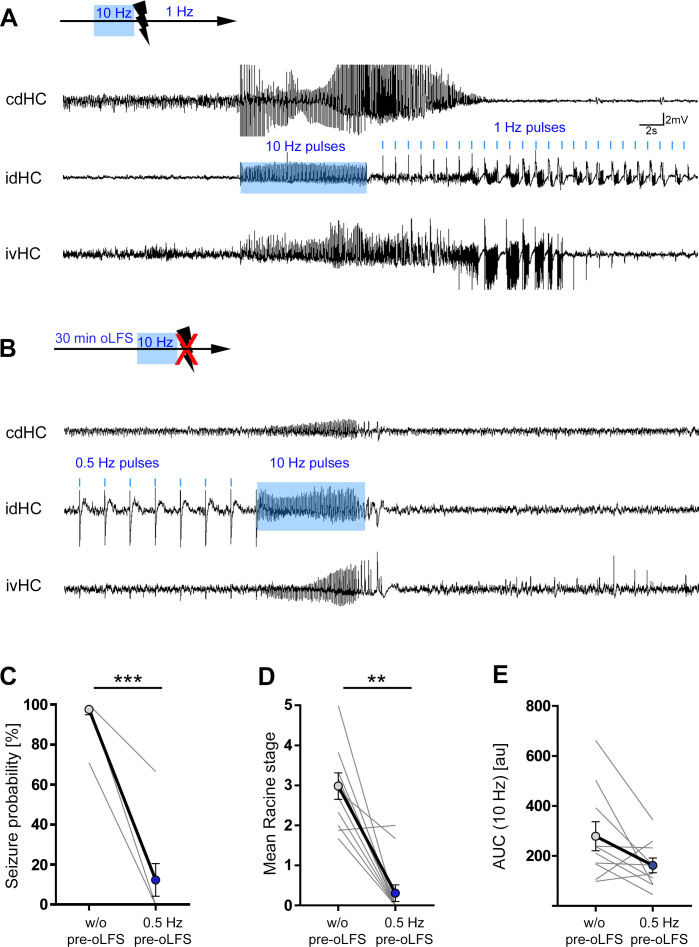
Preconditioning with 0.5 Hz prevents evoked generalized seizures. (**A**) Representative LFP trace of an optogenetically evoked generalized seizure. Seizure generalization cannot be prevented by oLFS (1 Hz) applied directly after seizure induction. (**B**) oLFS at 0.5 Hz for 30 min prior to the pro-convulsive stimulus effectively prevents seizure generalization. (**C**) 0.5 Hz oLFS significantly decrease the seizure probability in all animals. Wilcoxon rank test, matched-pairs; ***p<0.001 (n = 10 animals). (**D**) Trials in which seizure generalization is not prevented completely, the ensuing seizure is associated with a milder behavioral phenotype (RS). Wilcoxon rank test, matched-pairs; **p<0.01, (n = 10 animals). (**E**) The cellular response to the 10 Hz stimulation (mean AUC) is reduced after 0.5 Hz oLFS stimulation in sessions in which seizures have been successfully suppressed. Paired t-test; *p<0.05 (n = 10 animals).

Next, we probed whether oLFS can interfere with generalized seizures. When 1 Hz oLFS was started directly after the pro-convulsive 10 Hz stimulus, ongoing seizures were not interrupted (Figure 5—figure supplement 2A). In contrast, pre-conditioning with 1 Hz oLFS for 30 min prior to the pro-convulsive stimulus was highly effective in lowering the probability for seizure generalization (Figure 5D,E, without (w/o) pre-oLFS: 91.42 ± 5.18%; with 1 Hz pre-oLFS: 14.87 ± 8.33%, p<0.001, n = 13 animals). 0.5 Hz oLFS was also effective in preventing evoked generalized seizures (Figure 5—figure supplement 2B,C, w/o pre-oLFS: 97.73 ± 2.27%; with 0.5 Hz pre-oLFS: 12.12 ± 8.13%, p<0.05; n = 11 animals). In trials with incomplete seizure suppression, the ensuing seizures were associated with a milder behavioral phenotype (Figure 5F, w/o oLFS: RS 2.58 ± 0.31; with 1 Hz oLFS: RS 0.29 ± 0.17, n = 13 animals; Figure 5—figure supplement 2D, w/o oLFS: RS 2.88 ± 0.32; with 0.5 Hz oLFS: RS 0.37 ± 0.25, p<0.01; n = 10 animals).

Since LFS and seizure induction were both driven by photostimulation, we performed additional experiments combining optogenetic 10 Hz stimulation with electrical 1 Hz preconditioning in the same animal. To this end, mice received intrahippocampal KA (15 mM) and the ChR2-carrying viral construct into the medial entorhinal cortex followed by a side-by-side implantation of an optic fiber and a stimulation electrode into the dentate gyrus. In addition, all animals were implanted with LFP recording electrodes as described above (Figure 1B).

We determined the minimum optical stimulus duration sufficient to reliably trigger a generalized seizure. Then, we probed the seizure-suppressive action of 1 Hz eLFS prior to optogenetic 10 Hz seizure induction (Figure 5H). Thirty minutes of 1 Hz eLFS was as effective as oLFS in reducing the probability of generalized seizures (Figure 5I, w/o pre-eLFS: 93.75 ± 6.25%; with 1 Hz pre-eLFS: 16.67 ± 9.62%) and the associated behavior (Figure 5J, w/o eLFS: RS 2.38 ± 0.40; with 1 Hz eLFS: RS 0.33 ± 0.33, n = 4 animals).

In conclusion, preconditioning by optogenetic and electrical 1 Hz LFS was highly effective in preventing evoked generalized seizures.

### Cellular responses to oLFS

In order to investigate the underlying mechanisms of the anti-epileptic effects of oLFS, we quantified the cellular responses in the sclerotic hippocampus. For this, we calculated the area under the curve (AUC) of each evoked response over the 1-hr stimulation period. We only selected sessions with high stimulation efficacy (within the 95% confidence interval (CI), compare Figure 4P).

**Figure 6.**
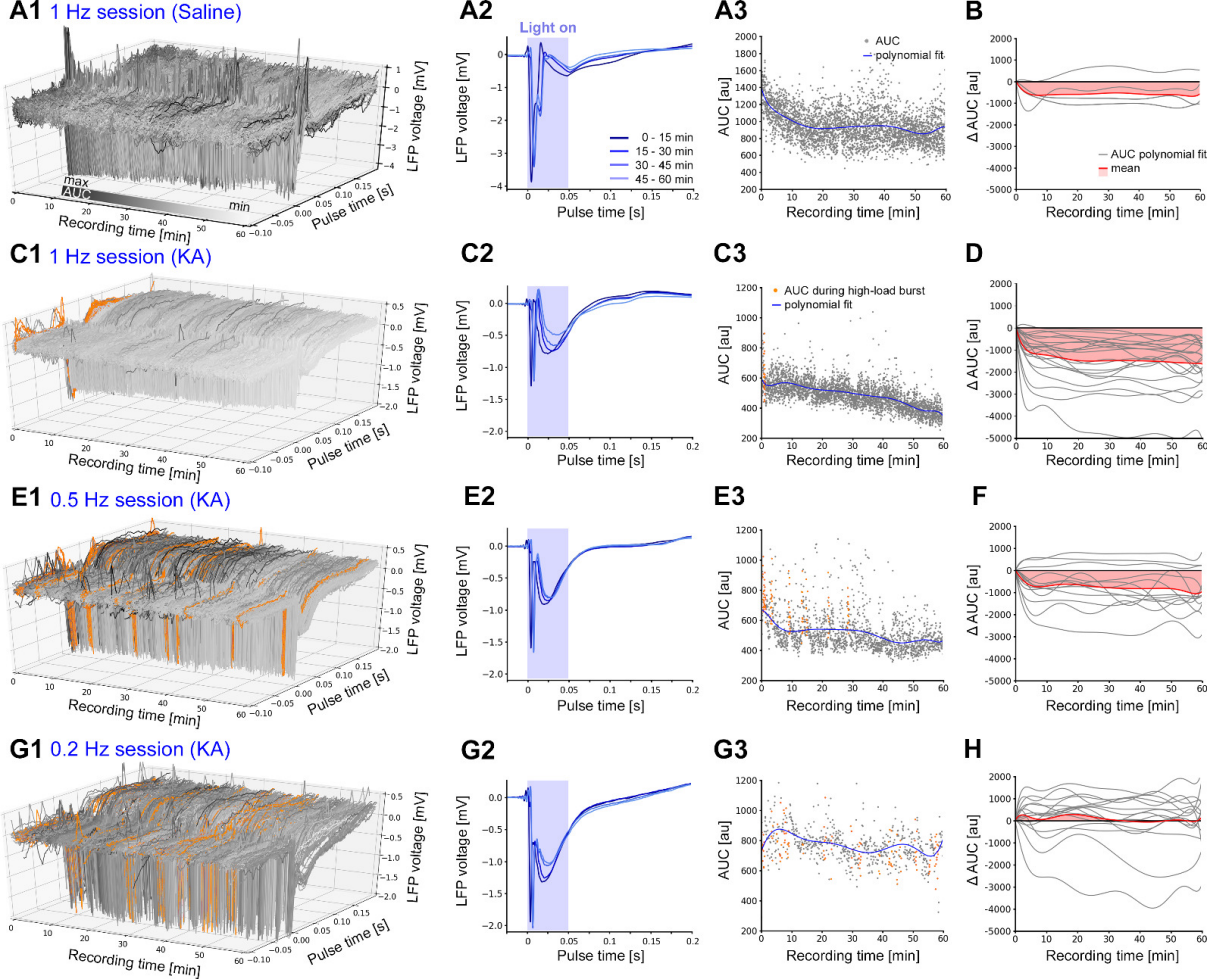
Evoked cellular responses decrease over time during continuous oLFS. (**A, C, E, G**) Representative examples of evoked responses in the dentate gyrus of idHC following local photostimulation of entorhinal afferents for (**A**) non-epileptic control (1 Hz) and (**C, E, G**) a chronically epileptic mouse (1, 0.5, and 0.2 Hz). (**A2, C2, E2, G2**) Mean evoked responses (50 ms–long light pulse) across 15 min time windows. (**A3, C3, E3, G3**) For each evoked response, AUCs are calculated during a [−0.1, +0.2 s] interval relative to the onset of each light pulse. AUC values that are within high-load bursts are marked in orange and are excluded for the calculation of the polynomial fit (blue line). (**B, D, F, H**) Polynomial fits of AUC normalized to the first AUC value (delta AUC) for all stimulation sessions (gray) and mean changes (red). Calculations were performed in python 2.7 provided in Figure 6—source code 1. Figure 6—source code 1.AUC calculation of 1 Hz oLFS evoked responses.LFP data (idHC) were imported to python 2.7 and filtered (4th order low-pass Chebyshev filter type I with a cut-off frequency at 300 Hz) to smoothen the signal. We calculated the AUC of evoked responses (integral of LFP traces in a defined time interval = LFP snippet) during oLFS (1, 0.5, 0.2 Hz). The time interval for AUC calculation was set relative to stimulus onset from −0.1 s to +0.2 s. For responses co-occurring with high-load bursts, the AUC was not determined. Using a representative example, we plotted a mean stimulation response across 15 min time windows and individual AUC values for 1-hr oLFS. Polynomial fits of AUC values normalized to the first AUC value (delta AUC) for all stimulation sessions were then calculated and plotted.

Photostimulation (1 Hz) evoked stable response waveforms (Figure 6) which decreased slightly in amplitude over time in non-epileptic (saline-injected) mice (Figure 6A1-3, B). In chronically epileptic mice, the cellular responses declined strongly and rapidly within the first 10 min (Figure 6C1-3, D). Lower frequencies (0.5 and 0.2 Hz) altered the cellular response much less (Figure 6E–H) as evident from AUC analysis. Similarly, AUCs of evoked responses of the pro-convulsive 10 Hz pulse-train were reduced by about 40% after pre-conditioning when compared to the responses without preconditioning (Figure 5G, AUC w/o oLFS: 266.9 ± 53.89; with 1 Hz oLFS: 139.2 ± 19.08, p<0.01, n = 11 animals; Figure 5K, w/o eLFS: 213.4 ± 41.16; with 1 Hz eLFS: 189.2 ± 65.33, n = 4 animals; Figure 5—figure supplement 2E, AUC w/o oLFS: 278.9 ± 58.09; with 0.5 Hz oLFS: 162.1 ± 29.11, n = 10 animals).

**Figure 7.**
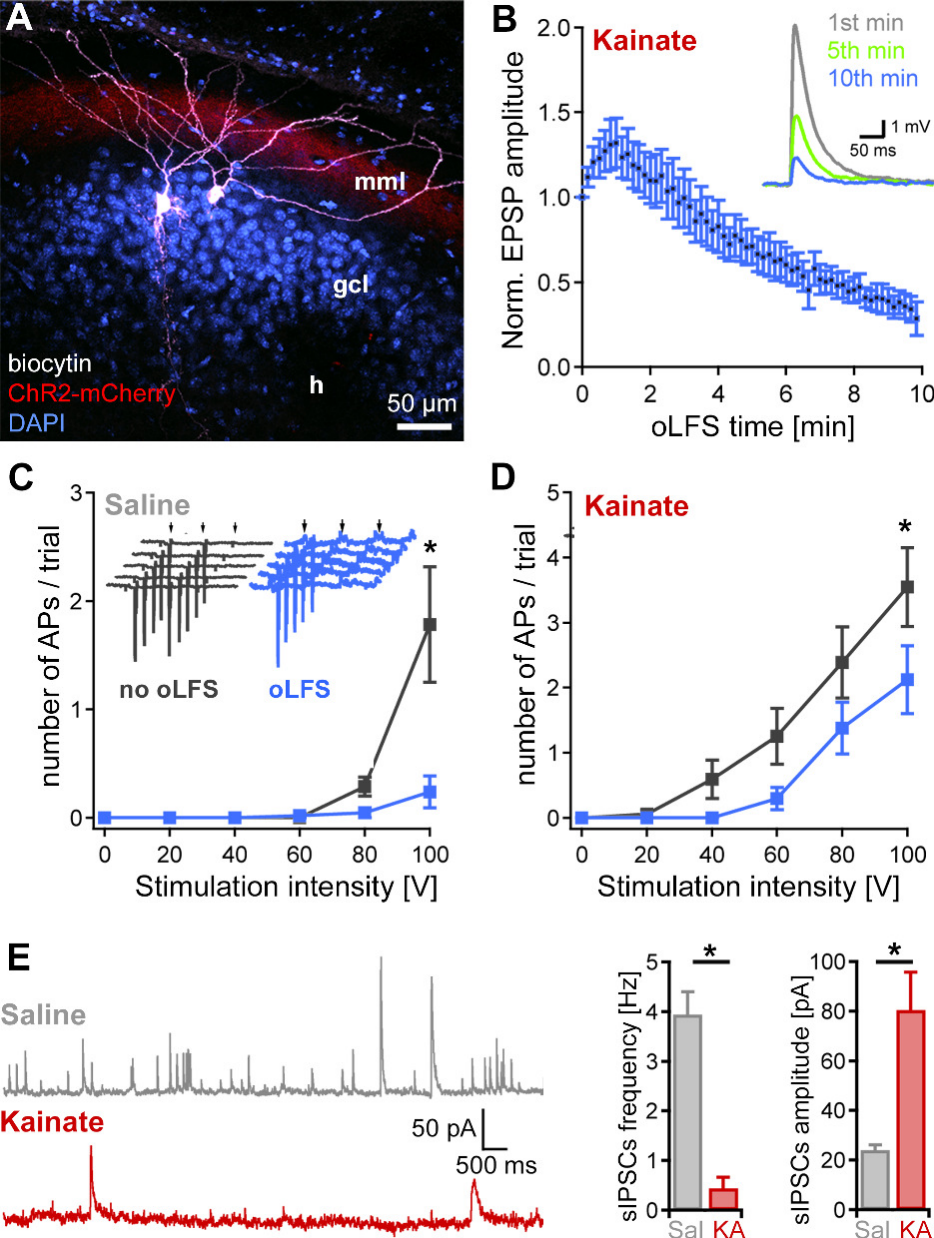
Decrease of single-cell EPSPs and discharge probability after 10 min oLFS. (**A**) Representative confocal projection of a dentate gyrus slice from a KA- (15 mM) and AAV-treated mouse 28 days after SE. The two biocytin-filled DGCs (white) were recorded in this section. ChR2-expressing entorhinal fibers are visible in the middle molecular layer (red, mml). Cell bodies were stained with DAPI (blue). h, hilus; scale bar 50 µm. (**B**) Pulsed blue light delivery reliably induces EPSPs, which decline strongly during a 10-min stimulation protocol (50 ms pulses at 1 Hz). (**C, D**) Extracellular electrical stimulation (trial = 5 pulses, 50 Hz, arrows) of entorhinal fibers induces action potentials (APs) measured in DGCs (inset, gray traces, recorded in loose-patch). Photostimulation at 1 Hz over 10 min (inset, blue traces) significantly reduces the discharge probability of DGCs in epileptic KA (n = 19 cells) and saline (n = 11 cells) control slices. ANOVA on ranks with Dunn’s Bonferroni post-hoc correction; *p<0.05. Values are given as mean ± SEM. (**E**) Spontaneous IPSCs in DGCs of saline (n = 6 cells) and KA (n = 7 cells) slices were analyzed for frequency and amplitude. The number of sIPSCs is strongly reduced in ‘epileptic’ DGCs compared to control but those that occur have a larger amplitude. Mann-Whitney Rank Sum Test; *p<0.05 both.

To investigate whether oLFS decreases the excitability of DGCs at the single-cell level, we studied their intrinsic properties and the synaptic strength of entorhinal inputs in acute slices obtained from chronically epileptic mice. We performed whole-cell recordings of DGCs located in the outer region of the dispersed GCL in the presence of GABA_A_ and GABA_B_ receptor blockers as described in the Methods. Cells were filled with biocytin during recordings for subsequent morphological identification (Figure 7A, n = 14 animals). Photostimulation of afferent entorhinal fibers robustly induced depolarization of DGCs (5.1 ± 1.0 mV, n = 14 cells) and was occasionally sufficient to induce action potentials (−70 mV holding potential, n = 4 cells). Since cellular responses *in vivo* declined strongly and rapidly within the first 10 min, we applied 10 min oLFS at 1 Hz to acute slice preparations. During oLFS, evoked synaptic responses were strongly depressed (Figure 7B, reduction of excitatory postsynaptic potential (EPSP) amplitude to 28.5 ± 9.9% of the original response, n = 14 cells). Next, we evaluated the effect of oLFS on discharge probability upon electrical stimulation (trial = 5 pulses at 50 Hz) of entorhinal fibers. To maintain stable intracellular conditions in DGCs, we used cell-attached recordings. In non-sclerotic control slices (saline mice), oLFS for 10 min reduced the discharge probability of DGCs (Figure 7C, number of action potentials generated per trial at 100 V stimulation, no-oLFS: 1.8 ± 0.5 vs. after oLFS: 0.24 ± 0.15, p<0.05, n = 11 cells). Although DGCs were overall more excitable in KA mice, as reported previously in this model (Janz et al., 2017a), the number of action potentials in response to electrical stimulation was significantly reduced upon 10 min oLFS (Figure 7D, number of action potentials generated per trial at 100 V stimulation, no-oLFS: 3.5 ± 0.6 vs. after oLFS: 2.1 ± 0.5, p<0.05, n = 19 cells).

In contrast, intrinsic properties of DGCs in KA mice were not altered by the applied stimulation protocol (control: resting membrane potential (V_m_) = −73.4 ± 1.1 mV; C_m_ = 53.5 ± 3.7 pF; R_m_ = 298.7 ± 27.4 MΩ; Rheobase = 150.8 ± 14.1 pA; n = 27 cells; after oLFS: V_m_ = −74.8 ± 1.3 mV; C_m_ = 56.4 ± 7.5 pF; R_m_ = 313.9 ± 36.8 MΩ; Rheobase = 153.3 ± 26.4 pA; n = 14 cells). The reduced excitability of DGCs during oLFS may be explained by a reduced glutamate release from entorhinal projections. Additionally, we measured spontaneous inhibitory postsynaptic currents (sIPSCs) of DGCs in slices of control and epileptic mice and confirmed that KA-treated mice have strongly reduced inhibition in the sclerotic hippocampus which is most likely due to the loss of inhibitory interneurons (Figure 7E, sIPSCs frequency: saline 3.9 ± 0.5 Hz vs. KA 0.4 ± 0.2 Hz, p<0.05, n = 5 cells; sIPSCs amplitude: saline 24.0 ± 2.1 pA vs. KA 80.4 ± 15.2 pA, p<0.05, n = 4 cells, all at +20 mV).

### Effects of repeated hippocampal eLFS on epileptiform activity

**Figure 8.**
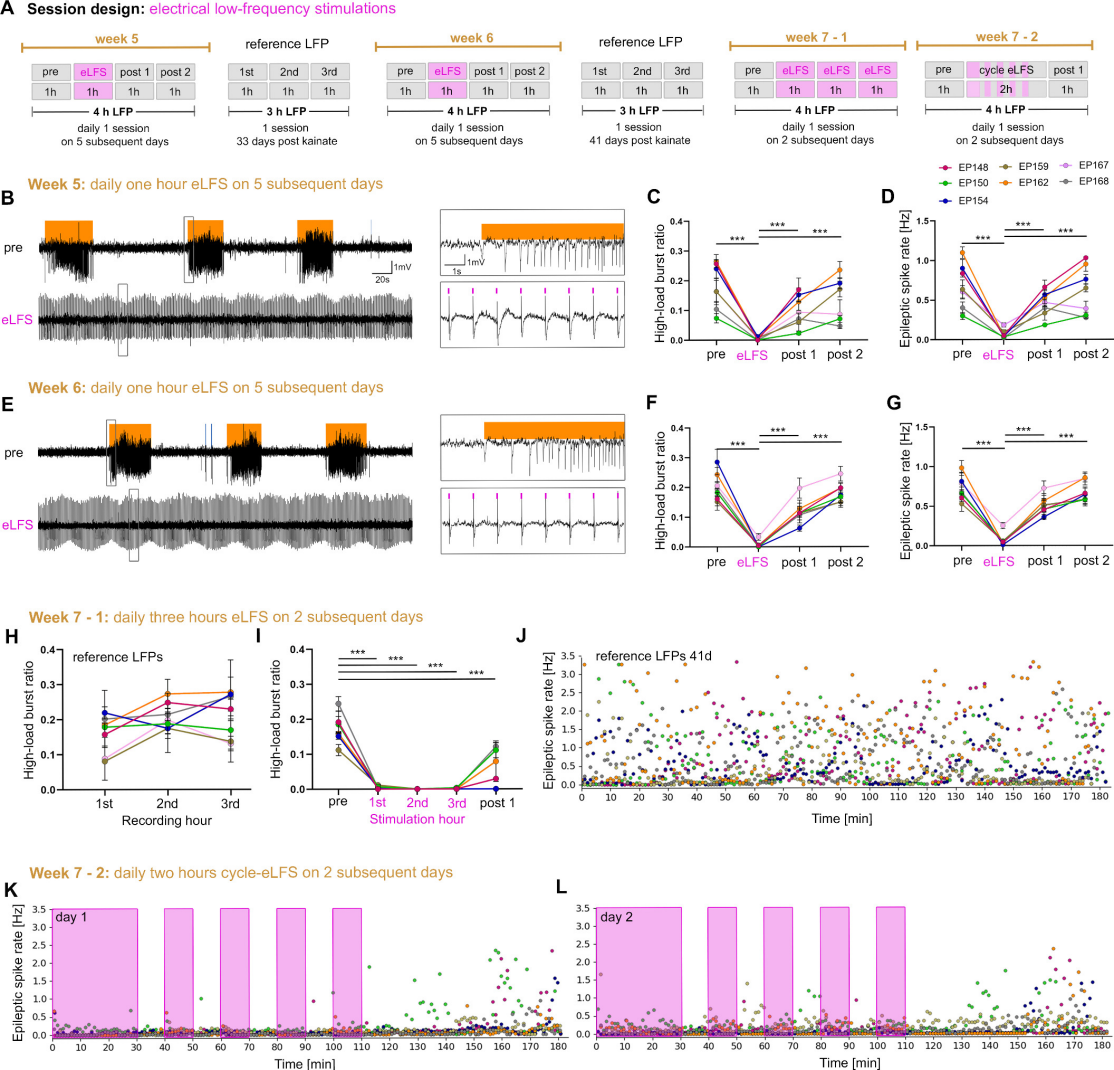
Stable suppression of epileptiform activity through eLFS in the sclerotic hippocampus. (**A**) Session design. We locally stimulated the dentate gyrus in the sclerotic idHC for 1 hr/day (1 Hz) over 2 weeks. In the third week, we stimulated animals 3 hr continuously and 2 hr in a 10 min ‘on-off’ manner on 4 successive days. (**B, E**) Representative LFP traces (15 mM KA, idHC electrode) for the ‘pre’ and ‘eLFS’ sub-sessions (weeks 5 and 6) are shown. Automatically detected high-load bursts are indicated by an orange bar. (**C, D, F, G**) Quantification of epileptiform activity shows that eLFS nearly extinguishes epileptiform activity in all animals followed by a return to pre-stimulation levels within 2 hr (‘post 1’ and ‘post 2’). Single sessions were used to calculate the RM one-way ANOVA; Tukey’s multiple comparison test ***p<0.001. Animals with a misplaced stimulation electrode did not show this remarkable suppression of epileptiform activity as shown in Figure 8—figure supplement 1. The correct positions of electrodes were confirmed by histology as shown in Figure 8—figure supplement 2. (**H**) Animals have a stable high-load burst ratio over 3 hr of reference recordings. (**I**) 3 hr continuous eLFS effectively reduces the high-load burst ratio over the whole stimulation period, reoccurring in a lower manner within 1 hr after stimulation (Two-way ANOVA; Tukey’s multiple comparison test ***p<0.001). All values are given as mean ± SEM. Source data is provided in Figure 8—source data 1. (**J**) Epileptic spike rates for each minute (one data point, color-coded for each animal) in a 3-hr recording. (**K, L**) An initial 30 min eLFS and 10 min ‘on-off’ stimulation protocol abolishes epileptic spikes during the stimulation cycle. Approximately 40 min after the last 10 min eLFS the epileptic spike rate starts to rise again. Figure 8—source data 1.eLFS effect on ipsilateral epileptiform activity over time.(**A, B**) High-load burst ratios and epileptic spike rates of each sub-session for the first and the second week of daily 1 Hz eLFS are listed. The high-load burst ratio and epileptic spike rate are reduced during eLFS but recover within the first hour of post-recording (post 1). (**C**) High-load burst ratios of reference LFP recordings (days 33 and 41 after SE) for 3 hr of individual animals. (**D**) High-load burst ratios for individual animals that went into 3-hr continuous eLFS experiments. Epileptiform activity is effectively reduced during ongoing eLFS but returns in a reduced manner within the first hour in some animals. The implant of EP167 broke after the last reference LFP recording and was therefore excluded. Values are given as mean ± SEM.

To assess whether the seizure-suppressive effect of 1 Hz LFS was stable over extended periods, we repeatedly applied eLFS over 3 weeks after the preconditioning experiments (Figure 1B). In the first and second week (weeks 5 and 6 after SE), animals were stimulated and recorded daily for 4 hr (including ‘pre’-, ‘eLFS’-, ‘post 1’-, and ‘post 2’-sessions of 1 hr each). Three-hour reference LFP recordings were performed before, in between, and after these eLFS experiments (Figure 8A). During these 2 weeks, spontaneous epileptiform activity was successfully suppressed in each eLFS session but returned to reference level during the following 2 hr (Figure 8B–G, Figure 8—source data 1). Compared to the first reference recording, epileptiform activity remained unchanged in the intermittent reference recordings (Figure 8H, Figure 8—source data 1; Figure 8J).

**Figure 8—figure supplement 1.**
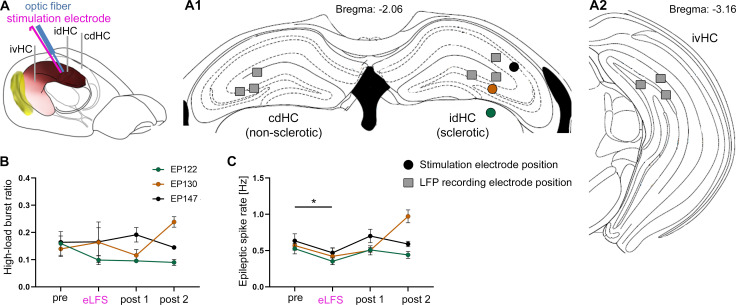
eLFS outside of the dentate gyrus is ineffective in suppressing spontaneous epileptiform activity. (**A**) Implantation scheme. (**A1**) Dorsal electrode (square) and optic fiber/stimulation electrode (dot) positions for three animals in which the stimulation electrode was misplaced during the implantation surgery. (**A2**) Ventral electrode positions. (**B**) In the idHC, the high-load burst ratio was not reduced during 1-hr eLFS (two sessions per animal). (**C**) The epileptic spike rate was slightly reduced compared to the ‘pre’ session. Single sessions were used to calculate the one-way ANOVA; Tukey’s multiple comparison test *p<0.05 (n = 3 animals).

**Figure 8—figure supplement 2.**
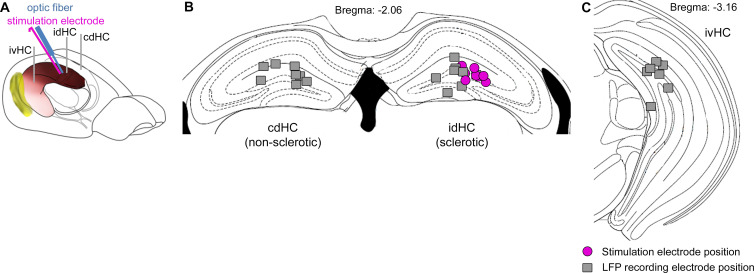
Positions of implanted electrodes for all animals included in the study. (**A**) Implantation scheme for eLFS combined with optogenetic seizure induction. The stimulation electrode was glued to the optic fiber and both were implanted in a 30° angle at the idHC site. (**B**) Positions of the stimulation electrode/optic fiber (pink circle) and LFP electrodes in the dorsal region (n = 8). (**C**) Ipsilateral ventral electrode positions.

In the third week (week 7 after SE), we extended the eLFS period from 1 hr to 2 hr on 2 subsequent days (Figure 8A). Here, we confirmed that stimulation was also effective for 3-hr stimulation periods. Within 1 hr following eLFS, high-load bursts gradually reappeared (Figure 8I, Figure 8—source data 1). Finally, we tested whether we could reduce the number of 1 Hz stimuli and still maintain the successful interference with epileptiform activity. Therefore, we introduced stimulation-free periods and stimulated mice on 2 consecutive days in an on-off manner: after an initial 30 min stimulation period, four 10 min ‘off’ and ‘on’ phases, and a 1-hr ‘post’ recording followed. Epileptiform activity was efficiently extinguished during the 2-hr discontinuous stimulation (‘on-off’ period, Figure 8K,L), showing the same suppressive effect as the continuous stimulation.

Interestingly, mice with a misplaced stimulation electrode (i.e. not in the dentate gyrus, Figure 8—figure supplement 1A, n = 3) showed no reduction of the high-load burst ratio (Figure 8—figure supplement 1B, pre: 0.16 ± 0.02; eLFS: 0.15 ± 0.03; post 1: 0.15 ± 0.02; post 2: 0.17 ± 0.03; n = 3 animals) and only a slight reduction of the epileptic spike rate during eLFS (Figure 8—figure supplement 1C, pre: 0.59 ± 0.05 Hz; eLFS: 0.43 ± 0.05 Hz; post 1: 0.60 ± 0.06; post 2: 0.71 ± 0.10; pre vs. eLFS p<0.01; n = 3 animals). This suggests that the stimulation target, i.e. the dentate gyrus, is important for a successful seizure interference in the intrahippocampal KA mouse model (Figure 8—figure supplement 2).

In conclusion, eLFS in the sclerotic hippocampus has a strong seizure-suppressive effect, which (i) maintains its efficacy over several weeks without desensitization, (ii) can be prolonged for at least 3 hr, and (iii) remains stable in short stimulation-free phases.

## Discussion

In the present study, we applied LFS in experimental MTLE to interfere with seizure generation *in vivo*. Our main findings can be summarized as follows: (1) we modified the intrahippocampal KA mouse model to create different degrees of hippocampal sclerosis and seizure burden, (2) in this model, among several frequencies, 1 Hz stimulation exerts the strongest suppressive effects on both spontaneous subclinical epileptiform activity and evoked generalized seizures, (3) the suppressive action of LFS is stable during the applied stimulation time and does not fade over several weeks, and (4) this effect is most likely associated with reduced efficacy of perforant path transmission onto DGCs rather than downscaling their intrinsic excitability.

### Modification of a mouse epilepsy model to obtain variable degrees of hippocampal sclerosis and seizure burden

To create an animal model that reflects the inter-individual variability of neurodegeneration and seizure frequency as observed in human MTLE (Blümcke et al., 2013; Thom, 2014), we modified the well-established intrahippocampal KA mouse model of MTLE by varying the KA dose. We found that different KA concentrations resulted in variable degrees of histopathological changes and the development of spontaneous recurrent epileptiform activity as previously described for 20 mM KA injection (Janz et al., 2017b). In particular, cell death of hilar neurons increased with the KA dose, whereas DGCs survived equally but dispersed more strongly. This is in line with previous reports from human patients, showing that GCD appeared to be related to the amount of cell loss in the hilus (Houser, 1990) and that DGCs survive in the sclerotic tissue (Thom, 2014). Similar to the human pathology, the sclerotic hippocampus in the intrahippocampal KA mouse model is thought to be critically involved in seizure generation (Krook-Magnuson et al., 2015; Pallud et al., 2011; Walker, 2015). Accordingly, we observed a correlation between hilar cell loss, GCD, and epileptiform activity in the ipsilateral hippocampus.

Epileptiform activity generated in the sclerotic focus often spreads to the contralateral hippocampus in MTLE patients (Gloor et al., 1993; Mintzer et al., 2004) and KA-treated mice (Janz et al., 2018; Meier et al., 2007). We observed that epileptiform activity propagated less frequently to the contralateral hippocampus in mice with strong hippocampal sclerosis, than in those with mild sclerosis, pointing toward a relationship between network preservation and seizure spread.

### oLFS of entorhinal afferents interferes with spontaneous epileptiform activity

We used the modified KA mouse model to stimulate the sclerotic hippocampus for seizure interference. Following KA injection, CA1 and CA3 pyramidal cells are extensively lost (Bouilleret et al., 1999; Marx et al., 2013), whereas DGCs and their entorhinal afferents are preserved (Janz et al., 2017a). Accordingly, by positioning the optic fiber as precisely as possible above the molecular layer of the dentate gyrus, we stimulated mainly the perforant path (Janz et al., 2018). Hence, CA2 neurons, surviving CA3 pyramidal cells, or temporoammonic fibers (in cases of mild hippocampal sclerosis) were most likely not activated by light delivery. Using this experimental design, we identified 1 Hz oLFS as a highly effective stimulation frequency to suppress spontaneous epileptiform activity in the sclerotic and also in the undamaged, contralateral hippocampus. Interestingly, we recorded population spikes at both the site of light delivery as well as contralaterally with a polysynaptic delay, pointing to a more widespread network effect.

LFS at 1 Hz has been used previously to alleviate epileptiform activity. *In vitro* approaches showed that optogenetic activation of calcium/calmodulin-dependent protein kinase type II (CaMKII)-positive neurons for 3 min in the entorhinal cortex and electrical stimulation of the ventral hippocampal commissure for 15 min disrupt seizure-like activity induced by 4-Aminopyridine (4-AP) (Shiri et al., 2017; Toprani and Durand, 2013). Also, electrical 1 Hz stimulation of Schaffer collaterals in acute hippocampal slices for 10 min diminished epileptiform activity induced by bicuculline (Albensi et al., 2004). In addition to this short-term efficacy of 1 Hz LFS, long-term effects over several hours (60 min continuous stimulation or alternating periods of 15 min on/off for 4 hr) were reported from *in vivo* studies using amygdala kindling or pilocarpine rodent models targeting the ventral hippocampal commissure or entorhinal cortex (Rashid et al., 2012; Xu et al., 2016). Although these reports give valuable insights into the seizure-suppressing effects of LFS, they differ from our study with respect to the epilepsy models and stimulation targets used. Our emphasis was to show that LFS can prevent seizure generation in chronically epileptic mice with hippocampal sclerosis.

Optogenetics allows us to selectively target entorhinal afferents in the hippocampus for our oLFS experiments. Optogenetic stimulation has been applied in the past to alleviate seizure-like activity in experimental epilepsy (for review see Christenson Wick and Krook-Magnuson, 2018; Krook-Magnuson and Soltesz, 2015; Zhao et al., 2015). Most optogenetic studies decreased network activity either by direct inhibition of hippocampal principal cells or by activating GABAergic interneurons (Kokaia et al., 2013; Krook-Magnuson et al., 2013; Ladas et al., 2015; Ledri et al., 2014). More recently, Bui et al., 2018 showed that optogenetic stimulation of mossy cells in the dorsal dentate gyrus can control electrographic seizures, but the effect was much weaker than with direct inhibition of DGCs.

In the present study, by indirectly stimulating excitatory DGCs we deviated from the approach of increasing inhibition. Accordingly, local 1 Hz photostimulation of entorhinal afferents effectively suppressed epileptiform activity in both hippocampi independent of the severity of hippocampal sclerosis. Lower stimulation frequencies (0.5 and 0.2 Hz) were less effective. This is in line with previous *in vitro* studies showing a frequency dependency for the reduction of the duration of 4-AP-induced seizure-like activity (D'Arcangelo et al., 2005; Toprani and Durand, 2013).

### Preconditioning by LFS prevents induced generalized seizures

Next, we probed whether oLFS and eLFS can interfere with behavioral seizures induced by 10 Hz photostimulation of entorhinal afferents, as 5–20 Hz hippocampal photostimulation has previously been shown to be pro-convulsive (Janz et al., 2018; Osawa et al., 2013). We observed that 1 Hz stimulation right after a pro-convulsive stimulus, which is similar to an on-demand approach, was not able to interrupt an ongoing seizure. Similarly, on-demand photostimulation of DGCs at 7 or 20 Hz exacerbated spontaneous epileptiform activity to large behavioral seizures in the same model (Bui et al., 2018; Krook-Magnuson et al., 2015), and on-demand inhibition of DGCs (or mossy cells) did not affect the occurrence of behavioral seizures (Bui et al., 2018).

However, preconditioning by 1 Hz (oLFS or eLFS) or 0.5 Hz (oLFS) for 30 min was able to prevent the occurrence of optogenetically-induced seizures. Our preconditioning results are in line with a technique called quenching that blocked the development and progression of amygdala-kindled seizures using 15–20 min 1 Hz stimulation right before or after a kindling stimulus or daily LFS in fully kindled rats (Jalilifar et al., 2018; Weiss et al., 1995). Taken together, timing and frequency of stimulation appear to be critical for successful seizure interference.

### The suppressive effects of eLFS on epileptiform activity are stable over several weeks

Considering the clinical limitations of optogenetics, we applied eLFS in the dentate gyrus over several weeks to test the suppressive action on epileptiform activity. We first repeated the 1-hr stimulation sessions daily over 2 weeks. Then, we applied eLFS for longer periods (3 hr) in the third week. Finally, since continuous LFS could potentially disrupt hippocampal function and shorten the battery life of implanted devices, we applied a discontinuous stimulation protocol. All of these stimulation protocols were repeatedly effective in suppressing spontaneous epileptiform activity. In the future, we plan to extend the LFS-off periods and develop a closed-loop system that initiates the 1 Hz stimulation when epileptiform activity returns. This will overcome the limitation of the current study in which the seizure-suppressive effects of LFS have been analyzed over relatively short durations (hours) as longer durations (days or weeks) would be closer to clinical applications.

Our results are promising when compared to the use of anti-epileptic drugs (AEDs) which only acutely exert seizure-suppressive effects in epileptic mice (Duveau et al., 2016; Klein et al., 2015; Riban et al., 2002) but can lose their effects during prolonged treatment (Roucard et al., 2010). Also, MTLE is frequently pharmacoresistant in humans (Engel, 2001). From a translational point of view, selective stimulation of entorhinal afferents might be applied in these patients by placing electrodes in the clearly defined angular bundle (Zeineh et al., 2017).

### LFS reduces the efficacy of perforant path transmission onto DGCs

Although the mechanism underlying the seizure-suppressive effect of LFS is not clear, several lines of evidence suggest that LFS reduces the excitability of the hippocampus and associated networks (i.e. the entorhinal cortex). Besides, retrograde firing of entorhinal cortex neurons may potentially lead to more widespread network effects. Mechanistically, it is rather remarkable that synchronization of the hippocampal network by LFS interferes with seizure generation, a process that is classically characterized by hypersynchronization. One explanation could be the local recruitment of GABAergic interneurons (Xu et al., 2016). However, this is unlikely, since the frequency of IPSCs was strongly reduced in our slice experiments and interneurons are often extensively diminished in the sclerotic hippocampus (Bouilleret et al., 2000; Marx et al., 2013).

Another hypothesis, investigated in a 4-AP *in vitro* slice model, suggests that electrical stimulation of the entorhinal cortex at low frequencies (e.g. at 0.5 Hz) results in less accumulation of extracellular potassium compared to the large potassium efflux associated with GABA_A_ receptor-mediated interictal (between seizures [Fisher et al., 2014]) discharges that could trigger a seizure (Avoli et al., 2013). Clamping GABA-mediated potentials with 1 Hz LFS (directly via activation of GABAergic interneurons or indirectly via feedback inhibition upon principal cell activity) could therefore induce transient increases in extracellular potassium and restrain pro-epileptic discharges (Barbarosie et al., 2002). In other words, stimulating the network more frequently (i.e. at 1 Hz) leads to a lower accumulation of extracellular potassium than less frequent interictal discharges would induce (Shiri et al., 2017). Our results show that this mechanism cannot fully account for the seizure-suppressive effect of oLFS since we observed a decrease in the amplitude of evoked responses in the presence of GABA_A_ and GABA_B_ receptor blockers in whole-cell recordings.

Another explanation for the reduction of epileptiform activity achieved by our experimental design could be synaptic depression due to synaptic fatigue or long-term depression (LTD). Synaptic depression can be induced at the entorhinal–DGC synapse with stimulation frequencies equal to or below 1 Hz (Abrahamsson et al., 2005; Gonzalez et al., 2014). In line with this, we showed that the amplitude of evoked responses decreases over time on both the network and the single-cell level, which could explain the prolonged interval without spontaneous epileptiform activity following LFS. However, the intrinsic properties of DGCs did not change, thus there were no obvious signs of postsynaptic LTD. It is unlikely that downscaling of entorhinal inputs alone is sufficient to suppress epileptiform activity given that perforant path transection does not alleviate seizure-like activity (Meyer et al., 2016; Pallud et al., 2011). We therefore assume that the seizure-suppressive effects of our oLFS cannot fully rely on synaptic depression. Supporting this notion, LFS is not effective in inducing synaptic depression in sclerotic human hippocampal slices (Beck et al., 2000).

Concerning acute seizure-suppressive effects, we suggest that LFS drives the hippocampal network into a stable state, reducing the probability of seizures while LFS is ongoing. This hypothesis is supported by Chang et al., 2018 who showed that evoked interictal-like discharges elicit anti-epileptic effects, contingent on the network state as well as stimulus amplitude and frequency. This study investigated the influence of interictal discharges on seizure generation *in vitro* in local CA3 and CA1 circuits, two subnetworks that are typically lost in MTLE with hippocampal sclerosis. Our results suggest that the authors’ observations also apply to the entorhinal-dentate network *in vivo*. In line with this interpretation, oLFS of entorhinal afferents may introduce glutamatergic synaptic perturbation followed by transient suppression of neuronal activity (de Curtis et al., 2001; de Curtis and Avanzini, 2001; Muldoon et al., 2015) and thus may interfere with recurrent generation of epileptiform activity (Chang et al., 2018).

It is possible that oLFS could impair normal hippocampal function given its dramatic effect on glutamatergic transmission. However, Wang et al., 2020 demonstrated that LFS improved cognitive function in pilocarpine-injected rats. Future research will address whether hippocampal function is affected by long-term LFS in our model.

In conclusion, our study identified 1 Hz LFS in the dentate gyrus as a highly efficient approach to interfere with seizure generation in the MTLE mouse model with hippocampal sclerosis. We demonstrated that the effect was largely driven by repetitive activation of DGCs residing in the sclerotic seizure focus, and we have shed light on the associated cellular mechanisms. Considering the potential for clinical translation, our findings may pave the way for effective seizure control in one of the most common forms of drug-resistant epilepsy.

## Materials and methods

### Animals

Experiments were conducted with adult (8–12 weeks) transgenic male mice (C57BL/6-Tg(Thy1-eGFP)-M-Line) (Feng et al., 2000). Each animal represents an individual experiment, performed once. In total, 96 mice were used for this study. Mice were kept in a 12 hr light/dark cycle at room temperature (RT) with food and water *ad libitum*. All animal procedures were carried out under the guidelines of the European Community’s Council Directive of September 22, 2010 (2010/63/EU) and were approved by the regional council (Regierungspräsidium Freiburg).

### KA and virus injections

Mice were injected with KA or saline into the right dorsal hippocampus (KA: n = 53, saline: n = 5 for *in vivo* experiments and KA: n = 34, saline: n = 4 for acute slice electrophysiology), as described previously (Heinrich et al., 2006; Häussler et al., 2012; Janz et al., 2017a). In brief, mice were deeply anesthetized (ketamine hydrochloride 100 mg/kg, xylazine 5 mg/kg, atropine 0.1 mg/kg body weight, i.p.) followed by a stereotaxic injection of 50 nL of either 10, 15, or 20 mM KA solution (Tocris, Bristol, UK) in 0.9% sterile saline or injection of saline only. Mice were randomly assigned to a respective KA concentration group. Stereotaxic coordinates relative to bregma were: anterioposterior (AP) = −2.0 mm, mediolateral (ML) = −1.5 mm, and relative to the cortical surface: dorsoventral (DV) = −1.5 mm. Following KA injection the occurrence of a behavioral SE was verified. SE was characterized by mild convulsion, chewing, immobility, or rotations, as described before (Riban et al., 2002; Tulke et al., 2019). Mice that did not have SE (n = 5) or died due to KA treatment (n = 18) or surgical procedures (n = 2) were excluded from further experiments.

For optogenetic stimulation of entorhinal fibers, KA- and saline-treated animals were stereotaxically injected with a recombinant adeno-associated virus (AAV, 0.45 µl; n = 50 for *in vivo* experiments and n = 28 for acute slice electrophysiology), carrying the genomic sequences for ChR2 fused to mCherry under the control of the CaMKIIa promoter (AAV1.CaMKIIa.hChR2(H134R)-mCherry.WPRE.hGH; Penn Vector Core, Pennsylvania, USA) into the ipsilateral medial entorhinal cortex in the same surgery (Janz et al., 2018). Stereotaxic coordinates relative to bregma: AP = −5.0 mm, ML = −2.9 mm, and relative to the cortical surface: DV = −1.8 mm. KA-injected mice without virus injection were used as controls (no-virus controls, n = 5).

### Implantations

For *in vivo* experiments Teflon-coated platinum-iridium wires (125 µm diameter; World Precision Instruments, Sarasota, Florida, USA) were implanted 16 days after injections at three hippocampal positions as described previously (Janz et al., 2018): ipsilateral dorsal (idHC), ipsilateral ventral (ivHC) and contralateral dorsal (cdHC). All animals were additionally implanted with an optic fiber (ferrule 1.25 mm, cannula 200 µm diameters; Prizmatix Ltd., Givat-Shmuel, Israel) in the same position as the idHC electrode, but at a 30° angle. For electrical stimulation, 11 animals received an additional electrode (coated with nanostructured platinum [Boehler et al., 2020]), in parallel to the optic fiber. Stereotaxic coordinates are given relative to bregma (AP, ML) or to the cortical surface (DV): cdHC: AP = −2.0 mm, ML = +1.4 mm, DV = −1.6 mm; idHC: AP = −2.0 mm, ML = −1.4 mm (−2.4 mm for the optic fiber), DV = −1.6 mm; and ivHC: AP = −3.4 mm, ML = −2.8 mm, DV = −2.1 mm. The correct positions of electrodes and optic fibers were confirmed by histology (Figure 8—figure supplement 2). Three mice had to be excluded due to wrong electrode positions (Figure 8—figure supplement 1). Two stainless steel screws (DIN 84; Schrauben-Jäger, Landsberg, Germany) were implanted above the frontal cortex to provide a reference and ground, respectively. Electrodes and screws were soldered to a micro-connector (BLR1-type). The implant was fixed with dental cement (Paladur).

### *In vivo* LFS experiments

After recovery from implantations, the freely behaving mice were first recorded on 2 successive days (3 hr each) to determine reference LFPs (Figure 1). Each mouse represents the biological replicate and the number of recordings per mouse the technical replicate. For LFP recordings, mice were connected to a miniature preamplifier (MPA8i, Smart Ephys/Multi-Channel Systems, Reutlingen, Germany). Signals were amplified 1000-fold, bandpass-filtered from 1 Hz to 5 kHz, and digitized with a sampling rate of 10 kHz (Power1401 analog-to-digital converter, Spike2 software, Cambridge Electronic Design, Cambridge, UK).

#### Optogenetic stimulation

On days 21–28 after KA and virus injection (10, 15, or 20 mM KA, AAV1.CamKIIa.ChR2-mCherry), photostimulation with pulsed blue light (460 nm; 50 ms pulse duration; 150 mW/mm² at the fiber tip; blue LED, Prizmatix Ltd.) was applied at low frequencies (1, 0.5, or 0.2 Hz) to test the effect of oLFS on spontaneous epileptiform activity. Taking into account the restricted penetration depth of 473 nm light in brain tissue (Yizhar et al., 2011) and an output of 150 mW/mm² at the tip of our optic fiber, we estimated that in our case the radius of light emission with sufficient power to activate ChR2 did not exceed 500 µm (Figure 4—figure supplement 2). During stimulation, we continuously recorded LFPs and videos. Recording sessions were divided into four sub-sessions: ‘pre’ - 1 hr before oLFS; ‘oLFS’ - 1 hr during oLFS; ‘post 1’ and ‘post 2’ - first hour and second hour after oLFS (Figure 1A, week 4). For each frequency, we performed 2 trials on different days. To check for light- or heat-induced effects the ‘pre’ and ‘oLFS’ (1 Hz) sessions were performed in non-virus controls.

Since in the intrahippocampal mouse model spontaneous epileptiform activity is frequent but rarely generalizes we evoked behavioral seizures by 10 Hz photostimulation (25 ms pulse duration) and assessed the effect of oLFS before or after the pro-convulsive 10 Hz stimulus (Figure 1A, week 5). To determine the minimum duration sufficient to trigger a behavioral seizure for each animal (identification of seizure threshold), we systematically increased the stimulation duration (in 1 s steps). Each evoked seizure was visually inspected on the electrophysiological as well as behavioral level. We assessed generic features as described by Jirsa et al., 2014 and motor symptoms according to the Racine scale (Racine, 1972). The identified seizure threshold was then validated for robust seizure induction for at least 3 times in each animal. In subsequent ‘preconditioning' oLFS sessions, photostimulation at 1 or 0.5 Hz was performed for 30 min before applying the pro-convulsive 10 Hz stimulus. For both frequencies, a minimum of 3 trials was performed on different days. In a subset of mice (n = 5), the efficacy of the pro-convulsive 10 Hz stimulus was tested again after the preconditioning experiments to exclude confounding effects of habituation.

#### Electrical stimulation

To investigate the long-term effectiveness of hippocampal LFS, we applied electrical stimulation *in vivo* (Figure 1B). Reference LFPs were recorded for 3 hr (Figure 1B, week 3) 19–21 days after injections (15 mM KA, AAV1.CamKIIa.ChR2-mCherry), that is immediately before stimulation experiments, and after weeks 5 and 6. Stimulation experiments consisted of 30 min eLFS (biphasic rectangular current pulses: 400 μs phase duration, ±200 μA amplitude, anodic first; MC stimulus II software, STG1004, Smart Ephys/Multi-Channel Systems, Reutlingen, Germany) followed by optogenetic 10 Hz seizure induction (seizure threshold was determined as described above, Figure 1B, week 4). Then, we applied eLFS to interfere with spontaneous epileptiform activity: mice were electrically stimulated daily for 1 hr (1 Hz) over 2 weeks (2 × 5 days) during LFP recordings following our standard paradigm (‘pre’, ‘eLFS’, ‘post 1’ and ‘post 2’, 1 hr each, Figure 1B, weeks 5–6). In week 7, mice were stimulated continuously by eLFS for 3 hr after a 1-hr ‘pre’ recording on 2 successive days. Finally, we stimulated intermittently: mice were stimulated initially for 30 min continuously followed by 10 min ‘off’ (no stimulation) and 10 min ‘on’ (1 Hz stimulation) phase, which was repeated four times, followed by 1-hr ‘post’ recording (Figure 1B, week 7).

### Perfusion and tissue preparation

Following the last recording session, mice were anesthetized (see above) and transcardially perfused [0.9% saline followed by 4% paraformaldehyde in 0.1 M phosphate buffer (PB, pH 7.4)]. Following dissection, brains were post-fixated overnight, immersed in sucrose (25% in PB) overnight at 4°C for cryo-protection, shock-frozen in isopentane at −40°C and stored at −80°C. Brains were sectioned (coronal plane, 50 μm) with a cryostat (CM3050, Leica, Bensheim, Germany). Slices were collected in 2x saline-sodium citrate buffer (2xSSC; 0.3 M NaCl, 0.03 M sodium citrate, pH 7.0).

### Fluorescent *in situ* hybridization (FISH)

*Gad67* mRNA was localized by FISH with digoxigenin (DIG)-labeled cRNA probes generated by *in vitro* transcription as described earlier (Kulik et al., 2003). Slices were hybridized with DIG-labeled antisense cRNA probes and immunodetection of the DIG-labeled hybrids was performed with a peroxidase-conjugated anti-DIG antibody (1:2000; raised in sheep; Roche Diagnostics, Mannheim, Germany). The fluorescence signal was developed with tyramide signal amplification (TSA) Plus Cyanine 3 System kit (PerkinElmer, Waltham, Massachusetts, USA) as described previously (Tulke et al., 2019).

In detail, brain slices were pre-treated in a 1:1 mixture of hybridization buffer [50% formamide, 4xSSC, 5% dextran sulfate, 250 μg/ml heat-denatured salmon sperm DNA, 200 µl yeast t-RNA, 1% Denhardt's-reagent (Sigma-Aldrich, Steinheim, Germany)] and 2xSSC at RT for 15 min. Subsequently, the slices were pre-hybridized in a hybridization buffer for 60 min at 45°C, followed by the addition of DIG-labeled antisense or sense *Gad67* cRNA probe (100 ng/ml) and incubated overnight at 45°C. Slices were washed in 2xSSC for 2 × 15 min at RT and then successively rinsed at 55°C for 15 min in 2x SSC with 50% formamide; 0.1x SSC with 50% formamide and twice in 0.1x SSC alone. Then the slices were rinsed in 0.1 M Tris-buffered saline (TBS) for 2 × 10 min and transferred to the blocking buffer [1% blocking reagent (Roche Diagnostics, Mannheim, Germany) in TBS] for 60 min at RT. For fluorescent detection, tissue sections were treated with a horseradish peroxidase-conjugated DIG antibody overnight at 4°C and developed in the presence of amplification buffer and tyramide working solution (1:50) for 6 min in the dark. The staining-reaction was stopped by rinsing in TBS for 3 × 5 min and 1 × 15 min. Slices were kept in the dark for further immunofluorescence staining.

### Immunohistochemistry

For immunofluorescence staining, free-floating sections were pre-treated in 10% normal horse serum (Vectorlabs, Burlingame, California, USA) in phosphate buffer (PB) for 1 hr. Subsequently, slices were incubated first with guinea-pig anti-NeuN (1:500; Synaptic Systems, Göttingen, Germany) overnight at 4°C and then with a donkey anti-guinea-pig Cy5-conjugated antibody for 2.5 hr at RT (1:200, Jackson ImmunoResearch Laboratories Inc, West Grove, Pennsylvania, USA) followed by extensive rinsing in PB. Sections were mounted on glass slides with an anti-fading mounting medium (DAKO, Hamburg, Germany).

### Image acquisition and analysis

Composite images were taken with an AxioImager2 microscope using Plan-APOCHROMAT 5x or 10x objectives (Zeiss, Göttingen, Germany). Exposure times (5x objective: Cy5-labeled NeuN, 500 ms; 10x objective: Cy3-labeled *Gad67* probe, 700 ms, Cy5-labeled NeuN, 5 s) were kept constant for each staining. The images were further processed with ZEN blue software (Zeiss).

To assess the extent of hippocampal sclerosis, we quantified the volume of the dispersed granule cell layer (GCL) and cell loss in the hilus and CA1 along the septo-temporal axis of the hippocampus using Fiji ImageJ software (Schindelin et al., 2012). Here, masking was performed since the evaluator was not aware of the respective KA treatment. In detail, a region-of-interest (ROI) was drawn visually comprising the dispersed parts of the GCL using the ImageJ polygon function in each slice (around 50 slices per animal). Afterwards, the volume was calculated based on the area measured in each slice (values are given in mm^3^). For the quantification of cell loss, the summed length of pyramidal cell-free gaps in the CA1 region was measured in each section using the segmented line function. Furthermore, hilar cell loss was quantified by automated detection (Cell Counter plugin) of NeuN^+^ cells (size parameter: 50-infinity pixel^2^) and *Gad67* mRNA^+^ interneurons (size parameter: 100-infinity pixel^2^) in the hilus of three dorsal sections for each animal. Here, we calculated the percentage of cell loss in the sclerotic hippocampus compared to the contralateral, non-sclerotic hippocampus (set to 100%). In detail, the Cy3 and Cy5 channels were split and individual images were converted to grayscale. Images were background-subtracted by manual adjustment of the threshold and further processed using the watershed function, which separates overlapping cell bodies. A ROI was then defined for the hilus with the polygon function, and the respective cell density was calculated. ChR2-mCherry expression analysis was performed using the mean gray value for each layer along the dorsoventral axis of the sclerotic hippocampus (Figure 4—figure supplement 2E) in Fiji ImageJ. We manually selected three ROIs in each layer (from alveus to the lower outer molecular layer of the dentate gyrus) in the image of the hippocampus where the optic fiber was positioned.

### Analysis of epileptiform activity

Animals that showed abnormal hippocampal atrophy (i.e. extensive loss of DGCs) were excluded from analyses (n = 5). Recordings obtained from all electrodes were first visually inspected for epileptiform activity. Then, we used a custom algorithm developed in the intrahippocampal KA mouse model (Heining et al., 2019) to detect and classify epileptiform activity in LFP data from idHC and cdHC. The dataset used from Heining et al., 2019 served us as a reference for classification. This reference dataset consisted of data obtained from animals injected with 20 mM KA recorded at electrode positions comparable to ours. The detection and classification algorithm works as described in the following: *Epileptiform spikes:* The spectrogram per frequency bin was normalized to values between 0 and 1 and local maxima in the normalized (4–40 Hz) frequency band were identified as spikes. This was complemented by amplitude-based spike detection and spike sorting to reduce false negatives or positives. *Bursts*: Spikes within an inter-spike interval below 2.5 s were assigned to the same burst. *Feature vectors:* Each burst *i* was represented by a three-dimensional feature vector *y_i_* (= [log10 of spike count; log10 of mean inter-spike interval; standard deviation of inter-spike intervals]). To compare our data to the reference dataset, *y_i_* was normalized to the mean (*x̄*) and standard deviation (σ) of the feature vectors *x* of the reference dataset and multiplied by the weight vector *w* (=[2; 2; 1]):

yi'=(yi−x¯)/σx⋅w

*Burst classification*: bursts were classified using a SOM that consists of nodes (visualized as hexagons) representing prototypical feature vectors. Nodes with similar feature vectors are neighbors on the SOM. Based on the SOM created with the reference dataset, bursts were classified according to their spike load into high-load, medium-load, and low-load bursts categories (Figure 3—figure supplement 1A,B). To classify burst*s i* we matched *y_i_’* to the closest node (Euclidean distance) of the SOM. Burst *i* then inherited the category of this best matching node (Figure 3—figure supplement 1C).

To assess the severity of epileptiform activity within a recording, we calculated the high-load burst ratio as the fraction of time spent in high-load bursts (sum of the high-load burst durations divided by total recording time). The automatic detection of high-load bursts was confirmed by visual inspection. To assess epileptic burden of individual mice in the three KA concentration groups, the average high-load burst ratio was calculated from a total of nine LFP recordings (15 hr) performed on different days (2x 3 hr before oLFS experiments, 6x 1 hr of ‘pre’ sub-sessions of oLFS experiments and 1x 3 hr after oLFS experiments; Figure 1A). In oLFS and eLFS experiments, suppressive effects on epileptiform activity were evaluated based on the high-load burst ratio and the epileptic spike rate calculated for the respective sub-session (‘pre’,’ oLFS’/’eLFS’,’ post 1’, post 2’). Whole sessions were excluded if the corresponding ‘pre’ recording had a high-load burst ratio below 0.05.

Furthermore, we analyzed the AUC of evoked responses (integral of LFP traces in a defined time interval) during photostimulation. We used a 4th order low-pass Chebyshev filter type I with a cut-off frequency of 300 Hz to smoothen the signal. The time interval for calculating the AUCs was set relative to stimulus onset from −0.1 s to +0.2 s for oLFS (1, 0.5, and 0.2 Hz) and from −0.02 s to +0.06 s for the 10 Hz stimulation (Figure 5—source code 1, Figure 6—source code 1). For responses during oLFS that were co-occurring with high-load bursts, the AUC was not determined. Recordings obtained from the cdHC site were used to measure pulse latencies between recording sites during photostimulation sessions (Figure 4—figure supplement 4—source code 1). In brief, we used the same time intervals and filters as applied for the AUC calculation and z-scored the voltage curve of each pulse response. We then calculated the delay between each idHC and cdHC pulse response using the first threshold crossing (one threshold was identified by visual inspection of the signal for all animals) and took the median for each oLFS sub-session.

### Acute slice electrophysiology

In an additional set of experiments, mice were deeply anesthetized 21 to 28 days after KA (15 mM) and virus injection, perfused with 10 ml cold protective solution containing (in mM): 92 choline chloride, 30 NaHCO_3_, 2.5 KCl, 1.2 NaH_2_PO_4_, 25 D-glucose, 20 HEPES, 0.5 CaCl_2_, 5 Na-ascorbate acid, 2 Thiourea, 3 Na-Pyruvate, 10 MgCl_2_ and 12 N-acetylcysteine (oxygenated with 95% O_2_/5% CO_2_, 34°C) before dissection. Transverse acute hippocampal slices (300–350 µm) were obtained and incubated for 1 hr at 34°C in a solution in which choline was replaced by 1 N-Methyl-D-glucamine (NMDG). Afterward, slices were stored in artificial cerebrospinal fluid (ACSF, containing in mM: 125 NaCl, 25 NaHCO_3_, 2.5 KCl, 1.25 NaH_2_PO_4_, 25 D-glucose, 2 CaCl_2_, and 1 MgCl_2_ oxygenated with 95% O_2_/5% CO_2_) and supplemented with 12 mM N-acetylcysteine at RT. Whole-cell patch-clamp recordings were performed as previously described (Elgueta et al., 2015) in the presence of GABA_A_ and GABA_B_ receptor blockers [10 µM gabazine and 2 µM CGP55845, respectively; 30–34°C; Multiclamp 700B amplifier (Molecular Devices, San José, California, USA); 5 kHz low-pass filter; sampling frequency 40 kHz]. When recording IPSCs, the extracellular solution was supplemented with 10 µM CNQX and 100 µM APV to block excitatory postsynaptic currents. Stimulus generation, data acquisition, and analysis were performed using custom-made programs written in Igor (WaveMetrics Inc, Portland, Oregon, USA). Recording pipettes were filled with a solution containing (in mM): 140 K-Gluconate, 4 KCl, 10 HEPES, 2 MgCl_2_, 2 Na_2_ATP, 10 EGTA, 0.125 Alexa-Fluor 488 and 0.15 biocytin (pH = 7.2; 290–310 mOsm), that resulted in pipette resistances of 4–6 MΩ. Series resistances between 8 and 20 MΩ were compensated using bridge balance in current-clamp and were left uncompensated in voltage-clamp. For loose-patch experiments and extracellular stimulations, pipettes were filled with a HEPES-buffered ACSF (containing in mM: ACSF (see above), 135 NaCl, 5.4 KCl, 1.8 CaCl_2_, 1 MgCl_2_, 5 HEPES). sIPCS were recorded at +20 mV when using an intracellular solution contained (in mM) 130 Cs-Gluconate, 10 HEPES, 2 MgCl_2_, 2 Na_2_ATP, 10 EGTA, 8 TEA-Cl, and 10 EGTA. Extracellular stimulation was performed using a stimulus isolator (Isopulser) with pipettes (~1 MΩ) placed in the middle molecular layer where the ChR2-mCherry was expressed. Five pulses (50 Hz, 0.1–0.3 ms, 20–100 V) were evoked and a minimum of 10 trials was used to calculate the overall discharge probability. The rheobase was measured with 1 s-long current injections increasing with 20 pA steps. Series resistance, cell capacitance (C_m_), and membrane resistance (R_m_) were calculated from −10 mV pulses. Photostimulation was performed for 10 min using full-field blue light pulses (473 nm; 50 ms pulse duration; LED p2000, CoolLED, Andover, UK).

### Statistical analysis

Data were tested for significant differences with Prism 8 software (GraphPad Software Inc). Comparisons of two groups were performed with a paired (comparisons within animals) or unpaired (comparisons between animals) Student’s t-test. When more than two groups were compared either one-way ANOVA, or two-way ANOVA followed by Tukey’s post-hoc test was applied. In the case of missing values, statistics were calculated by fitting a restricted maximum likelihood model (REML). Significance thresholds were set to: *p<0.05, **p<0.01 and ***p<0.001. For all sample populations, mean and SEM are given, unless otherwise reported. Correlations were tested using Pearson’s correlation (slope significantly non-zero, CI 95%). All statistical results are summarized in Supplementary file 1.

## Data Availability

The LFP dataset is available on Open Science Framework: https://osf.io/uk94m/. The source code files for the seizure detection algorithm is accessible at Zenodo (https://doi.org/10.5281/zenodo.4110614). The source code for the seizure detection algorithm (Heining et al., 2019) was developed using previously published LFP data (Froriep et al., 2012; Janz et al., 2017b). The following datasets were generated: PaschenEElguetaCHeiningKVieiraDMKleisPOrcinhaCHäusslerUBartosMEgertUJanzPHaasCA2019Hippocampal low-frequency stimulation prevents seizure generation in a mouse model of mesial temporal lobe epilepsyOpen Science Framework10.17605/OSF.IO/UK94M10.7554/eLife.54518PMC780038133349333 HeiningK2020Code for detecting and classifying epileptiform activityZenodo10.5281/zenodo.4110614

## Additional files

Supplementary file 1. Quantitative summary of statistically tested parameters.

Transparent reporting form
