# Supplementary material for: Hippocampal low-frequency stimulation prevents seizure generation in a mouse model of mesial temporal lobe epilepsy
Source: eLife. 2020 Dec 22;9:e54518. doi: 10.7554/eLife.54518 (PMC7800381; doi:10.7554/eLife.54518)
Supplement: Figure 3—source data 1. — (a) The occurrence of burst classes elicited by different KA concentrations. In the ipsilateral hippocampus, animals treated with high (15 and 20 mM) KA concentrations exhibit more high-load and fewer low-load events than animals treated with 10 mM KA. Compared to the ipsilateral hippocampus, animals treated with high (especially 20 mM KA) have less high-load seizures on the contralateral side. (b) Mean high-load burst ratio and epileptic spike rate of the different KA groups. The mean ratio of time spent in high-load bursts (mean high-load burst ratio) and epileptic spike rate were significantly lower in the idHC 10 mM KA group compared to the 15 and 20 mM KA group. The mean was calculated from the reference and ‘pre’ recording sessions. Values are given as mean ± SEM. [file elife-54518-fig3-data1.docx]

**a Burst classes**

| **KA Concentration** | **High-load bursts** | **Medium-load bursts** | **Low-load bursts** | **n (animals)** |
| --- | --- | --- | --- | --- |
| 10 mM HCi | 9.94% | 17.33% | 72.73% | 4 |
| **10 mM HCc** | **9.56%** | **15.29%** | **75.14%** | **4** |
| 15 mM HCi | 21.80% | 14.94% | 63.26% | 6 |
| **15 mM HCc** | **14.96%** | **18.40%** | **66.64%** | **6** |
| 20 mM HCi | 17.78% | 15.43% | 66.97% | 5 |
| **20 mM HCc** | **5.42%** | **11.92%** | **82.67%** | **5** |

| **b Mean high-load burst ratio** | | | | |
| --- | --- | --- | --- | --- |
|  | **10 mM** | **15 mM** | **20 mM** | **n (animals)** |
| **Figure 3D: idHC** | **0.06 ± 0.01** | **0.20 ± 0.03** | **0.20 ± 0.02** | **4/6/5** |
| **Figure 3E: cdHC** | **0.05 ± 0.02** | **0.11 ± 0.03** | **0.07 ± 0.02** | **4/6/5** |
|  |  |  |  |  |
| **Mean epileptic spike rate [Hz]** | | | | |
|  | **10 mM** | **15 mM** | **20 mM** | **n (animals)** |
| **Figure 3F: idHC** | **0.32 ± 0.02** | **0.76 ± 0.10** | **0.77 ± 0.09** | **4/6/5** |
| **Figure 3G: cdHC** | **0.28 ± 0.06** | **0.44 ± 0.10** | **0.32 ± 0.03** | **4/6/5** |
|  |  |  |  |  |

**Figure 3–Source Data 1: Variable severity of epileptiform activity elicited by different KA concentrations. (a) The occurrence of burst classes elicited by different KA concentrations.** In the ipsilateral hippocampus, animals treated with high (15 and 20 mM) KA concentrations exhibit more high-load and fewer low-load events than animals treated with 10 mM KA. **Compared to the ipsilateral hippocampus, animals treated with high (especially 20 mM KA) have less high-load seizures on the contralateral side. (b) Mean high-load burst ratio and epileptic spike rate of the different KA groups. The mean ratio of time spent in high-load bursts (mean high-load burst ratio) and epileptic spike rate were significantly lower in the idHC 10 mM KA group compared to the 15 and 20 mM KA group. The mean was calculated from the reference and ‘pre’ recording sessions. Values are given as mean ± SEM.**
