# Supplementary material for: Hippocampal low-frequency stimulation prevents seizure generation in a mouse model of mesial temporal lobe epilepsy
Source: eLife. 2020 Dec 22;9:e54518. doi: 10.7554/eLife.54518 (PMC7800381; doi:10.7554/eLife.54518)
Supplement: Figure 4—source data 1. — Burst ratios and epileptic spike rates of each sub-session are listed for the three KA concentrations (10, 15, 20 mM) for each oLFS frequency (1, 0.5, 0.2 Hz) and no-virus controls (Ctr.). (a, b) The bust ratio and epileptic spike rate are reduced in all KA groups during oLFS but recover within the first hour of post-recording (post 1). This effect is also observed after 0.5 and 0.2 Hz oLFS but less pronounced. No change is observed in no-virus control animals. (c, d) Summary for all KA groups merged. (e) Median (±95% CI) of the suppression efficacy for the three applied frequencies. oLFS at 1 Hz seems most effective for the suppression of epileptiform activity. Values are given as mean ± SEM. [file elife-54518-fig4-data1.docx]

| **a Burst ratio** | | | | | |
| --- | --- | --- | --- | --- | --- |
| **KA conc.** | **pre** | **1 Hz oLFS** | **post 1** | **post 2** | **n (sessions)** |
| 10 mM | 0.08 ± 0.01 | 0.01 ± 0.00 | 0.11 ± 0.03 | 0.10 ± 0.03 | 6 |
| 15 mM | 0.18 ± 0.03 | 0.05 ± 0.03 | 0.16 ± 0.04 | 0.23 ± 0.05 | 12 |
| 20 mM | 0.19 ± 0.04 | 0.02 ± 0.00 | 0.17 ± 0.04 | 0.21 ± 0.05 | 7 |
|  |  |  |  |  |  |
| **KA conc.** | **pre** | **0.5 Hz oLFS** | **post 1** | **post 2** | **n (sessions)** |
| 10 mM | 0.11 ± 0.00 | 0.00 ± 0.00 | 0.03 ± 0.00 | 0.27 ± 0.00 | 1 |
| 15 mM | 0.24 ± 0.02 | 0.09 ± 0.03 | 0.17 ± 0.04 | 0.28 ± 0.04 | 9 |
| 20 mM | 0.21 ± 0.03 | 0.07 ± 0.02 | 0.13 ± 0.05 | 0.26 ± 0.05 | 4 |
|  |  |  |  |  |  |
| **KA conc.** | **pre** | **0.2 Hz oLFS** | **post 1** | **post 2** | **n (sessions)** |
| 10 mM | 0.10 ± 0.04 | 0.05 ± 0.02 | 0.15 ± 0.02 | 0.18 ± 0.09 | 2 |
| 15 mM | 0.22 ± 0.02 | 0.21 ± 0.04 | 0.23 ± 0.03 | 0.30 ± 0.02 | 8 |
| 20 mM | 0.25 ± 0.03 | 0.11 ± 0.03 | 0.23 ± 0.03 | 0.28 ± 0.03 | 5 |
|  |  |  |  |  |  |
| **KA conc.** | **pre** | **Ctr. 1 Hz oLFS** | **post 1** | **post 2** | **n (sessions)** |
| 20 mM | 0.23 ± 0.03 | 0.25 ± 0.02 | - | - | 7 |
|  |  |  |  |  |  |
| **b Epileptic spike rate [Hz]** | | | | | |
| **KA conc.** | **pre** | **1 Hz oLFS** | **post 1** | **post 2** | **n (sessions)** |
| 10 mM | 0.30 ± 0.04 | 0.09 ± 0.02 | 0.47 ± 0.09 | 0.38 ± 0.10 | 6 |
| 15 mM | 0.80 ± 0.10 | 0.23 ± 0.07 | 0.67 ± 0.10 | 0.90 ± 0.14 | 12 |
| 20 mM | 0.84 ± 0.19 | 0.19 ± 0.04 | 0.60 ± 0.10 | 0.94 ± 0.20 | 7 |
|  |  |  |  |  |  |
| **KA conc.** | **pre** | **0.5 Hz oLFS** | **post 1** | **post 2** | **n (sessions)** |
| 10 mM | 0.51 ± 0.00 | 0.18 ± 0.00 | 0.14 ± 0.00 | 0.75 ± 0.00 | 1 |
| 15 mM | 0.91 ± 0.12 | 0.37 ± 0.09 | 0.69 ± 0.13 | 0.96 ± 0.13 | 8 |
| 20 mM | 0.81 ± 0.06 | 0.32 ± 0.03 | 0.57 ± 0.15 | 1.04 ± 0.19 | 4 |
|  |  |  |  |  |  |
| **KA conc.** | **pre** | **0.2 Hz oLFS** | **post 1** | **post 2** | **n (sessions)** |
| 10 mM | 0.35 ± 0.06 | 0.28 ± 0.04 | 0.48 ± 0.02 | 0.58 ± 0.16 | 4 |
| 15 mM | 0.96 ± 0.08 | 0.73 ± 0.14 | 0.89 ± 0.09 | 1.18 ± 0.10 | 8 |
| 20 mM | 0.94 ± 0.06 | 0.45 ± 0.10 | 0.94 ± 0.14 | 1.11 ± 0.18 | 5 |
|  |  |  |  |  |  |
| **KA conc.** | **pre** | **Ctr. 1 Hz oLFS** | **post 1** | **post 2** | **n (sessions)** |
| 20 mM | 0.85 ± 0.13 | 0.89 ± 0.09 | - | - | 7 |
|  |  |  |  |  |  |
| **c All KA animals pooled:** | | | | | |
| **Burst ratio** | | | | | |
| **oLFS frequency** | **pre** | **oLFS** | **post 1** | **post 2** | **n (sessions)** |
| 1 Hz | 0.16 ± 0.02 | 0.03 ± 0.01 | 0.15 ± 0.02 | 0.19 ± 0.03 | 25 |
| 0.5 Hz | 0.22 ± 0.01 | 0.16 ± 0.03 | 0.22 ± 0.02 | 0.29 ± 0.02 | 14 |
| 0.2 Hz | 0.22 ± 0.01 | 0.16 ± 0.03 | 0.22 ± 0.02 | 0.29 ± 0.02 | 15 |
|  |  |  |  |  |  |
| **d Epileptic spike rate [Hz]** | | | | | |
| **oLFS frequency** | **pre** | **oLFS** | **post 1** | **post 2** | **n (sessions)** |
| 1 Hz | 0.68 ± 0.08 | 0.19 ± 0.03 | 0.59 ± 0.06 | 0.79 ± 0.10 | 25 |
| 0.5 Hz | 0.85 ± 0.08 | 0.34 ± 0.06 | 0.61 ± 0.10 | 0.96 ± 0.09 | 13 |
| 0.2 Hz | 0.80 ± 0.08 | 0.54 ± 0.09 | 0.81 ± 0.07 | 1.02 ± 0. 10 | 17 |
| **e**  **Suppression efficacy [%]** | | | | | |
|  | **Burst ratio** | | **Epileptic spike rate [Hz]** | |  |
| **oLFS frequency** | **Median ± 95% CI** | **n (sessions)** | **Median ± 95% CI** | **n (sessions)** |  |
| 1 Hz | 86.79 ± [88.13, 100.00]% | 22 | 74.88 ± [68.41, 83.26]% | 25 |  |
| 0.5 Hz | 75.60 ± [53.52, 100.00]% | 16 | 61.90 ± [46.21, 76.70]% | 13 |  |
| 0.2 Hz | 40.15 ± [-3.48, 73.52]% | 14 | 40.20 ± [9.15, 51.67]% | 17 |  |

**Figure 4–Source Data 1: oLFS effect on ipsilateral epileptiform activity.** Burst ratios and epileptic spike rates of each sub‑session are listed for the three KA concentrations (10, 15, 20 mM) for each oLFS frequency (1, 0.5, 0.2 Hz) and no-virus controls (Ctr.). **a, b** The bust ratio and epileptic spike rate are reduced in all KA groups during oLFS but recover within the first hour of post-recording (post 1). This effect is also observed after 0.5 and 0.2 Hz oLFS but less pronounced. No change is observed in no-virus control animals. **c, d** Summary for all KA groups merged. **e** Median (± 95% CI) of the suppression efficacy for the three applied frequencies. oLFS at 1 Hz seems most effective for the suppression of epileptiform activity. Values are given as mean ± SEM. CI, confidence interval
