# Supplementary material for: Hippocampal low-frequency stimulation prevents seizure generation in a mouse model of mesial temporal lobe epilepsy
Source: eLife. 2020 Dec 22;9:e54518. doi: 10.7554/eLife.54518 (PMC7800381; doi:10.7554/eLife.54518)
Supplement: Figure 4—figure supplement 1—source data 1. — Burst ratios and epileptic spike rates of each sub-session are listed for the three KA concentrations (10, 15, 20 mM) for each oLFS frequency (1, 0.5, 0.2 Hz) and no-virus controls (Ctr.). (a, b) The bust ratio and epileptic spike rate are reduced in all KA groups during oLFS but recover within the first hour of post-recording (post 1). This effect is also observed after 0.5 and 0.2 Hz oLFS but less pronounced. No change is observed in no-virus control animals. (c, d) Summary for all KA groups merged. oLFS at 1 Hz seems most effective for the suppression of ictal and interictal activity in the contralateral dorsal hippocampus. (e) Median (±95% CI) of the suppression efficacy for the three applied frequencies. oLFS at 1 Hz seems most effective for the suppression of epileptiform activity. Values are given as mean ± SEM. [file elife-54518-fig4-figsupp1-data1.docx]

| **a Burst ratio** | | | | | |
| --- | --- | --- | --- | --- | --- |
| **KA conc.** | **pre** | **1 Hz oLFS** | **post 1** | **post 2** | **n (sessions)** |
| 10 mM | 0.09 ± 0.02 | 0.03 ± 0.02 | 0.06 ± 0.02 | 0.08 ± 0.03 | 6 |
| 15 mM | 0.15 ± 0.03 | 0.02 ± 0.01 | 0.13 ± 0.03 | 0.17 ± 0.05 | 9 |
| 20 mM | 0.07 ± 0.01 | 0.03 ± 0.02 | 0.10 ± 0.00 | 0.05 ± 0.03 | 3 |
|  |  |  |  |  |  |
| **KA conc.** | **pre** | **0.5 Hz oLFS** | **post 1** | **post 2** | **n (sessions)** |
| 10 mM | 0.07 ± 0.01 | 0.01 ± 0.01 | 0.07 ± 0.04 | 0.17 ± 0.04 | 2 |
| 15 mM | 0.16 ± 0.04 | 0.05 ± 0.02 | 0.07 ± 0.02 | 0.13 ± 0.04 | 10 |
| 20 mM | 0.09 ± 0.03 | 0.02 ± 0.01 | 0.10 ± 0.00 | 0.12 ± 0.06 | 2 |
|  |  |  |  |  |  |
| **KA conc.** | **pre** | **0.2 Hz oLFS** | **post 1** | **post 2** | **n (sessions)** |
| 10 mM | 0.10 ± 0.03 | 0.05 ± 0.01 | 0.09 ± 0.03 | 0.14 ± 0.03 | 4 |
| 15 mM | 0.14 ± 0.02 | 0.10 ± 0.03 | 0.12 ± 0.03 | 0.17 ± 0.04 | 9 |
| 20 mM | 0.10 ± 0.01 | 0.03 ± 0.02 | 0.10 ± 0.02 | 0.08 ± 0.02 | 4 |
|  |  |  |  |  |  |
| **KA conc.** | **pre** | **Ctr. 1 Hz oLFS** | **post 1** | **post 2** | **n (sessions)** |
| 20 mM | 0.23 ± 0.03 | 0.28 ± 0.01 | - | - | 4 |
|  |  |  |  |  |  |
| **b Epileptic spike rate [Hz]** | | | | | |
| **KA conc.** | **pre** | **1 Hz oLFS** | **post 1** | **post 2** | **n (sessions)** |
| 10 mM | 0.37 ± 0.08 | 0.08 ± 0.03 | 0.29 ± 0.04 | 0.32 ± 0.07 | 6 |
| 15 mM | 0.63 ± 0.10 | 0.10 ± 0.03 | 0.54 ± 0.08 | 0.64 ± 0.09 | 9 |
| 20 mM | 0.33 ± 0.07 | 0.10 ± 0.07 | 0.39 ± 0.11 | 0.33 ± 0.09 | 3 |
|  |  |  |  |  |  |
| **KA conc.** | **pre** | **0.5 Hz oLFS** | **post 1** | **post 2** | **n (sessions)** |
| 10 mM | 0.31 ± 0.12 | 0.07 ± 0.04 | 0.28 ± 0.14 | 0.60 ± 0.05 | 2 |
| 15 mM | 0.56 ± 0.13 | 0.20 ± 0.04 | 0.34 ± 0.08 | 0.53 ± 0.14 | 10 |
| 20 mM | 0.33 ± 0.09 | 0.14 ± 0.05 | 0.35 ± 0.04 | 0.45 ± 0.16 | 3 |
|  |  |  |  |  |  |
| **KA conc.** | **pre** | **0.2 Hz oLFS** | **post 1** | **post 2** | **n (sessions)** |
| 10 mM | 0.50 ± 0.20 | 0.27 ± 0.06 | 0.39 ± 0.13 | 0.48 ± 0.12 | 4 |
| 15 mM | 0.54 ± 0.09 | 0.37 ± 0.10 | 0.45 ± 0.09 | 0.57 ± 0.11 | 9 |
| 20 mM | 0.37 ± 0.04 | 0.19 ± 0.05 | 0.37 ± 0.07 | 0.29 ± 0.03 | 4 |
|  |  |  |  |  |  |
| **KA conc.** | **pre** | **Ctr. 1 Hz oLFS** | **post 1** | **post 2** | **n (sessions)** |
| 20 mM | 0.85 ± 0.17 | 0.98 ± 0.12 | - | - | 4 |
|  |  |  |  |  |  |
| **c All KA animals pooled:** | | | | | |
| Burst ratio | | | | | |
| **oLFS frequency** | **pre** | **oLFS** | **post 1** | **post 2** | **n (sessions)** |
| 1 Hz | 0.12 ± 0.02 | 0.04 ± 0.01 | 0.11 ± 0.02 | 0.13 ± 0.02 | 21 |
| 0.5 Hz | 0.14 ± 0.02 | 0.05 ± 0.01 | 0.09 ± 0.02 | 0.15 ± 0.03 | 19 |
| 0.2 Hz | 0.12 ± 0.01 | 0.07 ± 0.02 | 0.10 ± 0.02 | 0.1427 ± 0.02 | 21 |
|  |  |  |  |  |  |
| **d Epileptic spike rate [Hz]** | | | | | |
| **oLFS frequency** | **pre** | **oLFS** | **post 1** | **post 2** | **n (sessions)** |
| 1 Hz | 0.51 ± 0.06 | 0.10 ± 0.03 | 0.44 ± 0.05 | 0.49 ± 0.06 | 21 |
| 0.5 Hz | 0.47 ± 0.08 | 0.19 ± 0.03 | 0.35 ± 0.06 | 0.53 ± 0.08 | 19 |
| 0.2 Hz | 0.48 ± 0.05 | 0.29 ± 0.05 | 0.40 ± 0.05 | 0.45 ± 0.06 | 21 |
|  |  |  |  |  |  |
| **e**  **Suppression efficacy [%]** | | | | |  |
|  | **Burst ratio** | | **Epileptic spike rate [Hz]** | |  |
| **oLFS frequency** | **Median ± 95% CI** | **n (sessions)** | **Median ± 95% CI** | **n (sessions)** |  |
| 1 Hz | 88.65 ± [80.52, 100.00]% | 16 | 82.27 ± [71.29, 92.21]% | 18 |  |
| 0.5 Hz | 74.32 ± [53.05, 96.55] | 14 | 60.60 ± [43.52, 72.94]% | 14 |  |
| 0.2 Hz | 42.96 ± [7.83, 74.31] | 17 | 39.82 ± [8.02, 63.93]% | 17 |  |

**Figure 4–figure supplement 1–Source Data 1: oLFS effect on contralateral epileptiform activity.** Burst ratios and epileptic spike rates of each sub‑session are listed for the three KA concentrations (10, 15, 20 mM) for each oLFS frequency (1, 0.5, 0.2 Hz) and no-virus controls (Ctr.). **a, b** The bust ratio and epileptic spike rate are reduced in all KA groups during oLFS but recover within the first hour of post-recording (post 1). This effect is also observed after 0.5 and 0.2 Hz oLFS but less pronounced. No change is observed in no-virus control animals. **c, d** Summary for all KA groups merged. oLFS at 1 Hz seems most effective for the suppression of ictal and interictal activity in the contralateral dorsal hippocampus. **e** Median (± 95% CI) of the suppression efficacy for the three applied frequencies. oLFS at 1 Hz seems most effective for the suppression of epileptiform activity. Values are given as mean ± SEM. CI, confidence interval
