# Supplementary material for: Hippocampal low-frequency stimulation prevents seizure generation in a mouse model of mesial temporal lobe epilepsy
Source: eLife. 2020 Dec 22;9:e54518. doi: 10.7554/eLife.54518 (PMC7800381; doi:10.7554/eLife.54518)
Supplement: Figure 4—figure supplement 5—source data 1. — (a) Time spent running (>4 cm/s) of each sub-session is listed for the three KA concentrations (10, 15, 20 mM) for each oLFS frequency (1, 0.5, 0.2 Hz). The running behavior between KA groups is not significantly different. (b) During oLFS sessions, the movement is not impaired. The decrease in the movement over the 4 hr of LFP recording is most likely due to adaptation effects to the environment. Values are given as mean ± SEM. [file elife-54518-fig4-figsupp5-data1.docx]

| **A Movement [%]** | | | | | |
| --- | --- | --- | --- | --- | --- |
| **KA conc.** | **pre** | **1 Hz oLFS** | **post 1** | **post 2** | **n (sessions)** |
| 10 mM | 7.74 ± 1.78 | 6.43 ± 1.22 | 1.06 ± 0.64 | 4.05 ± 1.72 | 6 |
| 15 mM | 11.56 ± 2.59 | 9.24 ± 1.67 | 5.08 ± 1.64 | 3.01 ± 0.87 | 12 |
| 20 mM | **12.67 ± 4.53** | **7.84 ± 2.89** | **6.99 ± 2.94** | **3.16 ± 1.22** | 6 |
|  |  |  |  |  |  |
| **KA conc.** | **pre** | **0.5 Hz oLFS** | **post 1** | **post 2** | **n (sessions)** |
| 10 mM | **8.34 ± 2.12** | **8.40 ± 2.63** | **7.44 ± 2.25** | **2.58 ± 0.98** | 6 |
| 15 mM | **12.63 ± 3.07** | **6.52 ± 1.99** | **5.35 ± 1.68** | **2.28 ± 0.92** | 12 |
| 20 mM | **7.39 ± 2.31** | **6.19 ± 1.91** | **6.00 ± 1.60** | **2.59 ± 1.99** | 6 |
|  |  |  |  |  |  |
| **KA conc.** | **pre** | **0.2 Hz oLFS** | **post 1** | **post 2** | **n (sessions)** |
| 10 mM | **7.56 ± 2.77** | **5.11 ± 2.40** | **3.03 ± 1.38** | **5.28 ± 2.25** | 6 |
| 15 mM | **15.3 ± 2.13** | **5.52 ± 1.43** | **3.86 ± 1.67** | **3.50 ± 1.2** | 12 |
| 20 mM | **6.78 ± 2.19** | **6.91 ± 2.59** | **5.59 ± 1.79** | **5.43 ± 1.52** | 6 |
|  |  |  |  |  |  |
| **B All KA concentrations pooled:** | | | | | |
| **Movement [%]** | | | | | |
| **oLFS frequency** | **pre** | **oLFS** | **post 1** | **post 2** | **n (sessions)** |
| 1 Hz | **10.63 ± 1.68** | **8.17 ± 1.07** | **4.45 ± 1.13** | **3.31 ± 0.66** | 24 |
| 0.5 Hz | **10.74 ± 1.83** | **7.14 ± 1.32** | **6.03 ± 1.06** | **2.43 ± 0.70** | 24 |
| 0.2 Hz | **10.57 ± 1.57** | **5.78 ± 1.09** | **4.08 ± 0.99** | **4.43 ± 0.88** | 24 |

**Figure 4–figure supplement 5–Source Data 1: oLFS effect on running behavior over time. (A)** Time spent running (>4 cm/s) of each sub‑session is listed for the three KA concentrations (10, 15, 20 mM) for each oLFS frequency (1, 0.5, 0.2 Hz). The running behavior between KA groups is not significantly different. **(B)** During oLFS sessions, the movement is not impaired. The decrease in the movement over the four hours of LFP recording is most likely due to adaptation effects to the environment. Values are given as mean ± SEM.
