# Supplementary material for: Hippocampal low-frequency stimulation prevents seizure generation in a mouse model of mesial temporal lobe epilepsy
Source: eLife. 2020 Dec 22;9:e54518. doi: 10.7554/eLife.54518 (PMC7800381; doi:10.7554/eLife.54518)
Supplement: Figure 8—source data 1. — (A, B) High-load burst ratios and epileptic spike rates of each sub-session for the first and the second week of daily 1 Hz eLFS are listed. The high-load burst ratio and epileptic spike rate are reduced during eLFS but recover within the first hour of post-recording (post 1). (C) High-load burst ratios of reference LFP recordings (days 33 and 41 after SE) for 3 hr of individual animals. (D) High-load burst ratios for individual animals that went into 3-hr continuous eLFS experiments. Epileptiform activity is effectively reduced during ongoing eLFS but returns in a reduced manner within the first hour in some animals. The implant of EP167 broke after the last reference LFP recording and was therefore excluded. Values are given as mean ± SEM. [file elife-54518-fig8-data1.docx]

| **A** | **Week 1** | | | | | | |
| --- | --- | --- | --- | --- | --- | --- | --- |
|  | |  | **pre** | **1 Hz eLFS** | **post 1** | **post 2** | **n (animals)** |
| **Fig. 8C** | | **High-load burst ratio** | **0.18 ± 0.02** | **0.01 ± 0.00** | **0.10 ± 0.01** | **0.14 ± 0.02** | **7** |
| **Fig. 8D** | | **Epileptic spike rate [Hz]** | **0.68 ± 0.06** | **0.07 ± 0.01** | **0.45 ± 0.04** | **0.62 ± 0.07** | **7** |
|  | | | | | | | |
| **B** | **Week 2** | | | | | | |
|  | |  | **pre** | **1 Hz eLFS** | **post 1** | **post 2** | **n (animals)** |
| **Fig. 8F** | | **High-load burst ratio** | **0.20 ± 0.02** | **0.01 ± 0.00** | **0.12 ± 0.01** | **0.18 ± 0.01** | **7** |
| **Fig. 8G** | | **Epileptic spike rate [Hz]** | **0.72 ± 0.04** | **0.07 ± 0.02** | **0.52 ± 0.03** | **0.69 ± 0.03** | **7** |

| **C** | **Reference LFP: high-load burst ratio** | | | | | | | | |
| --- | --- | --- | --- | --- | --- | --- | --- | --- | --- |
| **Fig. 8H** | | **EP148** | **EP150** | **EP154** | **EP159** | **EP162** | **EP167** | **EP168** | **Row means** |
| **n (sessions)** | | **2** | **2** | **2** | **2** | **2** | **2** | **2** | **7** |
| **1st hour** | | **0.16** | **0.18** | **0.22** | **0.08** | **0.19** | **0.20** | **0.09** | **0.16** |
| **2nd hour** | | **0.25** | **0.19** | **0.18** | **0.18** | **0.27** | **0.22** | **0.20** | **0.21** |
| **3rd hour** | | **0.23** | **0.17** | **0.27** | **0.14** | **0.28** | **0.27** | **0.13** | **0.21** |
|  | | | | | | | | | |
| **D** | **3 hours continuous eLFS: high-load burst ratio** | | | | | | | | |
| **Fig. 8I** | | **EP148** | **EP150** | **EP154** | **EP159** | **EP162** | **EP167** | **EP168** | **Row means** |
| **n (sessions)** | | **2** | **2** | **2** | **2** | **2** | **0** | **2** | **6** |
| **pre** | | **0.19** | **0.19** | **0.15** | **0.11** | **0.16** | **x** | **0.25** | **0.17** |
| **1st hour eLFS** | | **0.00** | **0.01** | **0.00** | **0.01** | **0.01** | **x** | **0.00** | **0.01** |
| **2nd hour eLFS** | | **0.00** | **0.00** | **0.00** | **0.00** | **0.00** | **x** | **0.00** | **0.00** |
| **3rd hour eLFS** | | **0.00** | **0.00** | **0.00** | **0.00** | **0.00** | **x** | **0.00** | **0.00** |
| **post 1** | | **0.03** | **0.11** | **0.00** | **0.00** | **0.08** | **x** | **0.12** | **0.06** |

**Figure 8–Source Data 1: eLFS effect on ipsilateral epileptiform activity over time. (A, B) High-load burst ratios and epileptic spike rates of each sub‑session for the first and the second week of daily 1 Hz eLFS are listed. The high-load burst ratio and epileptic spike rate are reduced during eLFS but recover within the first hour of post-recording (post 1). (C) High-load burst ratios of reference LFP recordings (day 33 and 41 after SE) for three hours of individual animals. (D) High-load burst ratios for individual animals that went into three-hour continuous eLFS experiments. Epileptiform activity is effectively reduced during ongoing eLFS but returns in a reduced manner within the first hour in some animals. The implant of EP167 broke after the last reference LFP recording and was therefore excluded. Values are given as mean ± SEM.**
