# Supplementary material for: Hippocampal low-frequency stimulation prevents seizure generation in a mouse model of mesial temporal lobe epilepsy
Source: eLife. 2020 Dec 22;9:e54518. doi: 10.7554/eLife.54518 (PMC7800381; doi:10.7554/eLife.54518)
Supplement: Supplementary file 1. [file elife-54518-supp1.docx]

| **Parameter** | **Reference** | **Statistical test and summary of results** | | | |
| --- | --- | --- | --- | --- | --- |
|  |  |  |  |  |  |
|  |  |  |  |  |  |
| **GCD vol. [mm^3^]** | **Fig. 2B** | One-way ANOVA |  |  |  |
|  |  | P value | < 0.001 |  |  |
|  |  | Number of groups | 3 |  |  |
|  |  | Number of values per group | 3, 6, 5 |  |  |
|  |  | F value | 20.56 |  |  |
|  |  |  |  |  |  |
|  |  | Tukey's Multiple Comparison Test |  |  |  |
|  |  | Groups | Mean Diff. | 95% CI | P values |
|  |  | 10 mM KA vs. 15 mM KA | -1.319 | -1.885 to -0.7518 | < 0.001 |
|  |  | 10 mM KA vs. 20 mM KA | -1.081 | -1.670 to -0.4925 | < 0.001 |
|  |  | 15 mM KA vs. 20 mM KA | 0.2371 | -0.2946 to 0.7688 | > 0.05 |
|  |  |  |  |  |  |
| **CA1 cell loss [%]** | **Fig. 2C** | One-way ANOVA |  |  |  |
|  |  | P value | ns |  |  |
|  |  | Number of groups | 3 |  |  |
|  |  | Number of values per group | 3, 6, 5 |  |  |
|  |  | F value | 1.029 |  |  |
|  |  |  |  |  |  |
|  |  | Tukey's Multiple Comparison Test |  |  |  |
|  |  | Groups | Mean Diff. | 95% CI | P values |
|  |  | 10 mM KA vs. 15 mM KA | -11.85 | -33.98 to 10.27 | > 0.05 |
|  |  | 10 mM KA vs. 20 mM KA | -6.219 | -29.21 to 16.78 | > 0.05 |
|  |  | 15 mM KA vs. 20 mM KA | 5.634 | -15.12 to 26.39 | > 0.05 |
|  |  |  |  |  |  |
| **Loss of** | **Fig. 2D** | One-way ANOVA |  |  |  |
| **NeuN+ hilar cells [%]** |  | P value | < 0.001 |  |  |
|  |  | Number of groups | 3 |  |  |
|  |  | Number of values per group | 3, 6, 5 |  |  |
|  |  | F value | 18.59 |  |  |
|  |  |  |  |  |  |
|  |  | Tukey's Multiple Comparison Test |  |  |  |
|  |  | Groups | Mean Diff. | 95% CI | P values |
|  |  | 10 mM KA vs. 15 mM KA | -33.7 | -50.48 to -16.91 | < 0.001 |
|  |  | 10 mM KA vs. 20 mM KA | -35.76 | -53.20 to -18.32 | < 0.001 |
|  |  | 15 mM KA vs. 20 mM KA | -2.06 | -17.80 to 13.68 | > 0.05 |
|  |  |  |  |  |  |
| **Loss of** | **Fig. 2E** | One-way ANOVA |  |  |  |
| **Gad67+ hilar cells [%]** |  | P value | ns |  |  |
|  |  | Number of groups | 3 |  |  |
|  |  | Number of values per group | 3, 3, 5 |  |  |
|  |  | F value | 2.645 |  |  |
|  |  |  |  |  |  |
|  |  | Tukey's Multiple Comparison Test |  |  |  |
|  |  | Groups | Mean Diff. | 95% CI | P values |
|  |  | 10 mM KA vs. 15 mM KA | -15.01 | -33.72 to 3.688 | > 0.05 |
|  |  | 10 mM KA vs. 20 mM KA | -9.086 | -25.51 to 7.340 | > 0.05 |
|  |  | 15 mM KA vs. 20 mM KA | 5.929 | -11.95 to 23.81 | > 0.05 |
|  |  |  |  |  |  |
| **High-load burst ratio** | **Fig. 3D** | One-way ANOVA |  |  |  |
| **(idHC)** |  | P value | < 0.01 |  |  |
|  |  | Number of groups | 3 |  |  |
|  |  | Number of values per group | 3, 6, 5 |  |  |
|  |  | F value | 9.613 |  |  |
|  |  |  |  |  |  |
|  |  | Tukey's Multiple Comparison Test |  |  |  |
|  |  | Groups | Mean Diff. | 95% CI | P values |
|  |  | 10 mM KA vs. 15 mM KA | -0.1239 | -0.2119 to -0.03596 | < 0.01 |
|  |  | 10 mM KA vs. 20 mM KA | -0.1376 | -0.2290 to -0.04614 | < 0.01 |
|  |  | 15 mM KA vs. 20 mM KA | -0.01363 | -0.09617 to 0.06890 | > 0.05 |
|  |  |  |  |  |  |
| **High-load burst ratio** | **Fig. 3E** | One-way ANOVA |  |  |  |
| **(cdHC)** |  | P value | ns |  |  |
|  |  | Number of groups | 3 |  |  |
|  |  | Number of values per group | 3, 6, 5 |  |  |
|  |  | F value | 1.276 |  |  |
|  |  |  |  |  |  |
|  |  | Tukey's Multiple Comparison Test |  |  |  |
|  |  | Groups | Mean Diff. | 95% CI | P values |
|  |  | 10 mM KA vs. 15 mM KA | -0.05136 | -0.1439 to 0.04120 | > 0.05 |
|  |  | 10 mM KA vs. 20 mM KA | -0.01311 | -0.1093 to 0.08308 | > 0.05 |
|  |  | 15 mM KA vs. 20 mM KA | 0.03824 | -0.04859 to 0.1251 | > 0.05 |
|  |  |  |  |  |  |
| **Epileptic spike rate [Hz]** | **Fig. 3F** | One-way ANOVA |  |  |  |
| **(idHC)** |  | P value | < 0.01 |  |  |
|  |  | Number of groups | 3 |  |  |
|  |  | Number of values per group | 3, 6, 5 |  |  |
|  |  | F value | 7.08 |  |  |
|  |  |  |  |  |  |
|  |  | Tukey's Multiple Comparison Test |  |  |  |
|  |  | Groups | Mean Diff. | 95% CI | P values |
|  |  | 10 mM KA vs. 15 mM KA | -0.4354 | -0.7810 to -0.08976 | < 0.05 |
|  |  | 10 mM KA vs. 20 mM KA | -0.4473 | -0.8065 to -0.08813 | < 0.05 |
|  |  | 15 mM KA vs. 20 mM KA | -0.01193 | -0.3362 to 0.3123 | > 0.05 |
|  |  |  |  |  |  |
|  |  | One-way ANOVA |  |  |  |
| **Epileptic spike rate [Hz]** | **Fig. 3G** | P value | ns |  |  |
| **(cdHC)** |  | Number of groups | 3 |  |  |
|  |  | Number of values per group | 3, 6, 5 |  |  |
|  |  | F value | 1.241 |  |  |
|  |  |  |  |  |  |
|  |  | Tukey's Multiple Comparison Test |  |  |  |
|  |  | Groups | Mean Diff. | 95% CI | P values |
|  |  | 10 mM KA vs. 15 mM KA | -0.1552 | -0.4399 to 0.1296 | > 0.05 |
|  |  | 10 mM KA vs. 20 mM KA | -0.03818 | -0.3341 to 0.2577 | > 0.05 |
|  |  | 15 mM KA vs. 20 mM KA | 0.117 | -0.1501 to 0.3841 | > 0.05 |
|  |  |  |  |  |  |
| **High-load burst ratio (idHC)** | **Fig. 3H** | Pearson's correlation |  |  |  |
| **vs.** |  | Number of XY pairs | 15 |  |  |
| **GCD vol. [mm^3^]** |  |  |  |  |  |
|  |  | Slope | 95% CI | R² value | P value |
|  |  | 0.101 ± 0.0181 | 0.5750 to 0.9454 | 0.7054 | < 0.0001 |
|  |  |  |  |  |  |
| **High-load burst ratio (idHC)** | **Fig. 3I** | Pearson's correlation |  |  |  |
| **vs.** |  | Number of XY pairs | 16 |  |  |
| **Loss of NeuN+ hilar cells [%]** |  |  |  |  |  |
|  |  | Slope | 95% CI | R² value | P value |
|  |  | 0.00265 ± 0.000861 | 0.5750 to 0.9454 | 0.4037 | < 0.01 |
|  |  |  |  |  |  |
| **High-load burst ratio (idHC)** | **Fig. 4E** | One-way ANOVA |  |  |  |
|  |  | P value | < 0.0001 |  |  |
|  |  | Number of groups | 4 |  |  |
|  |  | Number of values per group | 25 |  |  |
|  |  | F value | 21.06 |  |  |
|  |  |  |  |  |  |
|  |  | Tukey's Multiple Comparison Test |  |  |  |
|  |  | Groups | Mean Diff. | 95% CI | P values |
|  |  | pre vs. 1 Hz oLFS | 0.1261 | 0.07125 to 0.1809 | < 0.0001 |
|  |  | pre vs. post 1 | 0.00784 | -0.05189 to 0.06757 | > 0.05 |
|  |  | pre vs. post 2 | -0.03408 | -0.09085 to 0.02269 | > 0.05 |
|  |  | 1 Hz oLFS vs. post 1 | -0.1182 | -0.1666 to -0.06987 | < 0.0001 |
|  |  | 1 Hz oLFS vs. post 2 | -0.1602 | -0.2298 to -0.09048 | < 0.0001 |
|  |  | post 1 vs. post 2 | -0.04192 | -0.1063 to 0.02248 | > 0.05 |
|  |  |  |  |  |  |
| **Epileptic spike rate [Hz]** | **Fig. 4F** | One-way ANOVA |  |  |  |
| **(idHC)** |  | P value | < 0.0001 |  |  |
|  |  | Number of groups | 4 |  |  |
|  |  | Number of values per group | 25 |  |  |
|  |  | F value | 26.81 |  |  |
|  |  |  |  |  |  |
|  |  | Tukey's Multiple Comparison Test |  |  |  |
|  |  | Groups | Mean Diff. | 95% CI | P values |
|  |  | pre vs. 1 Hz oLFS | 0.495 | 0.3004 to 0.6896 | < 0.0001 |
|  |  | pre vs. post 1 | 0.0958 | -0.1122 to 0.3038 | > 0.05 |
|  |  | pre vs. post 2 | -0.1046 | -0.2734 to 0.06413 | > 0.05 |
|  |  | 1 Hz oLFS vs. post 1 | -0.3992 | -0.5149 to -0.2835 | < 0.0001 |
|  |  | 1 Hz oLFS vs. post 2 | -0.5996 | -0.8459 to -0.3533 | < 0.0001 |
|  |  | post 1 vs. post 2 | -0.2004 | -0.4247 to 0.02382 | > 0.05 |
|  |  |  |  |  |  |
| **High-load burst ratio** | **Fig. 4H** | Mixed-effects model (REML) |  |  |  |
| **(idHC)** |  | P value | < 0.0001 |  |  |
|  |  | Number of groups | 4 |  |  |
|  |  | Number of values per group | 14, 13, 14, 14 |  |  |
|  |  | Chi-square, df | 9.277, 1 |  |  |
|  |  |  |  |  |  |
|  |  | Tukey's Multiple Comparison Test |  |  |  |
|  |  | Groups | Mean Diff. | 95% CI | P values |
|  |  | pre vs. 0.5 Hz oLFS | 0.1457 | 0,07656 to 0,2148 | < 0.001 |
|  |  | pre vs. post 1 | 0.0735 | -0,002375 to 0,1494 | > 0.05 |
|  |  | pre vs. post 2 | -0.05057 | -0,1352 to 0,03410 | > 0.05 |
|  |  | 0.5 Hz oLFS vs. post 1 | -0.07216 | -0,1342 to -0,01016 | < 0.05 |
|  |  | 0.5 Hz oLFS vs. post 2 | -0.1962 | -0,2663 to -0,1262 | < 0.0001 |
|  |  | post 1 vs. post 2 | -0.1241 | -0,2071 to -0,04100 | < 0.01 |
|  |  |  |  |  |  |
| **Epileptic spike rate [Hz]** | **Fig. 4I** | One-way ANOVA |  |  |  |
| **(idHC)** |  | P value | < 0.0001 |  |  |
|  |  | Number of groups | 4 |  |  |
|  |  | Number of values per group | 13 |  |  |
|  |  | F value | 28.38 |  |  |
|  |  |  |  |  |  |
|  |  | Tukey's Multiple Comparison Test |  |  |  |
|  |  | Groups | Mean Diff. | 95% CI | P values |
|  |  | pre vs. 0.5 Hz oLFS | 0.5109 | 0.3550 to 0.6669 | < 0.0001 |
|  |  | pre vs. post 1 | 0.2433 | 0.01003 to 0.4766 | < 0.05 |
|  |  | pre vs. post 2 | -0.1147 | -0.3702 to 0.1407 | > 0.05 |
|  |  | 0.5 Hz oLFS vs. post 1 | -0.2676 | -0.4795 to -0.05568 | < 0.05 |
|  |  | 0.5 Hz oLFS vs. post 2 | -0.6257 | -0.8077 to -0.4437 | < 0.0001 |
|  |  | post 1 vs. post 2 | -0.3581 | -0.6134 to -0.1027 | < 0.01 |
|  |  |  |  |  |  |
| **High-load burst ratio** | **Fig. 4K** | Mixed-effects model (REML) |  |  |  |
| **(idHC)** |  | P value | < 0.0001 |  |  |
|  |  | Number of groups | 4 |  |  |
|  |  | Number of values per group | 15, 15, 15, 14 |  |  |
|  |  | Chi-square, df | 8.396, 1 |  |  |
|  |  |  |  |  |  |
|  |  | Tukey's Multiple Comparison Test |  |  |  |
|  |  | Groups | Mean Diff. | 95% CI | P values |
|  |  | pre vs. 0.2 Hz oLFS | 0.06387 | -0,01437 to 0,1421 | > 0.05 |
|  |  | pre vs. post 1 | 0.007667 | -0,04530 to 0,06063 | > 0.05 |
|  |  | pre vs. post 2 | -0.0698 | -0,1275 to -0,01206 | < 0.05 |
|  |  | 0.2 Hz oLFS vs. post 1 | -0.0562 | -0,1212 to 0,008788 | > 0.05 |
|  |  | 0.2 Hz oLFS vs. post 2 | -0.1337 | -0,2115 to -0,05583 | < 0.01 |
|  |  | post 1 vs. post 2 | -0.07747 | -0,1405 to -0,01446 | < 0.05 |
|  |  |  |  |  |  |
| **Epileptic spike rate [Hz]** | **Fig. 4L** | One-way ANOVA |  |  |  |
| **(idHC)** |  | P value | < 0.0001 |  |  |
|  |  | Number of groups | 4 |  |  |
|  |  | Number of values per group | 14 |  |  |
|  |  | F value | 19.84 |  |  |
|  |  |  |  |  |  |
|  |  | Tukey's Multiple Comparison Test |  |  |  |
|  |  | Groups | Mean Diff. | 95% CI | P values |
|  |  | pre vs. 0.2 Hz oLFS | 0.3175 | 0.09316 to 0.4400 | < 0.01 |
|  |  | pre vs. post 1 | 0.03667 | -0.1428 to 0.1396 | > 0.05 |
|  |  | pre vs. post 2 | -0.226 | -0.3630 to -0.05486 | < 0.01 |
|  |  | 0.2 Hz oLFS vs. post 1 | -0.2808 | -0.4440 to -0.09225 | < 0.01 |
|  |  | 0.2 Hz oLFS vs. post 2 | -0.5435 | -0.7038 to -0.2472 | < 0.001 |
|  |  | post 1 vs. post 2 | -0.2626 | -0.4124 to -0.002262 | < 0.05 |
|  |  |  |  |  |  |
| **High-load burst ratio (idHC)** | **Fig. 4N** | Paired Student's t test. two-tailed |  |  |  |
| **no virus control** |  | Number of pairs (Pre vs. 1 Hz oLFS) | 7 |  |  |
|  |  |  |  |  |  |
|  |  | Difference ± SEM | 95% CI | R² value | P value |
|  |  | -0.01629 ± 0.02275 | -0.07195 to 0.03938 | 0.07869 | > 0.05 |
|  |  |  |  |  |  |
|  |  |  |  |  |  |
| **Epileptic spike rate [Hz]** | **Fig. 4O** | Paired Student's t test. two-tailed |  |  |  |
| **(idHC) no virus control** |  | Number of pairs (Pre vs. 1 Hz oLFS) | 7 |  |  |
|  |  |  |  |  |  |
|  |  | Difference ± SEM | 95% CI | R² value | P value |
|  |  | -0.03800 ± 0.09214 | -0.2635 to 0.1875 | 0.02756 | > 0.05 |
|  |  |  |  |  |  |
|  |  |  |  |  |  |
| **Stimulation efficacy [%]** | **Fig. 4P** | Kruskal-Wallis test ANOVA |  |  |  |
| **High-load burst ratio (idHC)** |  | P value | < 0.01 |  |  |
|  |  | Number of groups | 3 |  |  |
|  |  | Number of values per group | 22, 16, 14 |  |  |
|  |  | Kruskal-Wallis statistic | 12.83 |  |  |
|  |  |  |  |  |  |
|  |  | Dunn's multiple comparisons test |  |  |  |
|  |  | Groups | Mean Diff. |  | P values |
|  |  | 1 Hz oLFS vs. 0.5 Hz oLFS | 4.963 |  | > 0.05 |
|  |  | 1 Hz oLFS vs. 0.2 Hz oLFS | 18.22 |  | < 0.01 |
|  |  | 0.5 Hz oLFS vs. 0.2 Hz oLFS | 13.25 |  | < 0.05 |
|  |  |  |  |  |  |
| **Stimulation efficacy [%]** | **Fig. 4Q** | Kruskal-Wallis test ANOVA |  |  |  |
| **Epileptic spike rate (idHC)** |  | P value | < 0.001 |  |  |
|  |  | Number of groups | 3 |  |  |
|  |  | Number of values per group | 25, 13, 17 |  |  |
|  |  | Kruskal-Wallis statistic | 17.93 |  |  |
|  |  |  |  |  |  |
|  |  | Dunn's multiple comparisons test |  |  |  |
|  |  | Groups | Mean Diff. |  | P values |
|  |  | 1 Hz oLFS vs. 0.5 Hz oLFS | 7.203 |  | > 0.05 |
|  |  | 1 Hz oLFS vs. 0.2 Hz oLFS | 21.28 |  | < 0.001 |
|  |  | 0.5 Hz oLFS vs. 0.2 Hz oLFS | 14.08 |  | > 0.05 |
|  |  |  |  |  |  |
| **High-load burst ratio** | **Fig4, S1E** | Mixed-effects model (REML) |  |  |  |
| **(cdHC)** |  | P value | < 0.0001 |  |  |
|  |  | Number of groups | 4 |  |  |
|  |  | Number of values per group | 21, 21, 20, 20 |  |  |
|  |  | Chi-square, df | 11.85, 1 |  |  |
|  |  |  |  |  |  |
|  |  | Tukey's Multiple Comparison Test |  |  |  |
|  |  | Groups | Mean Diff. | 95% CI | P values |
|  |  | pre vs. 1 Hz oLFS | 0.09001 | 0.03845 to 0.1416 | < 0.001 |
|  |  | pre vs. post 1 | 0.01875 | -0.02650 to 0.06399 | > 0.05 |
|  |  | pre vs. post 2 | -0.002163 | -0.04509 to 0.04076 | > 0.05 |
|  |  | 1 Hz oLFS vs. post 1 | -0.07126 | -0.1199 to -0.02261 | < 0.01 |
|  |  | 1 Hz oLFS vs. post 2 | -0.09217 | -0.1563 to -0.02799 | < 0.01 |
|  |  | post 1 vs. post 2 | -0.02091 | -0.06270 to 0.02088 | > 0.05 |
|  |  |  |  |  |  |
| **Epileptic spike rate [Hz]** | **Fig4, S1F** | Mixed-effects model (REML) |  |  |  |
| **(cdHC)** |  | P value | < 0.0001 |  |  |
|  |  | Number of groups | 4 |  |  |
|  |  | Number of values per group | 21, 21, 20, 20 |  |  |
|  |  | Chi-square, df | 21.54, 1 |  |  |
|  |  |  |  |  |  |
|  |  | Tukey's Multiple Comparison Test |  |  |  |
|  |  | Groups | Mean Diff. | 95% CI | P values |
|  |  | pre vs. 1 Hz oLFS | 0.3791 | 0.2217 to 0.5365 | < 0.0001 |
|  |  | pre vs. post 1 | 0.06507 | -0.04252 to 0.1727 | > 0.05 |
|  |  | pre vs. post 2 | 0.01966 | -0.09284 to 0.1322 | > 0.05 |
|  |  | 1 Hz oLFS vs. post 1 | -0.314 | -0.4522 to -0.1759 | < 0.0001 |
|  |  | 1 Hz oLFS vs. post 2 | -0.3594 | -0.5394 to -0.1795 | < 0.001 |
|  |  | post 1 vs. post 2 | -0.04541 | -0.1480 to 0.05722 | > 0.05 |
|  |  |  |  |  |  |
| **High-load burst ratio** | **Fig4, S1H** | Mixed-effects model (REML) |  |  |  |
| **(cdHC)** |  | P value | < 0.0001 |  |  |
|  |  | Number of groups | 4 |  |  |
|  |  | Number of values per group | 19, 18, 19, 19 |  |  |
|  |  | Chi-square, df | 31.66, 1 |  |  |
|  |  |  |  |  |  |
|  |  | Tukey's Multiple Comparison Test |  |  |  |
|  |  | Groups | Mean Diff. | 95% CI | P values |
|  |  | pre vs. 0.5 Hz oLFS | 0.08974 | 0.03959 to 0.1399 | < 0.001 |
|  |  | pre vs. post 1 | 0.05065 | 0.003552 to 0.09776 | < 0.05 |
|  |  | pre vs. post 2 | -0.01694 | -0.06432 to 0.03045 | > 0.05 |
|  |  | 0.5 Hz oLFS vs. post 1 | -0.03908 | -0.07717 to -0.0009986 | < 0.05 |
|  |  | 0.5 Hz oLFS vs. post 2 | -0.1067 | -0.1698 to -0.04353 | < 0.001 |
|  |  | post 1 vs. post 2 | -0.06759 | -0.1214 to -0.01381 | < 0.05 |
|  |  |  |  |  |  |
| **Epileptic spike rate [Hz]** | **Fig4, S1I** | Mixed-effects model (REML) |  |  |  |
| **(cdHC)** |  | P value | < 0.0001 |  |  |
|  |  | Number of groups | 4 |  |  |
|  |  | Number of values per group | 19, 18, 19, 19 |  |  |
|  |  | Chi-square, df | 35,76, 1 |  |  |
|  |  |  |  |  |  |
|  |  | Tukey's Multiple Comparison Test |  |  |  |
|  |  | Groups | Mean Diff. | 95% CI | P values |
|  |  | pre vs. 0.5 Hz oLFS | 0.2907 | 0.1080 to 0.4734 | < 0.01 |
|  |  | pre vs. post 1 | 0.1252 | -0.03037 to 0.2808 | > 0.05 |
|  |  | pre vs. post 2 | -0.04593 | -0.1495 to 0.05763 | > 0.05 |
|  |  | 0.5 Hz oLFS vs. post 1 | -0.1655 | -0.2634 to -0.06755 | < 0.001 |
|  |  | 0.5 Hz oLFS vs. post 2 | -0.3366 | -0.5275 to -0.1457 | < 0.001 |
|  |  | post 1 vs. post 2 | -0.1711 | -0.3274 to -0.01483 | < 0.05 |
|  |  |  |  |  |  |
| **High-load burst ratio** | **Fig4, S1K** | One-way ANOVA |  |  |  |
| **(cdHC)** |  | P value | < 0.0001 |  |  |
|  |  | Number of groups | 4 |  |  |
|  |  | Number of values per group | 21 |  |  |
|  |  | F value | 9.182 |  |  |
|  |  |  |  |  |  |
|  |  | Tukey's Multiple Comparison Test |  |  |  |
|  |  | Groups | Mean Diff. | 95% CI | P values |
|  |  | pre vs. 0.2 Hz oLFS | 0.05068 | 0.01546 to 0.08589 | < 0.01 |
|  |  | pre vs. post 1 | 0.01475 | -0.01375 to 0.04324 | > 0.05 |
|  |  | pre vs. post 2 | -0.01499 | -0.05458 to 0.02460 | > 0.05 |
|  |  | 0.2 Hz oLFS vs. post 1 | -0.03593 | -0.07076 to -0.001100 | < 0.05 |
|  |  | 0.2 Hz oLFS vs. post 2 | -0.06566 | -0.1065 to -0.02485 | < 0.01 |
|  |  | post 1 vs. post 2 | -0.02973 | -0.06979 to 0.01033 | > 0.05 |
|  |  |  |  |  |  |
| **Epileptic spike rate [Hz]** | **Fig4, S1L** | One-way ANOVA |  |  |  |
| **(cdHC)** |  | P value | < 0.0001 |  |  |
|  |  | Number of groups | 4 |  |  |
|  |  | Number of values per group | 21 |  |  |
|  |  | F value | 11.77 |  |  |
|  |  |  |  |  |  |
|  |  | Tukey's Multiple Comparison Test |  |  |  |
|  |  | Groups | Mean Diff. | 95% CI | P values |
|  |  | pre vs. 0.2 Hz oLFS | 0.1892 | 0.08235 to 0.2961 | < 0.001 |
|  |  | pre vs. post 1 | 0.0729 | -0.01467 to 0.1605 | > 0.05 |
|  |  | pre vs. post 2 | 0.02867 | -0.05602 to 0.1133 | > 0.05 |
|  |  | 0.2 Hz oLFS vs. post 1 | -0.1163 | -0.2099 to -0.02273 | < 0.05 |
|  |  | 0.2 Hz oLFS vs. post 2 | -0.1606 | -0.2612 to -0.05994 | < 0.01 |
|  |  | post 1 vs. post 2 | -0.04423 | -0.1455 to 0.05706 | > 0.05 |
|  |  |  |  |  |  |
| **High-load burst ratio (cdHC)** | **Fig4, S1N** | Paired Student's t test. two-tailed |  |  |  |
| **no virus control** |  | Number of pairs (Pre vs. 1 Hz oLFS) | 4 |  |  |
|  |  |  |  |  |  |
|  |  | Difference ± SEM | 95% CI | R² value | P value |
|  |  | -0.01629 ± 0.02275 | -0.08180 to 0.04041 | 0.2791 | > 0.05 |
|  |  |  |  |  |  |
|  |  |  |  |  |  |
| **Epileptic spike rate [Hz]** | **Fig4, S1O** | Paired Student's t test. two-tailed |  |  |  |
| **(cdHC) no virus control** |  | Number of pairs (Pre vs. 1 Hz oLFS) | 4 |  |  |
|  |  |  |  |  |  |
|  |  | Difference ± SEM | 95% CI | R² value | P value |
|  |  | 0.1132 ± 0.05680 | -0.06754 to 0.2940 | 0.5698 | > 0.05 |
|  |  |  |  |  |  |
|  |  |  |  |  |  |
| **Stimulation efficacy [%]** | **Fig4, S1P** | Kruskal-Wallis test ANOVA |  |  |  |
| **High-load burst ratio (cdHC)** |  | P value | < 0.01 |  |  |
|  |  | Number of groups | 3 |  |  |
|  |  | Number of values per group | 16, 14, 17 |  |  |
|  |  | Kruskal-Wallis statistic | 9.521 |  |  |
|  |  |  |  |  |  |
|  |  | Dunn's multiple comparisons test |  |  |  |
|  |  | Groups | Mean Diff. |  | P values |
|  |  | 1 Hz oLFS vs. 0.5 Hz oLFS | 7.768 |  | > 0.05 |
|  |  | 1 Hz oLFS vs. 0.2 Hz oLFS | 14.68 |  | < 0.01 |
|  |  | 0.5 Hz oLFS vs. 0.2 Hz oLFS | 6.916 |  | > 0.05 |
|  |  |  |  |  |  |
| **Stimulation efficacy [%]** | **Fig4, S1Q** | Kruskal-Wallis test ANOVA |  |  |  |
| **Epileptic spike rate (cdHC)** |  | P value | < 0.001 |  |  |
|  |  | Number of groups | 3 |  |  |
|  |  | Number of values per group | 18, 14, 17 |  |  |
|  |  | Kruskal-Wallis statistic | 17.49 |  |  |
|  |  |  |  |  |  |
|  |  | Dunn's multiple comparisons test |  |  |  |
|  |  | Groups | Mean Diff. |  | P values |
|  |  | 1 Hz oLFS vs. 0.5 Hz oLFS | 11.76 |  | > 0.05 |
|  |  | 1 Hz oLFS vs. 0.2 Hz oLFS | 20.10 |  | < 0.0001 |
|  |  | 0.5 Hz oLFS vs. 0.2 Hz oLFS | 8.336 |  | > 0.05 |
|  |  |  |  |  |  |
| **Movement [%]** | **Fig4, S5A** | Mixed-effects model (REML) |  |  |  |
|  |  | P value | < 0.0001 |  |  |
|  |  | Number of groups | 4 |  |  |
|  |  | Number of values per group | 21, 21, 23, 24 |  |  |
|  |  | Chi-square, df | 4.114, 1 |  |  |
|  |  |  |  |  |  |
|  |  | Tukey's Multiple Comparison Test |  |  |  |
|  |  | Groups | Mean Diff. | 95% CI | P values |
|  |  | pre vs. 1 Hz oLFS | 2.458 | -2.828 to 7.745 | > 0.05 |
|  |  | pre vs. post 1 | 6.179 | 1.359 to 11.00 | < 0.01 |
|  |  | pre vs. post 2 | 7.318 | 2.704 to 11.93 | < 0.01 |
|  |  | 1 Hz oLFS vs. post 1 | 3.721 | 0.2643 to 7.177 | < 0.05 |
|  |  | 1 Hz oLFS vs. post 2 | 4.86 | 1.354 to 8.366 | < 0.01 |
|  |  | post 1 vs. post 2 | 1.139 | -2.038 to 4.315 | > 0.05 |
|  |  |  |  |  |  |
| **Movement [%]** | **Fig4, S5B** | Mixed-effects model (REML) |  |  |  |
|  |  | P value | < 0.001 |  |  |
|  |  | Number of groups | 4 |  |  |
|  |  | Number of values per group | 22, 22, 24, 23 |  |  |
|  |  | Chi-square, df | 1.546, 1 |  |  |
|  |  |  |  |  |  |
|  |  | Tukey's Multiple Comparison Test |  |  |  |
|  |  | Groups | Mean Diff. | 95% CI | P values |
|  |  | pre vs. 0.5 Hz oLFS | 3.601 | -2.151 to 9.352 | > 0.05 |
|  |  | pre vs. post 1 | 4.708 | -1.045 to 10.46 | > 0.05 |
|  |  | pre vs. post 2 | 8.315 | 3.220 to 13.41 | < 0.001 |
|  |  | 0.5 Hz oLFS vs. post 1 | 1.107 | -2.825 to 5.039 | > 0.05 |
|  |  | 0.5 Hz oLFS vs. post 2 | 4.714 | 0.2042 to 9.224 | < 0.05 |
|  |  | post 1 vs. post 2 | 3.607 | 0.4556 to 6.758 | < 0.05 |
|  |  |  |  |  |  |
| **Movement [%]** | **Fig4, S5C** | Mixed-effects model (REML) |  |  |  |
|  |  | P value | < 0.001 |  |  |
|  |  | Number of groups | 4 |  |  |
|  |  | Number of values per group | 22, 23, 24, 24 |  |  |
|  |  | Chi-square, df | 6.973, 1 |  |  |
|  |  |  |  |  |  |
|  |  | Tukey's Multiple Comparison Test |  |  |  |
|  |  | Groups | Mean Diff. | 95% CI | P values |
|  |  | pre vs. 0.2 Hz oLFS | 4.795 | 0.2997 to 9.291 | < 0.05 |
|  |  | pre vs. post 1 | 6.491 | 1.729 to 11.25 | < 0.01 |
|  |  | pre vs. post 2 | 6.147 | 1.657 to 10.64 | < 0.01 |
|  |  | 0.2 Hz oLFS vs. post 1 | 1.696 | -2.164 to 5.555 | > 0.05 |
|  |  | 0.2 Hz oLFS vs. post 2 | 1.352 | -0.9734 to 3.677 | > 0.05 |
|  |  | post 1 vs. post 2 | -0.3439 | -3.545 to 2.857 | > 0.05 |
|  |  |  |  |  |  |
| **Seizure induction time [s]** | **Fig. 5B** | One-way ANOVA |  |  |  |
|  |  | P value | < 0.01 |  |  |
|  |  | Number of groups | 3 |  |  |
|  |  | Number of animals per group | 4, 6, 3 |  |  |
|  |  | F value | 11.71 |  |  |
|  |  |  |  |  |  |
|  |  | Tukey's Multiple Comparison Test |  |  |  |
|  |  | Groups | Mean Diff. | 95% CI | P values |
|  |  | 10 mM KA vs. 15 mM KA | -2.083 | -5.970 to 1.804 | > 0.05 |
|  |  | 10 mM KA vs. 20 mM KA | -7.917 | -12.52 to -3.318 | < 0.01 |
|  |  | 15 mM KA vs. 20 mM KA | -5.833 | -10.09 to -1.575 | < 0.01 |
|  |  |  |  |  |  |
| **Seizure probability [%]** | **Fig. 5E** | Wilcoxon matched-pairs signed rank test |  |  |  |
|  |  | Number of pairs (w/o oLFS vs. w/ 1Hz pre-oLFS) | 13 |  |  |
|  |  |  |  |  |  |
|  |  | Median of differences | Sum of positive, negative ranks |  | P value |
|  |  | -100 | 0 , -78 |  | < 0.001 |
|  |  |  |  |  |  |
|  |  |  |  |  |  |
| **Mean Racine scale** | **Fig. 5F** | Wilcoxon matched-pairs signed rank test |  |  |  |
|  |  | Number of pairs (w/o oLFS vs. w/ 1Hz pre-oLFS) | 13 |  |  |
|  |  |  |  |  |  |
|  |  | Median of differences | Sum of positive, negative ranks |  | P value |
|  |  | -2.33 | 1 , -90 |  | < 0.001 |
|  |  |  |  |  |  |
|  |  |  |  |  |  |
| **AUC (10 Hz) [au]** | **Fig. 5G** | Paired Student's t test. two-tailed |  |  |  |
|  |  | Number of pairs (w/o oLFS vs. w/ 1Hz pre-oLFS) | 11 |  |  |
|  |  |  |  |  |  |
|  |  | Difference ± SEM | 95% CI | R² value | P value |
|  |  | -127.7 ± 38.40 | -213.2 to -42.12 | 0.5251 | < 0.01 |
|  |  |  |  |  |  |
|  |  |  |  |  |  |
| **Seizure probability [%]** | **Fig. 5I** | Wilcoxon matched-pairs signed rank test |  |  |  |
|  |  | Number of pairs (w/o oLFS vs. w/ 1Hz pre-eLFS) | 4 |  |  |
|  |  |  |  |  |  |
|  |  | Median of differences | Sum of positive, negative ranks |  | P value |
|  |  | -70.84 | 0.000 , -10.00 |  | > 0.05 |
|  |  |  |  |  |  |
|  |  |  |  |  |  |
| **Mean Racine scale** | **Fig. 5J** | Wilcoxon matched-pairs signed rank test |  |  |  |
|  |  | Number of pairs (w/o oLFS vs. w/ 1Hz pre-oLFS) | 4 |  |  |
|  |  |  |  |  |  |
|  |  | Median of differences | Sum of positive, negative ranks |  | P value |
|  |  | -2 | 1 , -10 |  | > 0.05 |
|  |  |  |  |  |  |
|  |  |  |  |  |  |
| **AUC (10 Hz) [au]** | **Fig. 5K** | Paired Student's t test. two-tailed |  |  |  |
|  |  | Number of pairs (w/o eLFS vs. w/ 1Hz pre-eLFS) | 4 |  |  |
|  |  |  |  |  |  |
|  |  | Difference ± SEM | 95% CI | R² value | P value |
|  |  | -24.20 ± 32.19 | -126.7 to 78.26 | 0.1585 | > 0.05 |
|  |  |  |  |  |  |
|  |  |  |  |  |  |
| **Seizure probability [%]** | **Fig5, S2C** | Wilcoxon matched-pairs signed rank test |  |  |  |
|  |  | Number of pairs (w/o oLFS vs. w/ 0.5Hz pre-oLFS) | 10 |  |  |
|  |  |  |  |  |  |
|  |  | Median of differences | Sum of positive, negative ranks |  | P value |
|  |  | -100 | 0 , -66 |  | < 0.001 |
|  |  |  |  |  |  |
|  |  |  |  |  |  |
| **Mean Racine scale** | **Fig5, S2D** | Wilcoxon matched-pairs signed rank test |  |  |  |
|  |  | Number of pairs (w/o oLFS vs. w/ 1Hz pre-oLFS) | 10 |  |  |
|  |  |  |  |  |  |
|  |  | Median of differences | Sum of positive, negative ranks |  | P value |
|  |  | -2.52 | 1 , -54 |  | < 0.01 |
|  |  |  |  |  |  |
|  |  |  |  |  |  |
| **AUC (10 Hz) [au]** | **Fig5, S2E** | Paired Student's t test. two-tailed |  |  |  |
|  |  | Number of pairs (w/o oLFS vs. w/ 0.5Hz pre-oLFS) | 10 |  |  |
|  |  |  |  |  |  |
|  |  | Difference ± SEM | 95% CI | R² value | P value |
|  |  | -116.8 ± 53.90 | -238.7 to 5.144 | 0.3428 | > 0.05 |
|  |  |  |  |  |  |
|  |  |  |  |  |  |
| **Number of action potentials per trial** | **Fig. 7C** | Two-way ANOVA |  |  |  |
| **(saline)** |  |  |  |  |  |
|  |  | Number of columns (stimulation intensity, treatment) | 2 |  |  |
|  |  | F (stimulation intensity: 20, 40, 60, 80, 100 mV) | 10.213 |  |  |
|  |  | F (treatment: no oLFS, oLFS) | 8.289 |  |  |
|  |  |  |  |  |  |
|  |  | Source of Variation | % of total variation | P value summary |  |
|  |  | Interaction |  | < 0.001 |  |
|  |  | stimulation intensity |  | < 0.0001 |  |
|  |  | treatment |  | < 0.01 |  |
|  |  |  |  |  |  |
|  |  | Bonferroni t-test |  |  |  |
|  |  | Groups (no oLFS vs. oLFS) | Mean Diff. | 95% CI | P values |
|  |  | within 20 mV | 0.0000 |  | > 0.05 |
|  |  | within 40 mV | 0.0000 |  | > 0.05 |
|  |  | within 60 mV | 0.0200 |  | > 0.05 |
|  |  | within 80 mV | 0.2404 |  | > 0.05 |
|  |  | within 100 mV | 1.5438 |  | < 0.05 |
|  |  |  |  |  |  |
| **Number of action potentials per trial** | **Fig. 7D** | Two-way ANOVA |  |  |  |
| **(KA)** |  |  |  |  |  |
|  |  | Number of columns (stimulation intensity, treatment) | 2 |  |  |
|  |  | F (stimulation intensity: 20, 40, 60, 80, 100 mV) | 19.688 |  |  |
|  |  | F (treatment: no oLFS, oLFS) | 11.695 |  |  |
|  |  |  |  |  |  |
|  |  | Source of Variation | % of total variation | P value summary |  |
|  |  | Interaction |  | > 0.05 |  |
|  |  | stimulation intensity |  | < 0.0001 |  |
|  |  | treatment |  | < 0.001 |  |
|  |  |  |  |  |  |
|  |  | Bonferroni t-test |  |  |  |
|  |  | Groups (no oLFS vs. oLFS) | Mean Diff. | 95% CI | P values |
|  |  | within 20 mV | 0.5789 |  | > 0.05 |
|  |  | within 40 mV | 5.9474 |  | > 0.05 |
|  |  | within 60 mV | 9.5277 |  | > 0.05 |
|  |  | within 80 mV | 10.0671 |  | > 0.05 |
|  |  | within 100 mV | 14.2480 |  | < 0.05 |
|  |  |  |  |  |  |
| **IPSCs Frequency [Hz]** | **Fig. 7E** | Mann-Whitney Rank Sum Test |  |  |  |
|  |  | Number of cells (KA) | 6 |  |  |
|  |  | Number of cells (Saline) | 7 |  |  |
|  |  |  |  |  |  |
|  |  | Equal Variance Test (Brown-Forsythe): |  |  | P value |
|  |  | Failed |  |  | < 0.05 |
|  |  |  |  |  |  |
|  |  |  |  |  |  |
| **IPSCs Frequency [Hz]** | **Fig. 7E** | Mann-Whitney Rank Sum Test |  |  |  |
|  |  | Number of cells (KA) | 6 |  |  |
|  |  | Number of cells (Saline) | 7 |  |  |
|  |  |  |  |  |  |
|  |  | Equal Variance Test (Brown-Forsythe): |  |  | P value |
|  |  | Failed |  |  | < 0.05 |
|  |  |  |  |  |  |
|  |  |  |  |  |  |
| **High-load burst ratio (idHC)** | **Fig. 8C** | Mixed-effects model (REML) |  |  |  |
| **week 1** |  | P value | < 0.0001 |  |  |
|  |  | Number of groups | 4 |  |  |
|  |  | Number of values per group | 32, 32, 32, 14 |  |  |
|  |  | Chi-square, df | 14.38, 1 |  |  |
|  |  |  |  |  |  |
|  |  | Tukey's Multiple Comparison Test |  |  |  |
|  |  | Groups | Mean Diff. | 95% CI | P values |
|  |  | pre vs. 1 Hz eLFS | 0.1755 | 0.1323 to 0.2187 | < 0.0001 |
|  |  | pre vs. post 1 | 0.08175 | 0.04571 to 0.1178 | < 0.0001 |
|  |  | pre vs. post 2 | 0.03658 | -0.008593 to 0.08176 | > 0.05 |
|  |  | 1 Hz eLFS vs. post 1 | -0.09378 | -0.1277 to -0.05984 | < 0.0001 |
|  |  | 1 Hz eLFS vs. post 2 | -0.1389 | -0.1867 to -0.09122 | < 0.0001 |
|  |  | post 1 vs. post 2 | -0.04517 | -0.08846 to -0.001874 | < 0.05 |
|  |  |  |  |  |  |
| **Epileptic spike rate [Hz]** | **Fig. 8D** | Mixed-effects model (REML) |  |  |  |
| **(idHC) week 1** |  | P value | < 0.001 |  |  |
|  |  | Number of groups | 4 |  |  |
|  |  | Number of values per group | 32, 32, 32, 15 |  |  |
|  |  | Chi-square, df | 1.546, 1 |  |  |
|  |  |  |  |  |  |
|  |  | Tukey's Multiple Comparison Test |  |  |  |
|  |  | Groups | Mean Diff. | 95% CI | P values |
|  |  | pre vs. 1 Hz eLFS | 0.6125 | 0.4612 to 0.7638 | < 0.0001 |
|  |  | pre vs. post 1 | 0.2349 | 0.1142 to 0.3556 | < 0.0001 |
|  |  | pre vs. post 2 | 0.06688 | -0.07052 to 0.2043 | > 0.05 |
|  |  | 1 Hz eLFS vs. post 1 | -0.3776 | -0.4762 to -0.2789 | < 0.0001 |
|  |  | 1 Hz eLFS vs. post 2 | -0.5456 | -0.7292 to -0.3620 | < 0.0001 |
|  |  | post 1 vs. post 2 | -0.168 | -0.3085 to -0.02754 | < 0.05 |
|  |  |  |  |  |  |
| **High-load burst ratio (idHC)** | **Fig. 8F** | Mixed-effects model (REML) |  |  |  |
| **week 2** |  | P value | < 0.001 |  |  |
|  |  | Number of groups | 4 |  |  |
|  |  | Number of values per group | 34, 34, 34, 31 |  |  |
|  |  | Chi-square, df | 5.991, 1 |  |  |
|  |  |  |  |  |  |
|  |  | Tukey's Multiple Comparison Test |  |  |  |
|  |  | Groups | Mean Diff. | 95% CI | P values |
|  |  | pre vs. 1 Hz eLFS | 0.1936 | 0.1620 to 0.2252 | < 0.0001 |
|  |  | pre vs. post 1 | 0.08024 | 0.03625 to 0.1242 | < 0.001 |
|  |  | pre vs. post 2 | 0.01711 | -0.01612 to 0.05034 | > 0.05 |
|  |  | 1 Hz eLFS vs. post 1 | -0.1133 | -0.1408 to -0.08589 | < 0.0001 |
|  |  | 1 Hz eLFS vs. post 2 | -0.1765 | -0.1972 to -0.1557 | < 0.0001 |
|  |  | post 1 vs. post 2 | -0.06313 | -0.09041 to -0.03584 | < 0.0001 |
|  |  |  |  |  |  |
| **Epileptic spike rate [Hz]** | **Fig. 8G** | Mixed-effects model (REML) |  |  |  |
| **(idHC) week 2** |  | P value | < 0.0001 |  |  |
|  |  | Number of groups | 4 |  |  |
|  |  | Number of values per group | 34, 34, 34, 31 |  |  |
|  |  | Chi-square, df | 30.24, 1 |  |  |
|  |  |  |  |  |  |
|  |  | Tukey's Multiple Comparison Test |  |  |  |
|  |  | Groups | Mean Diff. | 95% CI | P values |
|  |  | pre vs. 1 Hz eLFS | 0.6478 | 0.5405 to 0.7551 | < 0.0001 |
|  |  | pre vs. post 1 | 0.2032 | 0.09463 to 0.3118 | < 0.0001 |
|  |  | pre vs. post 2 | 0.03463 | -0.04841 to 0.1177 | > 0.05 |
|  |  | 1 Hz eLFS vs. post 1 | -0.4446 | -0.5191 to -0.3702 | < 0.0001 |
|  |  | 1 Hz eLFS vs. post 2 | -0.6132 | -0.6896 to -0.5368 | < 0.0001 |
|  |  | post 1 vs. post 2 | -0.1686 | -0.2435 to -0.09365 | < 0.0001 |
|  |  |  |  |  |  |
| **High-load burst ratio** | **Fig. 8H** | Two-way ANOVA |  |  |  |
| **(idHC)** |  |  |  |  |  |
|  |  | Number of animals | 7 |  |  |
|  |  | Number of repetitions per animal | 2 |  |  |
|  |  | F (6, 21) | 2.88 |  |  |
|  |  |  |  |  |  |
|  |  | Source of Variation | % of total variation | P value summary |  |
|  |  | Interaction | 14.04 | ns |  |
|  |  | session | 11.83 | ns |  |
|  |  | animal | 33.46 | < 0.05 |  |
|  |  |  |  |  |  |
|  |  | Tukey's multiple comparisons test |  |  |  |
|  |  | Groups | Mean Diff. | 95% CI | P values |
|  |  | LFP 1st hour vs. LFP 2nd hour | -0.05157 | -0.1131 to 0.009943 | > 0.05 |
|  |  | LFP 1st hour vs. LFP 3rd hour | -0.05286 | -0.1144 to 0.008658 | > 0.05 |
|  |  | LFP 2nd hour vs. LFP 3rd hour | -0.001286 | -0.06280 to 0.06023 | > 0.05 |
|  |  |  |  |  |  |
| **High-load burst ratio** | **Fig. 8I** | Two-way ANOVA |  |  |  |
| **(idHC)** |  |  |  |  |  |
|  |  | Number of columns (animal) | 6 |  |  |
|  |  | Number of repetitions per animal | 2 |  |  |
|  |  | F (5, 30) animal | 8.014 |  |  |
|  |  |  |  |  |  |
|  |  | Source of Variation | % of total variation | P value summary |  |
|  |  | Interaction | 9.978 | < 0.001 |  |
|  |  | session | 81.07 | < 0.0001 |  |
|  |  | animal | 5.121 | < 0.0001 |  |
|  |  |  |  |  |  |
|  |  | Tukey's multiple comparisons test |  |  |  |
|  |  | Groups | Mean Diff. | 95% CI | P values |
|  |  | pre vs. 1 Hz eLFS 1st hour | 0.1682 | 0.1439 to 0.1925 | < 0.0001 |
|  |  | pre vs. 1 Hz eLFS 2nd hour | 0.174 | 0.1497 to 0.1983 | < 0.0001 |
|  |  | pre vs. 1 Hz eLFS 3rd hour | 0.1734 | 0.1491 to 0.1977 | < 0.0001 |
|  |  | pre vs. post1 | 0.1173 | 0.09302 to 0.1416 | < 0.0001 |
|  |  | 1 Hz eLFS 1st hour vs. 1 Hz eLFS 2nd hour | 0.005833 | -0.01848 to 0.03015 | > 0.05 |
|  |  | 1 Hz eLFS 1st hour vs. 1 Hz eLFS 3rd hour | 0.00525 | -0.01906 to 0.02956 | > 0.05 |
|  |  | 1 Hz eLFS 1st hour vs. post1 | -0.05083 | -0.07515 to -0.02652 | < 0.0001 |
|  |  | 1 Hz eLFS 2nd hour vs. 1 Hz eLFS 3rd hour | -0.0005833 | -0.02490 to 0.02373 | > 0.05 |
|  |  | 1 Hz eLFS 2nd hour vs. post1 | -0.05667 | -0.08098 to -0.03235 | < 0.0001 |
|  |  | 1 Hz eLFS 3rd hour vs. post1 | -0.05608 | -0.08040 to -0.03177 | < 0.0001 |
|  |  |  |  |  |  |
| **High-load burst ratio** | **Fig8, S1B** | Mixed-effects model (REML) |  |  |  |
| **(idHC)** |  | P value | > 0.05 |  |  |
|  |  | Number of groups | 4 |  |  |
|  |  | Number of values per group | 9, 9, 9, 7 |  |  |
|  |  | Chi-square, df | 0.3656, 1 |  |  |
|  |  |  |  |  |  |
|  |  | Tukey's Multiple Comparison Test |  |  |  |
|  |  | Groups | Mean Diff. | 95% CI | P values |
|  |  | pre vs. 1 Hz eLFS | 0.004778 | -0.08955 to 0.09910 | > 0.05 |
|  |  | pre vs. post 1 | 0.009889 | -0.06791 to 0.08769 | > 0.05 |
|  |  | pre vs. post 2 | -0.01454 | -0.1324 to 0.1034 | > 0.05 |
|  |  | 1 Hz eLFS vs. post 1 | 0.005111 | -0.1030 to 0.1132 | > 0.05 |
|  |  | 1 Hz eLFS vs. post 2 | -0.01932 | -0.1515 to 0.1129 | > 0.05 |
|  |  | post 1 vs. post 2 | -0.02443 | -0.1522 to 0.1033 | > 0.05 |
|  |  |  |  |  |  |
| **Epileptic spike rate [Hz]** | **Fig8, S1B** | Mixed-effects model (REML) |  |  |  |
| **(idHC)** |  | P value | > 0.05 |  |  |
|  |  | Number of groups | 4 |  |  |
|  |  | Number of values per group | 9, 9, 9, 7 |  |  |
|  |  | Chi-square, df | 0.1856, 1 |  |  |
|  |  |  |  |  |  |
|  |  | Tukey's Multiple Comparison Test |  |  |  |
|  |  | Groups | Mean Diff. | 95% CI | P values |
|  |  | pre vs. 1 Hz eLFS | 0.1617 | 0.04669 to 0.2766 | < 0.01 |
|  |  | pre vs. post 1 | -0.003778 | -0.2030 to 0.1955 | > 0.05 |
|  |  | pre vs. post 2 | -0.1226 | -0.4807 to 0.2355 | > 0.05 |
|  |  | 1 Hz eLFS vs. post 1 | -0.1654 | -0.3610 to 0.03009 | > 0.05 |
|  |  | 1 Hz eLFS vs. post 2 | -0.2843 | -0.6682 to 0.09968 | > 0.05 |
|  |  | post 1 vs. post 2 | -0.1188 | -0.5775 to 0.3399 | > 0.05 |
|  |  | 1 Hz eLFS vs. post 2 | -0.01932 | -0.1515 to 0.1129 | > 0.05 |
|  |  | post 1 vs. post 2 | -0.02443 | -0.1522 to 0.1033 | > 0.05 |
|  |  |  |  |  |  |
| **Epileptic spike rate [Hz]** | **Fig8, S1B** | Mixed-effects model (REML) |  |  |  |
| **(idHC)** |  | P value | > 0.05 |  |  |
|  |  | Number of groups | 4 |  |  |
|  |  | Number of values per group | 9, 9, 9, 7 |  |  |
|  |  | Chi-square, df | 0.1856, 1 |  |  |
|  |  |  |  |  |  |
|  |  | Tukey's Multiple Comparison Test |  |  |  |
|  |  | Groups | Mean Diff. | 95% CI | P values |
|  |  | pre vs. 1 Hz eLFS | 0.1617 | 0.04669 to 0.2766 | < 0.01 |
|  |  | pre vs. post 1 | -0.003778 | -0.2030 to 0.1955 | > 0.05 |
|  |  | pre vs. post 2 | -0.1226 | -0.4807 to 0.2355 | > 0.05 |
|  |  | 1 Hz eLFS vs. post 1 | -0.1654 | -0.3610 to 0.03009 | > 0.05 |
|  |  | 1 Hz eLFS vs. post 2 | -0.2843 | -0.6682 to 0.09968 | > 0.05 |
|  |  | post 1 vs. post 2 | -0.1188 | -0.5775 to 0.3399 | > 0.05 |

**Supplementary file 1: Quantitative summary of statistically tested parameters.** The table displays all results statistical tests performed (right column) for each parameter (left column). The reference to the corresponding figure is given in the middle column. Diff., difference; CI, confidence interval; eLFS, electrical low-frequency stimulation; LFP, local field potential, oLFS, optogenetic low-frequency stimulation; w/, with; w/o, without;
