# Supplementary material for: Hippocampal low-frequency stimulation prevents seizure generation in a mouse model of mesial temporal lobe epilepsy
Source: eLife. 2020 Dec 22;9:e54518. doi: 10.7554/eLife.54518 (PMC7800381; doi:10.7554/eLife.54518)
Supplement: Peer review history

# Decision letter

**DOI:** 10.7554/eLife.54518.sa1

**Affiliations:** Stanford University School of MedicineUnited States; Kennedy Krieger Institute and Johns Hopkins University School of MedicineUnited States

In the interests of transparency, eLife publishes the most substantive revision requests and the accompanying author responses.

**Acceptance summary:**

Paschen et al. show that optogenetic stimulation of medial entorhinal cortical fibers or hippocampal neurons at relative low frequency around 1 Hz inhibits seizures in a model of chronic temporal lobe epilepsy. The experiments are performed to a very high standard, and include a painstaking and detailed characterization of the intrahippocampal kainate model, with detailed neuropathological and in-vivo electrographic analysis of the model with different concentrations of kainate and different amounts of hippocampal damage,as well as analysis of low frequency effects, with 1 Hz delivering more efficacy than lower frequencies.

**Decision letter after peer review:**

Thank you for submitting your article "Optogenetic low-frequency stimulation of dentate granule cells prevents seizure generation in experimental epilepsy" for consideration by *eLife*. Your article has been reviewed by John Huguenard as the Senior and Reviewing Editor, and three reviewers.

The reviewers have discussed the reviews with one another and the Reviewing Editor has drafted this decision to help you prepare a revised submission.

Summary:

Paschen et al., show that optogenetic stimulation of medial entorhinal cortical fibers or hippocampal neurons inhibits seizures in a model of chronic temporal lobe epilepsy. Optogenetic inhibition of seizures has been described in the literature in different types of models, and targeting different cell types selectively. The experiments are performed to a very high standard, and include a painstaking and detailed characterization of the kainate model, with detailed neuropathological and in-vivo electrographic analysis of the model with different concentrations of kainate and different amounts of hippocampal damage. Different stimulus frequencies were tested for seizure control, with the most robust effects with a relatively low rate of 1 Hz.

Essential revisions:

While all reviewers noted strengths of the findings, one overall major issue is the specificity of the LF stimulation (see points 2,5,7,10,14, below). It is critical that in the revised manuscript, it must be clearly demonstrated that the stimuli are specifically activating perforant path synapses onto the dentate gyrus granule cells.

1) The major deterioration of granule cell excitability with just 10 min of oLFS in vitro can make it challenging to interpret what might be happening in vivo during the 1hour stimulation sessions. It is also unclear what effects chronic oLFS would have in epileptic animals (e.g., over days) as the 1hour stimulation sessions in 2 successive days is quite short.

2) The authors assume that the mechanism underlying seizure reduction is due to reduced glutamate release at perforant pathway-granule cell synapses. How did the responses in epileptic animals compare to control animals? Were paired pulse responses of evoked EPCs also altered in granule cells? What effects does oLFS have on inhibition in the dentate gyrus?

3) Given the dramatic effect on glutamatergic transmission, it is possible that normal hippocampal function is disrupted by oLFS. Were the mice evaluated in memory tests?

4) Using optogenetic stimulation to drive convulsive seizures as well as to stop is potentially problematic. Did the authors test these mice for spontaneous convulsive seizures? Convulsive seizures have been shown to occur regularly in this model around 15 weeks after KA administration (Krook-Magnuson, 2013).

5) How do the authors know they are specifically activating entorhinal input only to dentate gyrus and not to other hippocampal regions, such as CA1 or CA3?

6) Only 1-3days between implantation surgery and initiating the recording sessions is very short.

7) What effect does oLFS outside of the sclerotic area have on seizures (e.g., oLFS in the contralateral hippocampus)?

8) There are serious concerns with the current form of the manuscript specifically with the translational interpretation of the results. The techniques, data findings, and concepts in this manuscript are seemingly being proposed as a viable alternative to the hippocampal anterior horn resection surgery (anatomically ventral lobe of mouse hippocampus) for patients with refractory MTLE. Primary evidence is not provided to explain why the authors assume seizure recurrence rates would be lower with the optogenetic cell-type specific stimulation. Additionally, first generation deep brain stimulation paradigms that are open loop are antiquated as they lack long-term efficacy. In this regard, closed-loop (on demand) deep brain stimulation in conjunction with resective surgeries are already in practice and report benefits for cases where the epileptic focus is not discrete nor amenable to complete resection (Ma et al., 2020). Resective epilepsy surgery provides seizure freedom to the majority of patients with pharmacoresistant focal MTLE (please see review by Englot and Chang, 2014). Early surgical intervention is a predictor for improved seizure freedom for patients with temporal lobe epilepsy (Engel et al., 2003;Wiebe, 2001). Patients with bilateral epileptic foci, or who are at risk for severe memory impairments, may not be ideal candidates for surgical resection. Deep brain stimulation is one alternative surgical therapy for these patients. The optimal anti-ictogenic stimulation parameters for DBS in MTLE have not been identified/there is no clear consensus, however multiple surgery centers are already reporting efficacy results for closed-loop DBS in patients. The current manuscript is not positioned to compare the efficacy between pharmacological intervention, surgical resection, and DBS stimulation (either electrical or optogenetic) for these reasons:

A) For translational relevance as a therapeutic intervention pharmacoresistance should first be demonstrated in this model, with refractoriness to anti-seizure drugs quantified with the similar parameters reported here (e.g. High load burst ratio, Epileptic spike rate, seizure probability, etc.). Without this essential data the manuscript does not need to claim a translational relevance (see Abstract) nor suggest an improved outcome of oLFS vs. all other therapeutic interventions. None of these statements are directly supported by any data in the manuscript. Abstract "As an alternative to curative epilepsy surgery, brain stimulation evolves as a promising approach for seizure-interference." Electrical deep brain stimulation is a type of epilepsy surgery and should be portrayed as such. Abstract "exhibits unprecedented anti-ictogenic effects in chronically epileptic mice." It is unclear what oLFS efficacy is being clinically compared to throughout the manuscript. Is it resective epilepsy surgery, high frequency deep brain stimulation, or pharmacoresistance? Introduction "However, many patients do not remain seizure-free following curative epilepsy surgery (Mohan et al., 2018; Ryvlin and Kahane, 2005), thus demonstrating the urgent need for new therapeutic avenues." This statement is not supported by the current citations. It would benefit the reader to cite robust Class I evidence or meta-analyses data specifically investigating long-term outcomes of resective epilepsy surgery in MTLE. All assessments of clinical data should provide, at the minimum, the rate of seizure freedom and time post-surgery (e.g. 50-75% seizure free at 1 year). Introduction "However, in MTLE with severe hippocampal sclerosis, electrical stimulation appears remarkably ineffective…(Velasco et al., 2007)" It is not clear what the efficacy of electrical stimulation in MTLE is being compared to. Velasco et al., 2007 does not support the above statement. Rather, the conclusions of Velasco et al., 2007 are much more nuanced as stated by the authors in the Discussion. Discussion – here the authors portray the potential translational utility for this data in a weighted and clear manner, in contrast to the Introduction.

B) It is important to note that all interventions reported in this manuscript are acute time points, and all comparisons to clinical data are invalid as these outcomes are at much longer time scales (many months). Most anti-seizure drugs if tested would be effective during the sub-acute temporal periods chosen for the recordings. At the minimum, the limitation of acute stimulation parameters should be discussed.

9) Bilateral hippocampus

It is unclear if the bilateral hippocampus exhibits spontaneous discharges in this model. Figure 3A-B shows a dose dependent generalization of epileptiform activity into the contralateral dorsal hippocampus, but there is no quantification of this important parameter in Figure 3, Figure 4, Figure 5. Does this model involve the kindling of the contralateral hippocampus over time with the 15mM KA dose? Does idHC oLFS suppress cdHC epileptiform activity? Are there any KA dose differences? This quantification is important as bilateral epileptic foci are an important parameter for MTLE DBS candidates.

10) Cell-type specificity

Introduction The authors state that the stimulation of interneurons in preclinical mTLE increases seizure probability (Lévesque et al., 2019). This citation is from a systemic PTZ model with different stimulation parameters. The authors should either demonstrate the inability of optogenetic activation of interneurons to prevent ictogenesis in their model (potentially via the loss of GABAergic interneurons etc.) or remove statements claiming that targeting interneurons are ineffective as further studies are required in this model. Further, it would benefit readers to delineate stimulation differences when referencing other clinical or preclinical work (i.e. closed vs. open loop).

11) Frequency dependency

From the current manuscript it is clear that there are frequency dependent anti-seizure differences. However, low frequency stimulation is performed because the authors state that high frequency stimulation is ineffective. The authors should either demonstrate the inability of high frequency stimulation to prevent ictogenesis in their model, or remove statements claiming that they are ineffective.

12) Closed loop neuromodulation

Open-loop continuous stimulation is prone to failure over time as the approach does adapt to natural fluctuations, disease progression, or nervous system adaptations. In contrast, closed-loop neurostimulation is a rapidly emerging technology positioned to overcome the shortcomings of open-loop continuous stimulation. Therefore, brain responsive closed-loop neurostimulation (RNS System) devices should be extensively discussed in the context of this current manuscript (Geller et al., 2017and Van Ness et al., 2016). Previous closed-loop preclinical studies utilizing optogenetics in a unilateral KA model have demonstrated that on-demand inhibition of DGCs is anti-ictogenic (Krook-Magnuson et al., 2015; Krook-Magnuson et al., 2013), discussing this work in detail would benefit the reader.

13) The authors do not address or nor justify the exclusion of female mice in this study.

14) It is important to note that this paper uses different types of stimulation, which are not entirely well described in the paper (as dentate granule cell stimulation in the title). I think this latter phrasing is a little misleading. The light stimulation is firstly done as a stimulation of MEC fibers expressing ChR2 within the dentate gyrus. This is important, because even if the light stimulation is local within the dentate gyrus, retrograde firing of EC neurons will potentially lead to more wide-spread network effects. This should be acknowledged and discussed. Secondly, a more direct stimulation of neurons within the dentate gyrus is achieved with injection of virus at the same location as the kainate injection. However, also this viral transduction technique is not specific to DGCs, as the authors suggest at several points. The AAV used has a CaMKIIa promoter, which will express not only in DG neurons, but potentially also in other types of neurons (i.e. adjacent CA3 cells, potentially mossy cells, CA1 neurons), in particular since the injection location for kainate as far as I interpret the coordinates is not specifically located within the DG. Because light delivery is likewise never very specific, this means that the authors may stimulate a much more distributed hippocampal cell population. The figure panel 5B looks consistent with granule cell expression, but does not really give a precise idea of non-granule cell expression, in particular in the hilus and CA3 areas. There must be an analysis of expression patterns of hippocampal ChR2 using post-hoc immunohistochemistry, with a quantification of neurons in dentate, hilus and CA3/1 areas for all animals to be able to interpret the data clearly. Regarding the MEC virus injections, the MEC is not easy to target – the authors should demonstrate that they indeed target MEC in all animals by quantifying the expected band of mCherry positive fibers in the middle molecular layer of the dentate gyrus in those animals included in the study. Given the importance of this issue, I think it would be good to show more definitive quantification than the example shown in Figure 4C (which does look convincing). Given that the authors are saying that this may be a promising avenue for treatment, I think it is important to get a better sense of what is actually being stimulated in these experiments. This is particularly true given that in the Krook-Manguson, 2015 paper , selective expression of NpHR and ChR2 only in granule cells has been done. In these cases, stimulation of granule cells has resulted in induction of seizures and increased seizure progression to large behavioral seizures. This is even consistent with the authors data from figure 6 in my view, and I was really surprised that the authors have not discussed this. Does this not support the idea that the key factor deciding on seizure generation or reduction is stimulation frequency? This is also relevant for translation.

I also think that the paper by Bui et al., (2018) should be cited, this is a pretty glaring omission, as this paper has studied the effects of selectively manipulating mossy cells on seizures in a temporal lobe epilepsy, a cell type that is present within the dentate hilus and is known to strongly affect excitability of granule cells. This is clearly very relevant to the present paper. Incidentally, this paper also contains a figure of on-demand inhibition of dentate granule cells (Supplementary figure 11), which strongly truncates seizures. In the context of the authors hypothesis of how LFS works, this is also clearly relevant.

15) Materials and methods: results for ANOVA should be reported fully according to established guidelines (i.e. APA guidelines), throughout the manuscript. In addition, it should always be clear what the p-values refer to (i.e. in subsection “Variability of hippocampal sclerosis and seizure burden in chronically epileptic mice” a p-value is given for Figure 2B, but it is not explicit if this is for the ANOVA or one of the post tests.

There should be at least a brief account of how the analysis of the burst events was done in the Results section that allows the reader to get an idea of what these classifications mainly report. It is somewhat irksome to read the Results, refer to the Materials and methods, which also does not carry more information about this classification, and instead refers to a prior paper (Heining et al., 2019). If I understood it correctly, the recording sites in the Heining et al., paper were identical to those analyzed in this paper (this should be mentioned explicitly), but the injections were confined to 20 mM kainate in the previous paper. I assume that the authors have considered these following issues, but it is not clear from the manuscript. Firstly, one would like to see the SOMs that are the basis of the classification at least in some examples to see how well this discrimination works for the more subtle variant of this model. Also, it is unclear to me how exactly the classification will be comparable between the different kainate model variants. The SOM may classify into three categories in all three models, but in this case the average spike load indexes for the three categories might be different in the different epilepsy models. At this point it is not very easy for me to see what the differences in the composition of burst classes actually means when translated into firing dynamics in the different systems. I do appreciate the importance at looking at structure in high-dimensional data using such methods, but I think the authors could do a much better job at explaining this. I also think the authors should compute, and give some basic statistics of spiking in the different models (frequencies, inter-event interval distributions, etc.). This may not be the best way to show differences between models, but I do think it is important to evaluate the performance of the SOM protocol.

16) The slice recordings, together with the in-vivo opto-LFP recordings support a decrease in cellular excitability following light stimulation. This is interesting, but I think preliminary. One of the key findings in-vivo is that 1Hz stimulation induces a lasting effect. Therefore, one would have liked to see some data on whether this is a persistent effect, that outlasts the effects of the optical stimulation. It is not per se surprising that there is a decrease in neurotransmission upon prolonged afferent stimulation. Do the authors have some idea of what happens after the stimulus train? They probably have at least a subset of neurons that they were able to continue measuring after the stimulation train.

[Editors' note: further revisions were suggested prior to acceptance, as described below.]

Thank you for resubmitting your article "Hippocampal low-frequency stimulation prevents seizure generation in a mouse model of mesial temporal lobe epilepsy" for consideration by *eLife*. Your revised article has been reviewed by John Huguenard as the Senior Editor and Reviewing Editor, and two reviewers. The following individuals involved in review of your submission have agreed to reveal their identity: Robert Hunt (Reviewer #1); Shilpa D Kadam with Brennan Sullivan (Reviewers #2).

The reviewers have discussed the reviews with one another and the Reviewing Editor has drafted this decision to help you prepare a revised submission.

We would like to draw your attention to changes in our revision policy that we have made in response to COVID-19 (https://elifesciences.org/articles/57162). Specifically, we are asking editors to accept without delay manuscripts, like yours, that they judge can stand as *eLife* papers without additional data, even if they feel that they would make the manuscript stronger. Thus the revisions requested below only address clarity and presentation.

Summary:

Paschen et al. show that optogenetic stimulation of medial entorhinal cortical fibers or hippocampal neurons inhibits seizures in a model of chronic temporal lobe epilepsy. Optogenetic inhibition of seizures has been described in the literature in different types of models, and targeting different cell types selectively. The experiments are performed to a very high standard, and include a painstaking and detailed characterization of the kainate model, with detailed neuropathological and in-vivo electrographic analysis of the model with different concentrations of kainate and different amounts of hippocampal damage. Different stimulus frequencies were tested for seizure control, with the most robust effects with a relatively low rate of 1 Hz.

Essential revisions:

Please see the detailed comments for your information below. The editors and reviewers agree that the following two issues need to be addressed. It is expected this should not require any additional experiments. First, please modify the text to remove any remaining claims that are not supported by statistical analysis. We think the best approach is simply to provide the quantified population results and the statistical test results. Secondly, there remains an issue of specificity of pathway stimulation. It is not entirely clear that the optogenetic approach is only affecting the MEC to DG circuit in vivo. The authors rely only on staining in the DG molecular layer as evidence of specificity. However, given the anatomy of the entorhinal-hippocampal circuit and the experimental design to target dorsal blade of DG, it would really help to show that stimulation produces no response in CA1. Alternatively, please provide images of sagittal sections documenting that virus injection was restricted to MEC, as is standard practice for these types of experiments.

Detailed comments of reviewers for this revision:

Reviewer #1:

The authors addressed almost all concerns from the prior review and added some important new data. However, I'm still not entirely convinced that the MEC to DG circuit was selectively targeted. The mCherry staining in middle molecular layer of DG is convincing, but this isn't as direct as showing successful viral targeting of only MEC (e.g., in a sagittal section). At least one animal shows mCherry staining in outer molecular layer (EP8, Figure S2). In all of the example images shown, there is also robust mCherry staining in slm of CA1 across its transverse axis. i.e., there is staining in both proximal CA1 (toward CA2) and distal CA1 (toward subiculum). MEC should preferentially project to proximal CA1 whereas LEC primarily projects to distal CA1 (Steward, 1976; Wyss, 1981; Tamamaki and Nojyo, 1995; Naber et al., 2001). Even though the pyramidal neurons are mostly lost in the epilepsy model, these mCherry-labeled MEC projections are likely synapsing onto any surviving neurons in CA1. Likewise, while it is clear that the perforant pathway is being activated in DG as the authors report, the optic fiber is extremely close to the MEC axons in CA1c (Figure S2). How do the authors know they are not activating these axons, in addition to the projections to DG?

Reviewer #2:

This is a revised submission by Paschen et al., of the manuscript entitled "Hippocampal low-frequency stimulation prevents seizure generation in a mouse model of mesial temporal lobe epilepsy" for publication in *eLife*. Overall, the experiments presented are of high quality and are recommended for publication. The remaining concerns are related to outstanding unsupported claims that require statistics for their inclusion in the final manuscript.

1) Frequency dependence of the LFS effects

A) Subsection “Application of different oLFS protocols during spontaneous epileptiform activity” "0.5 or 0.2 Hz also had suppressive effects on epileptiform activity, albeit less pronounced than the 1 Hz stimulation". No statistical analysis supports this statement. Figure 4P figure legend states "1Hz oLFS was the most effective stimulation frequency", however no statistical analysis supports this statement. Rather, Figure 4P demonstrates 1 and 0.5 being significantly better than 0.2, but no significant differences between 1Hz vs. 0.5Hz. This inaccuracy is also prevalent throughout the Discussion and somewhat contradictory to the logic purposed by the authors Introduction.

B) Subsection “Application of different oLFS protocols during spontaneous epileptiform activity”. "Interestingly, epileptiform activity was also suppressed in the contralateral hippocampus, with 1 Hz oLFS being most effective" No statistical analysis supports this statement, specifically "most effective".

C) Burst ratio efficacy [%] analysis was not performed for cdHC, and epileptic spike rate efficacy [%] graph is an essential analysis for cdHC and idHC.

2) Translational relevance still remains overstated.

A) The eLFS experiments demonstrate the acute effects (hours) of DBS over many weeks. These experiments are important as it demonstrates the ability for LFS to reduce epileptic activity, and the authors are commended for this work. However, the authors compare their results to clinic and to pharmacoresistance (broadly considered). This comparison is inaccurate as it does not acknowledge the limitation of analyzing anti-seizure effects over short durations of time (even if over many weeks) in their current manuscript. The authors' primary outcome of acute effects is categorically different than clinical primary outcomes (seizure diaries, continuous monitoring, etc.), and therefore such overstatements should be avoided.

B) For almost all studies cited the electrode location, frequency of stimulation, duration of stimulation, and primary outcomes were different. However, only differences in frequency parameters between studies were highlighted by the authors within the current manuscript e.g. Introduction. This is an oversimplification of the clinical data and should be revised as it is inaccurate. The closed-loop RNS system is tailored to each patient and considered a complementary therapy to anti-seizure medication, this is not reflected in the current manuscript.

C) The author's rebuttal response (point 13) and reasoning for not including female mice in their study is not reflected in the revised manuscript. This reasoning needs to be added to the methods section of the manuscript. The author's go to great lengths to compare their results to the clinic but omit female mice from their experiments, an evident contradiction.

# Reply: Author response

**DOI:** 10.7554/eLife.54518.sa2

Essential revisions:

While all reviewers noted strengths of the findings, one overall major issue is the specificity of the LF stimulation (see points 2,5,7,10,14, below). It is critical that in the revised manuscript, it must be clearly demonstrated that the stimuli are specifically activating perforant path synapses onto the dentate gyrus granule cells.

We are happy that all reviewers noted the strengths of our findings.

To begin with, we addressed the main concern of all reviewers regarding the specificity of the optogenetic stimulation. We share the view that our experiments do not fully exclude off-target effects. However, taking into account all our present results and previous findings (Janz et al., 2018, 2017), we have good reasons to assume that the stimulation of dentate granule cells (DGCs; indirectly via the perforant path) elicits the anti-epileptic effect of low-frequency stimulation (LFS). In the following, we will outline our arguments in detail:

– Anatomy: Since CA1 and CA3 pyramidal cells, GABAergic interneurons, as well as mossy cells are extensively diminished in the KA-injected hippocampus as shown in Figure 2 (subsection “Modification of the intrahippocampal KA mouse model”) and by others (Bouilleret et al., 2000, 1999; Marx et al., 2013), it is unlikely that stimulation of these cell types is essentially involved in the anti-epileptic action of oLFS. This point has been addressed in the revised manuscript (subsection “oLFS of entorhinal afferents interferes with spontaneous, recurrent seizures”).

– Opsin expression and light delivery: In the revised manuscript, we demonstrate that ChR2-mCherry is expressed in the middle molecular layer of the dentate gyrus of all animals included in the study (Figure 4—figure supplement 2). We quantified ChR2-mCherry expression by densitometric analysis of grey values (subsection “Image acquisition and analysis”) along the dorsoventral hippocampal axis in each animal. Peak expression was found in the middle molecular layer, the termination zone of the perforant path (Figure 4—figure supplement 2E). Weak ChR2-mCherry expression was also present in the alveus and the stratum lacunosum moleculare (i.e. the temporoammonic path). Since the optic fiber was positioned in the molecular layer, right above the perforant path, and the penetration depth in brain tissue is restricted to 500 µm under the given parameters (473 nm; 150 mW/mm² at the fiber tip; according to Yizhar et al., 2011), we estimate that in our case the radius of light emission did not exceed 500 µm (Figure 4—figure supplement 2; subsection “in vivo LFS experiments”). Therefore, we are convinced that the optical stimuli specifically activated perforant path synapses onto DGCs.

– Acute slice experiments: In our in vitro experiments, optical stimulation of the perforant path induced EPSPs in DGCs and was occasionally sufficient to elicit action potentials (Figure 7A, B; subsection “Cellular responses to oLFS”).

– Stimulation electrode position: In our new animal cohort (see point 1 below), we stimulated the dentate gyrus electrically (1 Hz) and obtained strong seizure-suppressive effects. However, in cases of misplaced stimulation electrodes (Figure 8—figure supplement 1) seizures were not suppressed, suggesting that the stimulation target, i.e. the dentate gyrus is important for a successful seizure interference in the intrahippocampal KA mouse model (subsection “Effects of repeated hippocampal eLFS on epileptiform activity”).

1) The major deterioration of granule cell excitability with just 10 min of oLFS in vitro can make it challenging to interpret what might be happening in vivo during the 1hour stimulation sessions. It is also unclear what effects chronic oLFS would have in epileptic animals (e.g., over days) as the 1hour stimulation sessions in 2 successive days is quite short.

Concerning the first point, we would like to emphasize that in vivo we observed a decrease of the cellular response within the first 10 min during one hour 1 Hz stimulation. In the following 50 min of oLFS, no further decline occurred (Figure 6D). That was the reason why we performed in vitro experiments focusing on the effects of 10 min oLFS. Interestingly, we saw a strong similarity to the in vivo situation: EPSP amplitudes of DGCs decreased within 10 min of oLFS indicating that also in vivo oLFS might reduce the efficacy of perforant path transmission onto DGCs (Figure 7).

Concerning the second point, we fully agree with the reviewer, that only two days of oLFS is quite short. Therefore, we extended the LFS (1 Hz) experiments in a new animal cohort and switched to electrical LFS (eLFS) which was performed daily for one hour over two weeks (Figure 8A-G; subsection “Effects of repeated hippocampal eLFS on epileptiform activity”). Additionally, one-hour eLFS was expanded to three hours in a continuous and discontinuous manner. In conclusion, eLFS in the sclerotic hippocampus had a strong seizure-suppressive effect, which (i) maintained its efficacy over several weeks without desensitization, (ii) could be prolonged for at least three hours, and (iii) remained stable in short stimulation-free phases (Figure 8K-L; subsection “Effects of repeated hippocampal eLFS on epileptiform activity”).

Piret Kleis has been added as an author at the revised stage since she contributed substantially to the new eLFS dataset. All authors agree with her inclusion and place in the author list.

2) The authors assume that the mechanism underlying seizure reduction is due to reduced glutamate release at perforant pathway-granule cell synapses. How did the responses in epileptic animals compare to control animals? Were paired pulse responses of evoked EPCs also altered in granule cells? What effects does oLFS have on inhibition in the dentate gyrus?

We would like to point out that reduced glutamate release is only one possible explanation or contributing factor for the anti-epileptic action of LFS. To address the first concern of the reviewer we discussed this point in more detail in the revised manuscript (subsection “LFS 488 reduces the efficacy of perforant path transmission onto DGCs”). The second point was answered by new in vivo (Figure 6A, B) and in vitro (Figure 7C) experiments. Compared to chronically epileptic mice, control animals responded similarly to one-hour oLFS in vivo (Figure 6B) and to 10 min oLFS in vitro (Figure 7C). In addition, we performed paired-pulse experiments in acute slices from epileptic mice before and after 10 min oLFS. We found a slight alteration for the 10 ms inter-stimulus interval in epileptic animals after oLFS (see Author response image 1, facilitation index of 1.3 ± 0.1 vs 1.7 ± 0.1 for control conditions and after 10 min oLFS, p = 0.018, paired t-test). Because the differences in paired-pulse facilitation were small, we decided not to include this data in the manuscript. Nevertheless, these results suggest a decrease in glutamate release probability in perforant path inputs after oLFS, supporting the hypothesis of short-term changes in synaptic dynamics of entorhinal fibers after ChR2-mediated 1 Hz stimulation.

Referring to the last question, we would like to point out (as mentioned above) that most GABAergic interneurons are lost in the sclerotic hippocampus. To corroborate these findings, we analyzed IPSCs in DGCs from epileptic and control animals (Figure 7E; subsection “Cellular responses to oLFS”). Similar to previous reports, we found that IPSC frequency was strongly reduced in GCs from KA-injected animals, confirming the absence of interneurons in the sclerotic hippocampus. This data, together with the fact that our in vitro experiments were made under conditions of pharmacological blockade of GABAergic transmission, strongly suggests that oLFS effects were mediated by changes in glutamatergic transmission and therefore we did not further analyze its influence on GABAergic inhibition.

**Author response image 1.**

3) Given the dramatic effect on glutamatergic transmission, it is possible that normal hippocampal function is disrupted by oLFS. Were the mice evaluated in memory tests?

We fully agree with the reviewer’s concern that hippocampal function, especially learning and memory might be potentially disrupted by LFS. Hence, we first evaluated the basic running behavior of mice before, during, and after oLFS and did not see major changes in this behavior (Figure 4—figure supplement 4). Further experiments will be needed to investigate the potential effects of hippocampal LFS on learning and memory as we mention now in the revised manuscript (subsection “LFS 488 reduces the efficacy of perforant path transmission onto DGCs”). In our view, this exceeds the scope of the current manuscript and will be addressed in a follow-up publication.

4) Using optogenetic stimulation to drive convulsive seizures as well as to stop is potentially problematic. Did the authors test these mice for spontaneous convulsive seizures? Convulsive seizures have been shown to occur regularly in this model around 15 weeks after KA administration (Krook-Magnuson, 2013).

We addressed this concern by combining electrical and optogenetic stimulation (subsection “Effects of oLFS and eLFS on induced behavioral seizures”). We applied 30 min eLFS in the sclerotic hippocampus before we started the optogenetic pro-convulsive stimulus (Figure 5H-K). We could demonstrate a promising preconditioning effect of 1 Hz eLFS. In our LFP recordings (up to seven weeks after KA administration), we observed that spontaneous recurrent seizures rarely generalized. This is in line with previous reports for the intrahippocampal KA mouse model (Bouilleret et al., 1999; Klein et al., 2015).

5) How do the authors know they are specifically activating entorhinal input only to dentate gyrus and not to other hippocampal regions, such as CA1 or CA3?

We addressed this major concern in the beginning of the letter.

6) Only 1-3 days between implantation surgery and initiating the recording sessions is very short.

In the initial manuscript, the illustrated timeline in Figure 1 was misleading. We corrected the Figure in the revised version (Figure 1). Animals were checked regularly following implantations and they all recovered within two to three days. Recording sessions were only started when mice showed normal behavior in their home cage, such as feeding, grooming, and interacting with their littermates.

7) What effect does oLFS outside of the sclerotic area have on seizures (e.g., oLFS in the contralateral hippocampus)?

To address this concern in the revised version, we analyzed data from the contralateral hippocampus and show that epileptiform activity spreads to the contralateral side (Figure 3; subsection “Modification of the intrahippocampal KA mouse model”) and is as efficiently suppressed by oLFS as in the ipsilateral hippocampus (Figure 4—figure supplement 1; subsection “Application of different oLFS protocols during spontaneous epileptiform activity”).

8) There are serious concerns with the current form of the manuscript specifically with the translational interpretation of the results. The techniques, data findings, and concepts in this manuscript are seemingly being proposed as a viable alternative to the hippocampal anterior horn resection surgery (anatomically ventral lobe of mouse hippocampus) for patients with refractory MTLE. Primary evidence is not provided to explain why the authors assume seizure recurrence rates would be lower with the optogenetic cell-type specific stimulation. Additionally, first generation deep brain stimulation paradigms that are open loop are antiquated as they lack long-term efficacy. In this regard, closed-loop (on demand) deep brain stimulation in conjunction with resective surgeries are already in practice and report benefits for cases where the epileptic focus is not discrete nor amenable to complete resection (Ma et al., 2020). Resective epilepsy surgery provides seizure freedom to the majority of patients with pharmacoresistant focal MTLE (please see review by Englot and Chang, 2014). Early surgical intervention is a predictor for improved seizure freedom for patients with temporal lobe epilepsy (Engel et al., 2003; Wiebe, 2001). Patients with bilateral epileptic foci, or who are at risk for severe memory impairments, may not be ideal candidates for surgical resection. Deep brain stimulation is one alternative surgical therapy for these patients. The optimal anti-ictogenic stimulation parameters for DBS in MTLE have not been identified/there is no clear consensus, however multiple surgery centers are already reporting efficacy results for closed-loop DBS in patients. The current manuscript is not positioned to compare the efficacy between pharmacological intervention, surgical resection, and DBS stimulation (either electrical or optogenetic) for these reasons:

We follow the reviewer's opinion, that the translational interpretation of the results was too strong in the former manuscript. We appreciate the detailed suggestions of the reviewer and changed the interpretation of our results accordingly (subsection “oLFS of entorhinal afferents interferes with spontaneous, recurrent seizures”). We would like to emphasize that LFS suppressed epileptiform activity in both hippocampi during 1 Hz stimulation. We think this an important result, since “Patients with bilateral epileptic foci, or who are at risk for severe memory impairments, may not be ideal candidates for surgical resection. Deep brain stimulation is one alternative surgical therapy for these patients.” We agree that the former manuscript did not give enough information about the long-lasting effect of LFS. However, in the revised version, we show new eLFS data over several hours and weeks (subsection “Effects of repeated hippocampal eLFS on epileptiform activity*”*). We also show that the seizure-suppression is maintained in short stimulation-free phases. This new data might be valuable to implement a novel LFS-based closed-loop system (subsection “The anti-epileptic effect of eLFS is stable over several weeks”).

A) For translational relevance as a therapeutic intervention pharmacoresistance should first be demonstrated in this model, with refractoriness to anti-seizure drugs quantified with the similar parameters reported here (e.g. High load burst ratio, Epileptic spike rate, seizure probability, etc.). Without this essential data the manuscript does not need to claim a translational relevance (see Abstract) nor suggest an improved outcome of oLFS vs. all other therapeutic interventions. None of these statements are direclty supported by any data in the manuscript. Abstract "As an alternative to curative epilepsy surgery, brain stimulation evolves as a promising approach for seizure-interference." Electrical deep brain stimulation is a type of epilepsy surgery and should be portrayed as such. Abstract "exhibits unprecedented anti-ictogenic effects in chronically epileptic mice." It is unclear what oLFS efficacy is being clinically compared to throughout the manuscript. Is it resective epilepsy surgery, high frequency deep brain stimulation, or pharmacoresistance? Introduction "However, many patients do not remain seizure-free following curative epilepsy surgery (Mohan et al., 2018; Ryvlin and Kahane, 2005), thus demonstrating the urgent need for new therapeutic avenues." This statement is not supported by the current citations. It would benefit the reader to cite robust Class I evidence or meta-analyses data specifically investigating long-term outcomes of resective epilepsy surgery in MTLE. All assessments of clinical data should provide, at the minimum, the rate of seizure freedom and time post-surgery (e.g. 50-75% seizure free at 1 year). Introduction "However, in MTLE with severe hippocampal sclerosis, electrical stimulation appears remarkably ineffective…(Velasco et al., 2007)" It is not clear what the efficacy of electrical stimulation in MTLE is being compared to. Velasco et al., 2007 does not support the above statement. Rather, the conclusions of Velasco et al., 2007 are much more nuanced as stated by the authors in the Discussion. Discussion – here the authors portray the potential translational utility for this data in a weighted and clear manner, in contrast to the Introduction.

We agree with the reviewer's suggestions and revised major parts of the manuscript. We rephrased potentially misleading parts in the Abstract, Introduction and the Discussion. We removed statements that are not directly supported by our data and critically discussed our results in the context of the current literature.

B) It is important to note that all interventions reported in this manuscript are acute time points, and all comparisons to clinical data are invalid as these outcomes are at much longer time scales (many months). Most anti-seizure drugs if tested would be effective during the sub-acute temporal periods chosen for the recordings. At the minimum, the limitation of acute stimulation parameters should be discussed.

We addressed this important point by conducting eLFS experiments for three weeks (see above point 1). Importantly, we could not find any indication for the development of tolerance, a problem that can occur with AEDs (Löscher and Schmidt, 2006). We refer to this point in the Discussion of the revised manuscript. Additionally, we removed all comparisons to clinical data, which are not supported by our data.

9) Bilateral hippocampus

It is unclear if the bilateral hippocampus exhibits spontaneous discharges in this model. Figure 3A-B shows a dose dependent generalization of epileptiform activity into the contralateral dorsal hippocampus, but there is no quantification of this important parameter in Figure 3, Figure 4, Figure 5. Does this model involve the kindling of the contralateral hippocampus over time with the 15mM KA dose? Does idHC oLFS suppress cdHC epileptiform activity? Are there any KA dose differences? This quantification is important as bilateral epileptic foci are an important parameter for MTLE DBS candidates.

Thank you for this comment! In the revised version, we included an analysis of contralateral epileptiform activity (Figure 3) (see also point 7). Interestingly, we see a dose-dependent propagation of epileptiform activity into the contralateral dorsal hippocampus (subsection; subsection “Modification of a mouse epilepsy model to obtain variable degrees of hippocampal sclerosis and seizure burden”). This propagation was stronger in animals treated with lower KA concentrations (Figure 3C). We did not observe a kindling effect of the contralateral hippocampus over time. We fully agree with the reviewer’s remark, that the quantification of contralateral activity is important, as bilateral epileptic foci are an important parameter for MTLE DBS candidates. In fact, oLFS in the idHC also suppressed cdHC epileptiform activity (see point 7).

10) Cell-type specificity

Introduction The authors state that the stimulation of interneurons in preclinical mTLE increases seizure probability (Lévesque et al., 2019). This citation is from a systemic PTZ model with different stimulation parameters. The authors should either demonstrate the inability of optogenetic activation of interneurons to prevent ictogenesis in their model (potentially via the loss of GABAergic interneurons etc.) or remove statements claiming that targeting interneurons are ineffective as further studies are required in this model. Further, it would benefit readers to delineate stimulation differences when referencing other clinical or preclinical work (i.e. closed vs. open loop).

We understand the reviewer’s concern and changed the revised manuscript accordingly. The work of Lévesque et al., (2019) is not cited anymore, since it was conducted in a different model and different stimulation parameters, as pointed out by the reviewer. We critically revised the manuscript and removed statements claiming that targeting interneurons are ineffective but we note that they would be difficult to target (Introduction). We delineated stimulation differences when referencing other clinical or preclinical work (Introduction; subsection “oLFS of entorhinal afferents interferes with spontaneous, recurrent seizures”).

11) Frequency dependency

From the current manuscript it is clear that there are frequency dependent anti-seizure differences. However, low frequency stimulation is performed because the authors state that high frequency stimulation is ineffective. The authors should either demonstrate the inability of high frequency stimulation to prevent ictogenesis in their model, or remove statements claiming that they are ineffective.

In the revised manuscript, we discussed our results more carefully and removed statements claiming that high-frequency stimulation is ineffective for the prevention of ictogenesis in our model. We rather present LFS as an alternative treatment for those patients that are neither amendable to surgical resection nor respond to HFS and accentuate the putative technical benefits of LFS (Introduction).

12) Closed loop neuromodulation

Open-loop continuous stimulation is prone to failure over time as the approach does adapt to natural fluctuations, disease progression, or nervous system adaptations. In contrast, closed-loop neurostimulation is a rapidly emerging technology positioned to overcome the shortcomings of open-loop continuous stimulation. Therefore, brain responsive closed-loop neurostimulation (RNS System) devices should be extensively discussed in the context of this current manuscript (Geller et al., 2017and Van Ness et al., 2016). Previous closed-loop preclinical studies utilizing optogenetics in a unilateral KA model have demonstrated that on-demand inhibition of DGCs is anti-ictogenic (Krook-Magnuson et al., 2015; Krook-Magnuson et al., 2013), discussing this work in detail would benefit the reader.

We agree with the reviewer that closed-loop stimulation has several advantages over open-loop approaches. In the present study, open-loop stimulation was highly efficient in suppressing epileptiform activity. However, in the course of the revision, we collected new evidence that 1 Hz LFS is suited for a closed-loop stimulation approach since mice experience seizure-free periods after LFS (Figure 8K-L). We propose in the revised discussion that closed-loop stimulation would be a great improvement and could be implemented in our setup (subsection “The anti-epileptic effect of eLFS is stable over several weeks”). In addition, we discuss brain responsive closed-loop neurostimulation (RNS System) (Introduction) and previous closed-loop preclinical studies utilizing optogenetics in our revised manuscript (subsection “Preconditioning by LFS prevents induced generalized seizures”).

13) The authors do not address or nor justify the exclusion of female mice in this study.

In the present study, we did not use female mice. Therefore, we cannot exclude sex-dependent effects. However, we like to note that Zeidler et al., (2018) and Kim et al., (2020) injected KA into the dorsal hippocampus in male and female mice and observed no significant gender differences. Hence, we expect that our stimulation paradigm would also be effective in female mice.

14) It is important to note that this paper uses different types of stimulation, which are not entirely well described in the paper (as dentate granule cell stimulation in the title). I think this latter phrasing is a little misleading. The light stimulation is firstly done as a stimulation of MEC fibers expressing ChR2 within the dentate gyrus. This is important, because even if the light stimulation is local within the dentate gyrus, retrograde firing of EC neurons will potentially lead to more wide-spread network effects. This should be acknowledged and discussed.

We agree with the reviewer’s remark. Therefore, we described the applied stimulation techniques in more detail (subsection “in vivo LFS experiments) and changed the title of the paper accordingly. In addition, we mentioned potential effects due to retrograde firing of EC neurons in the revised manuscript (subsection “LFS 488 reduces the efficacy of perforant path transmission onto DGCs”).

Secondly, a more direct stimulation of neurons within the dentate gyrus is achieved with injection of virus at the same location as the kainate injection. However, also this viral transduction technique is not specific to DGCs, as the authors suggest at several points. The AAV used has a CaMKIIa promoter, which will express not only in DG neurons, but potentially also in other types of neurons (i.e. adjacent CA3 cells, potentially mossy cells, CA1 neurons), in particular since the injection location for kainate as far as I interpret the coordinates is not specifically located within the DG. Because light delivery is likewise never very specific, this means that the authors may stimulate a much more distributed hippocampal cell population. The figure panel 5B looks consistent with granule cell expression, but does not really give a precise idea of non-granule cell expression, in particular in the hilus and CA3 areas. There must be an analysis of expression patterns of hippocampal ChR2 using post-hoc immunohistochemistry, with a quantification of neurons in dentate, hilus and CA3/1 areas for all animals to be able to interpret the data clearly.

To address this concern, we removed the data concerning the direct application of the virus to DGCs combined with kainate injection at the same location (previous Figure 5) since we realized that the hippocampus was severely damaged by this procedure. Instead, we added new data concerning eLFS, which is of greater translational value (see above Points 1 and 4).

Regarding the MEC virus injections, the MEC is not easy to target – the authors should demonstrate that they indeed target MEC in all animals by quantifying the expected band of mCherry positive fibers in the middle molecular layer of the dentate gyrus in those animals included in the study. Given the importance of this issue, I think it would be good to show more definitive quantification than the example shown in Figure 4C (which does look convincing).

Targeting the medial entorhinal cortex (MEC) has been implemented before in our laboratory (Janz et al., 2018, 2017) and presents a robust technique to label entorhinal afferents in the mid molecular layer of the dentate gyrus. Nevertheless, we addressed the comment of the reviewer and now present quantitative data concerning the ChR2-mCherry-positive fibers in the middle molecular layer of the dentate gyrus in all animals included in the study. We densitometrically analyzed the gray values within the different hippocampal layers along the dorsoventral axis at the position of the optic fiber to confirm ChR2-mCherry-expression in MEC projections to the dentate (subsection “Image acquisition and analysis”; Figure 4—figure supplement 2).

Given that the authors are saying that this may be a promising avenue for treatment, I think it is important to get a better sense of what is actually being stimulated in these experiments. This is particularly true given that in the Krook-Manguson, 2015 paper , selective expression of NpHR and ChR2 only in granule cells has been done. In these cases, stimulation of granule cells has resulted in induction of seizures and increased seizure progression to large behavioral seizures. This is even consistent with the authors data from figure 6 in my view, and I was really surprised that the authors have not discussed this. Does this not support the idea that the key factor deciding on seizure generation or reduction is stimulation frequency? This is also relevant for translation.

I also think that the paper by Bui et al., (2018) should be cited, this is a pretty glaring omission, as this paper has studied the effects of selectively manipulating mossy cells on seizures in a temporal lobe epilepsy, a cell type that is present within the dentate hilus and is known to strongly affect excitability of granule cells. This is clearly very relevant to the present paper. Incidentally, this paper also contains a figure of on-demand inhibition of dentate granule cells (Supplementary figure 11), which strongly truncates seizures. In the context of the authors hypothesis of how LFS works, this is also clearly relevant.

We addressed the points raised by the reviewer by including a more detailed discussion of stimulation frequency and timing (subsection “Preconditioning by LFS prevents induced generalized seizures”) and now refer to Bui et al., (2018).

15) Materials and methods: Results for ANOVA should be reported fully according to established guidelines (i.e. APA guidelines), throughout the manuscript. In addition, it should always be clear what the p-values refer to (i.e. in subsection “Variability of hippocampal sclerosis and seizure burden in chronically epileptic mice” a p-value is given for Figure 2B, but it is not explicit if this is for the ANOVA or one of the post tests.

A full report of ANOVA results and statistics are now added in Supplementary file 1.

There should be at least a brief account of how the analysis of the burst events was done in the Results section that allows the reader to get an idea of what these classifications mainly report. It is somewhat irksome to read the results, refer to the methods, which also does not carry more information about this classification, and instead refers to a prior paper (Heining et al., 2019). If I understood it correctly, the recording sites in the Heining et al., paper were identical to those analyzed in this paper (this should be mentioned explicitly), but the injections were confined to 20 mM kainate in the previous paper. I assume that the authors have considered these following issues, but it is not clear from the manuscript. Firstly, one would like to see the SOMs that are the basis of the classification at least in some examples to see how well this discrimination works for the more subtle variant of this model. Also, it is unclear to me how exactly the classification will be comparable between the different kainate model variants. The SOM may classify into three categories in all three models, but in this case the average spike load indexes for the three categories might be different in the different epilepsy models. At this point it is not very easy for me to see what the differences in the composition of burst classes actually means when translated into firing dynamics in the different systems. I do appreciate the importance at looking at structure in high-dimensional data using such methods, but I think the authors could do a much better job at explaining this. I also think the authors should compute, and give some basic statistics of spiking in the different models (frequencies, inter-event interval distributions, etc.). This may not be the best way to show differences between models, but I do think it is important to evaluate the performance of the SOM protocol.

We agree, that the analysis of the burst events has not been adequately described. In the revised manuscript, we added a description of the code in the Results section (subsection “Modification of the intrahippocampal KA mouse model) and a more detailed description in subsection under “Analysis of epileptiform activity”. Moreover, we added a Figure (Figure 3—figure supplement 1) that visualizes the workflow of the burst classification and show that it is comparable between the different kainate concentrations. Our collaborators are currently preparing a manuscript describing the software and its performance in detail. The code will be made publicly available via Git upon acceptance of the manuscript. We will then update the current publication with a link to the repository.

16) The slice recordings, together with the in-vivo opto-LFP recordings support a decrease in cellular excitability following light stimulation. This is interesting, but I think preliminary. One of the key findings in-vivo is that 1Hz stimulation induces a lasting effect. Therefore, one would have liked to see some data on whether this is a persistent effect, that outlasts the effects of the optical stimulation. It is not per se surprising that there is a decrease in neurotransmission upon prolonged afferent stimulation. Do the authors have some idea of what happens after the stimulus train? They probably have at least a subset of neurons that they were able to continue measuring after the stimulation train.

We appreciate the reviewer´s comments and understand that deeper knowledge about the excitability of DGCs after oLFS would be helpful to understand the observed anti-epileptic effects. Our original approach was indeed to investigate the long-term effects of oLFS using whole-cell recordings in vitro. Regrettably, we observed a slow but constant decline in input resistance after starting whole-cell recordings, which was independent on the oLFS stimulation, and decided, therefore, to switch either to very short patch-clamp or to cell-attached recordings. Nevertheless, we report here data on the same cells showing that after oLFS stimulation synaptic transmission (EPSP amplitude) goes back to control values after app. 30 min (from -76.2 ± 21.0 to -72.0 ± 20.3 pA, 28.4 min after 10 min 1 Hz photostimulation, n = 13, p = 0.58, paired t-test).

[Editors' note: further revisions were suggested prior to acceptance, as described below.]

Essential revisions:

Please see the detailed comments for your information below. The editors and reviewers agree that the following two issues need to be addressed. It is expected this should not require any additional experiments. First, please modify the text to remove any remaining claims that are not supported by statistical analysis. We think the best approach is simply to provide the quantified population results and the statistical test results. Secondly, there remains an issue of specificity of pathway stimulation. It is not entirely clear that the optogenetic approach is only affecting the MEC to DG circuit in vivo. The authors rely only on staining in the DG molecular layer as evidence of specificity. However, given the anatomy of the entorhinal-hippocampal circuit and the experimental design to target dorsal blade of DG, it would really help to show that stimulation produces no response in CA1. Alternatively, please provide images of sagittal sections documenting that virus injection was restricted to MEC, as is standard practice for these types of experiments.

We are happy that all reviewers were satisfied with the substantially revised manuscript including additional experiments. For the second revision, we addressed clarity and presentation as pointed out by the editor.

First, we modified the text and removed any remaining claims that are not supported by statistical analysis. We now report quantified population results and the statistical tests for the suppression efficacy of high-load bursts and epileptic spikes in the ipsilateral (Figure 4P, Q) and contralateral (Figure 4—figure supplement 1P, Q) hippocampus. We further added a comparative analysis of the different suppression efficacies (1, 0.5 and 0.2 Hz). This showed that 1 Hz oLFS can be considered as the most successful stimulation frequency (subsection “Application of different oLFS protocols during spontaneous epileptiform activity”).

Secondly, we agree that in the current manuscript we cannot prove that the optogenetic approach is merely affecting the MEC to DG circuit in vivo. However, sagittal sections of anterogradely traced medial perforant path fibers, that show the preserved medial perforant path and the degeneration of the temporoammonic path, were published previously (Figure 2 in Janz et al., 2017). In the current manuscript, we used the same medial entorhinal cortex coordinates for virus injections. Since we cannot prove the specificity of perforant path stimulation, in the revised manuscript we removed all statements concerning specific perforant path stimulation in vivo and state instead that we stimulated remaining entorhinal afferents in the sclerotic hippocampus (Introduction; subsection “oLFS of entorhinal afferents interferes with spontaneous epileptiform activity”).
